# Revisiting the photochemical synthesis of [FeFe]-hydrogenase mimics: reaction optimization, mechanistic study and electrochemical behaviour†

**DOI:** 10.1039/d0ra06002j

**Published:** 2020-08-12

**Authors:** Sergio Aguado, Luis Casarrubios, Carmen Ramírez de Arellano, Miguel A. Sierra

**Affiliations:** Departamento de Química Orgánica, Facultad de Ciencias Químicas, Center for Innovation in Advanced Chemistry (ORFEO-CINQA), Universidad Complutense 28040-Madrid Spain sierraor@ucm.es lcasarru@ucm.es; Departamento de Química Orgánica, Center for Innovation in Advanced Chemistry (ORFEO-CINQA), Universidad de Valencia 46100-Valencia Spain

## Abstract

The photoreaction of [(μ-S)_2_Fe_2_(CO)_6_] and alkenes or alkynes has been optimized to readily obtain functionalized [FeFe]-hydrogenase mimics. Irradiation under low CO pressure in THF produces the corresponding photo-adducts in good/acceptable (alkenes/alkynes) yields, with retention of the starting olefin stereochemistry. DFT-calculations provide plausible reaction pathways in both, singlet and triplet states. The DFT-calculation based in the singlet state is energetically more favorable. The electrochemical behavior of the synthesized compounds is also presented, including studies in acidic media. The electrochemical properties of the products vary in the presence of a double bond (cycloaddition of [(μ-S)_2_Fe_2_(CO)_6_] to alkynes), respect to a single bond (cycloaddition to alkenes).

## Abstract 2

The photochemistry of [(μ-S)_2_Fe_2_(CO)_6_] and alkenes or alkynes has been developed to a synthetically useful level. Computational data and electrochemistry of the photoadducts show interesting properties of these compounds.

## Graphical abstract

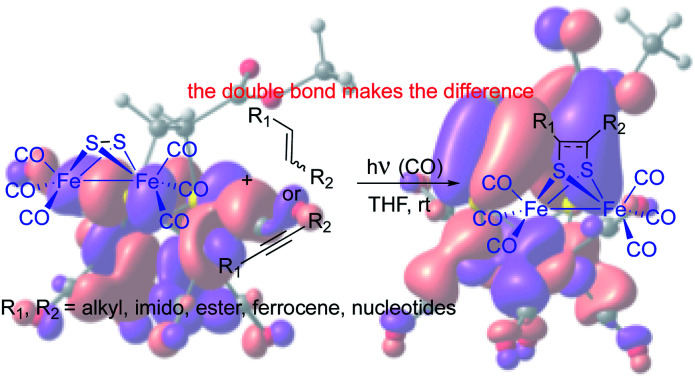

## Introduction

One of the challenges of chemistry in the 21st century is to develop viable methods to produce hydrogen.^1^ Hydrogenases are metalloenzymes able to generate molecular hydrogen by reducing protons in anaerobic media.^2^ The use of hydrogenase bio-inspired analogues is a promising option for the production of hydrogen. Current research in this field is mainly focused on two different approaches. The first one uses whole organisms, inorganic hybrids or supported enzymatic systems.^3^ The second approach uses mimics of hydrogenases, namely synthetic small molecules that when coupled to other reagents and catalysts are able to produce hydrogen.^4^

**Fig. 1:**
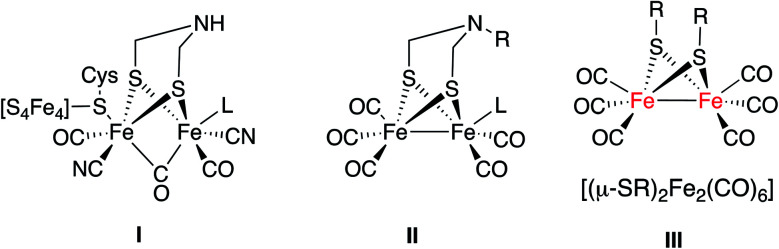
Schematic representation of [FeFe]-H_2_ase active site (I) and their synthetic mimics (II and III).

Fragment I is the basic motif of the hydrogen production moiety of a [FeFe]-hydrogenase (Fig. 1). Much synthetic research in this field targeted the preparation of mimics with a simplified structural motif II similar to I.^4*b*^ A second group of [FeFe]-H_2_ase synthetic models has the [(μ-SR)_2_Fe_2_(CO)_6_] motif as the essential core (III in Fig. 1).^4*a*^ This second group of mimics has been less studied in the photocatalytic production of hydrogen.^4*c*^

**Scheme 1:**
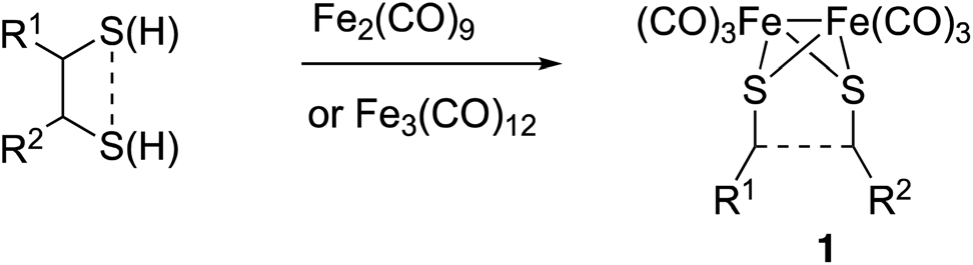
Synthesis of [FeFe]-H_2_ase synthetic models.

The preparation of type III mimics is achieved by thermal reaction of either Fe_2_(CO)_9_ or Fe_3_(CO)_12_ and sulphides or disulphides to yield compounds having structure 1 (Scheme 1).^4*b*^ This approach is versatile and provides access to sophisticated structures. However, again the reaction conditions are not tolerated by several classes of substrates. Additionally, the precursors of the sulphides or disulphides are not always easy to access.^5^

**Scheme 2:**
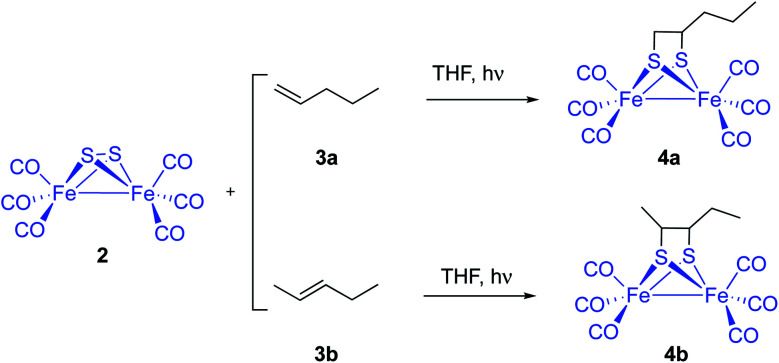
Photocycloaddition of 2 with 1- and 2-pentene 3a and 3b.

An alternative and potentially useful approach to introduce the [(μ-S)_2_Fe_2_(CO)_6_] 2 moiety into substrates not compatible with the conditions used by standard approaches would be the photocycloaddition of [(μ-S)_2_Fe_2_(CO)_6_] and alkenes or alkynes. The photochemical reaction of 2 and simple unfunctionalized substrates has been previously reported. However, yields of photoreactions are usually very poor. Thus, irradiation of [(μ-S)_2_Fe_2_(CO)_6_] 2 and simple olefins^6^ including 1- and 2-pentene 3a and 3b yielded the corresponding photoadducts 4a and 4b in 6.9% and 8.9% yield, respectively (Scheme 2).

Similar low yields were obtained with both, acyclic^6^ and cyclic^7^ dienes. The only exceptions are ethylene^8^ and *p*-benzoquinone,^9^ that produce the corresponding photoadducts in 65% and 53% yields, respectively. Finally, several C_60_[S_2_Fe_2_(CO)_6_]_*n*_ (*n* = 1–6) and C_70_[S_2_Fe_2_(CO)_6_]_*n*_ (*n* = 1–4) mixtures were obtained from [(μ-S)_2_Fe_2_(CO)_6_] and C60 and C70 fullerenes. The C_60_[S_2_Fe_2_(CO)_6_] adduct was separated from the mixture with a 52% yield based on recovered C60, while C_70_[S_2_Fe_2_(CO)_6_] adduct was obtained with a 63% based on recovered C70.^10^ The mechanism of these photoreactions remains unexplored. The mechanisms and synthetic applications for organometallic compounds photochemistry are intrinsically different from their all-carbon counterparts, being a subject of general interest.^11^

Despite the reported low yields for the photocycloaddition of [(μ-S)_2_Fe_2_(CO)_6_] and alkenes/alkynes, this reaction may be a good alternative to include this [FeFe] moiety into substrates incompatible with the reaction condition used by other synthetic approaches to these classes of compounds. We report herein a useful optimized approach to incorporate the [(μ-S)_2_Fe_2_(CO)_6_] into smooth reaction conditions to different classes of substrates, as well as a proposal for the reaction mechanism using DFT calculations.

## Results and discussion

**Scheme 3:**
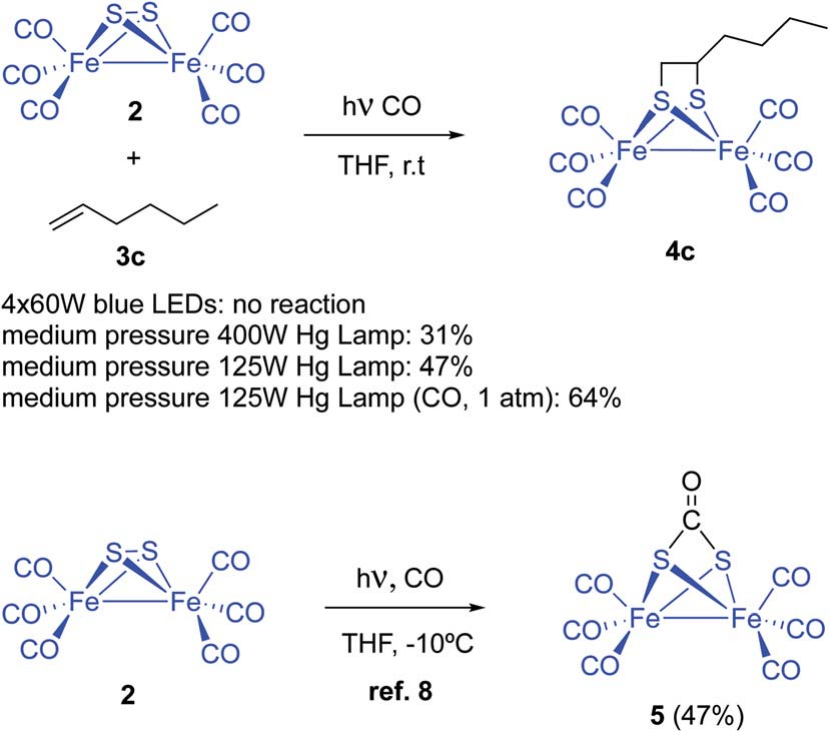
Photochemical reaction between [(μ-S)_2_Fe_2_(CO)_6_] 2 and olefins or alkynes 3. Initial optimizations.

Complex [(μ-S)_2_Fe_2_(CO)_6_] 2 and 1-hexene were used to tune up the reaction conditions. Light source and solvent were first investigated. Thus, irradiation of equimolar amounts of [(μ-S)_2_Fe_2_(CO)_6_] and 1-hexene in anhydrous THF using 4 × 60 W blue light LEDs did not produce any reaction product. The reagents were recovered unaltered after 72 hours of irradiation. The use of medium pressure Hg-lamps (Pyrex filter and Pyrex well) produced the desired photoadduct 4c in 31% (400 W) and 47% (125 W) isolated yields. A 6.9% yield for the reaction of 1-pentene and [(μ-S)_2_Fe_2_(CO)_6_], using a high-pressure Hg-lamp and quartz glassware, was previously reported. Thus, filtering the UV component of the irradiation source clearly increases the reaction yield. This yield improvement is probably due to a smaller decomposition of the diiron complexes by the CO-ligands photo-removing effect (see below). Other solvents like MeCN (14%), benzene (18%), and Et_2_O (11%) produced lower isolated yields of the adduct 4c (Scheme 3).

Dependence of yields with the choice of solvent pointed to a competitive light-induced CO dissociation leading to, either decomposition or tetrameric species.^12^ Thus, THF would fill iron coordination vacants avoiding or retarding competitive undesired reactions. This hypothesis would imply a yield increment under CO-atmosphere. However, it has been reported that complex [(μ-S)_2_Fe_2_(CO)_6_] reacts with CO to form the CO adduct 5 with a 47% yield (Scheme 3).^8^ Nevertheless, the reaction of [(μ-S)_2_Fe_2_(CO)_6_] 2 and 1-hexene was repeated under 1 atm (14 psi) of CO and, compound 4c was obtained with a 64% isolated yield. The reaction crude material was cleaner and decomposition of the starting diiron complex 2 was not observed. Therefore, it is clear that CO atmosphere hampers the photo-extrusion of CO and thence the decomposition of the [(μ-S)_2_Fe_2_(CO)_6_], increasing the reaction yields. However, the use of higher pressures of CO (40 psi) resulted in lower yields of the desired product. Competitive CO insertion to produce 5 might be the cause of these lower yields.^8^

**Scheme 4:**
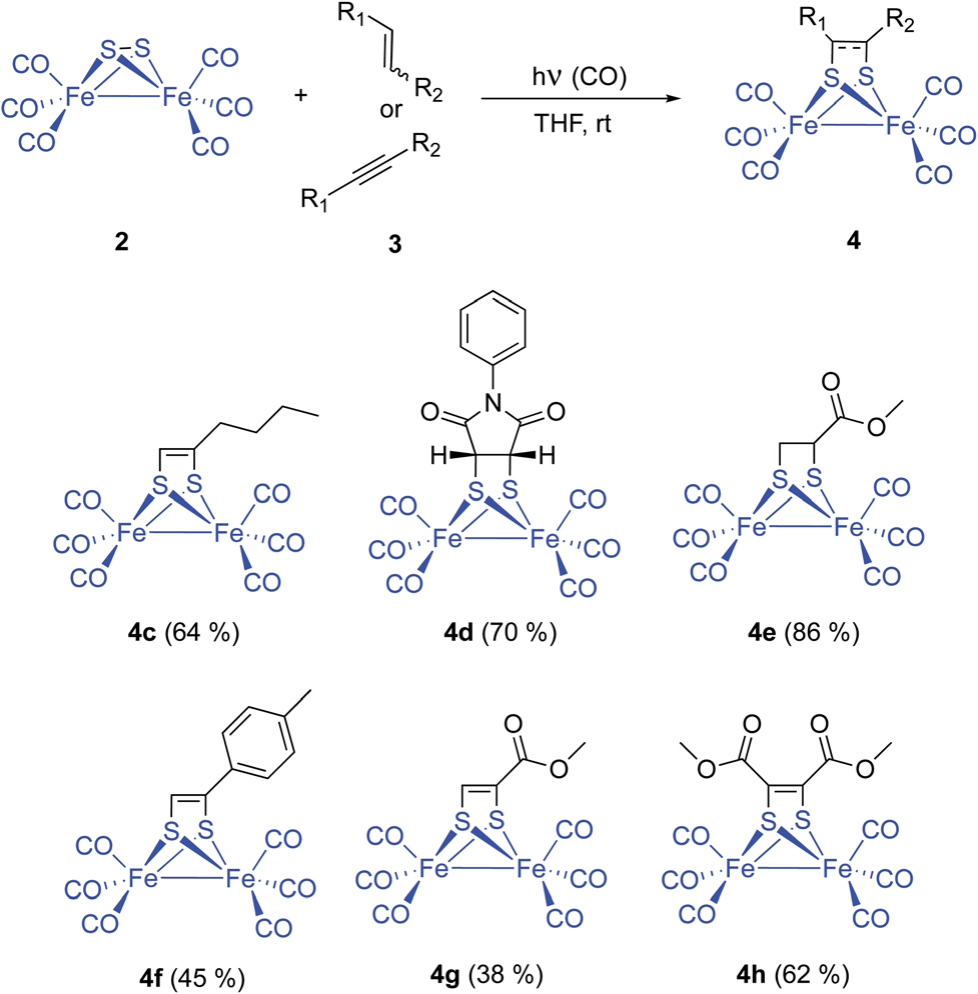
Photochemical reaction of [(μ-S)_2_Fe_2_(CO)_6_] with olefins and alkynes. Substrate scope.

Fine tuning of the reaction conditions of this photocycloaddition allows a yield increment from the described 6–9% up to 65% in the case of simple aliphatic olefins. Functionalized alkenes like *N*-phenylmaleimide 3d and methyl acrylate 3e were reacted with [(μ-S)_2_Fe_2_(CO)_6_] 2 to form the corresponding photoadducts 4d and 4e with 70% and 86% isolated yields, respectively. These yields were achieved with THF as the choice solvent under 1 atm of CO and a 125 W medium pressure Hg-lamp (Pyrex filter and Pyrex well) (Scheme 4).

Series of both, terminal and disubstituted alkynes were next tested as starting substrates. *p*-Tolyl acetylene 3f formed the corresponding adduct 4f in 45% yield, while methyl propyolate 3g and dimethyl acetylendicarboxylate 3h yielded the corresponding adducts 4g (38%) and 4h (62%). Although functionalized alkynes formed the corresponding cycloadducts in lower yields than those obtained for alkenes, they could be used as substrates for the cycloaddition process. Therefore, the method is general and tolerates a variety of functional groups (*vide infra*).^13^

**Fig. 2:**
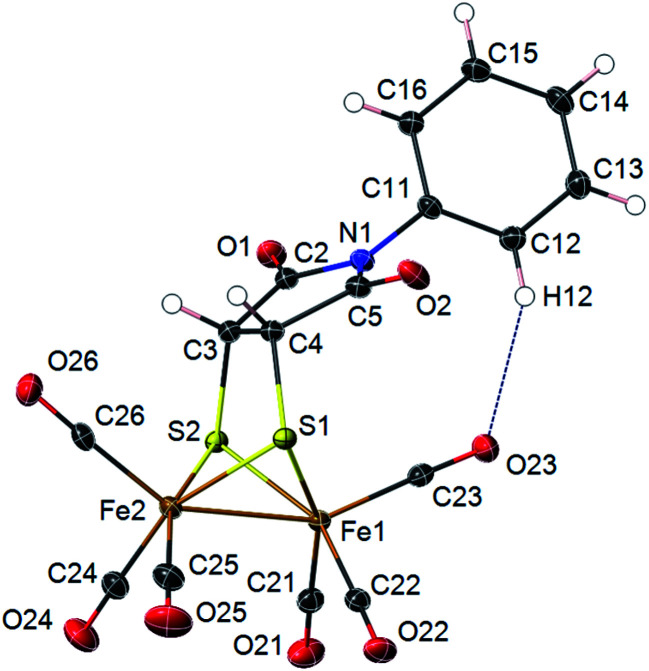
X-ray thermal ellipsoid plot for compound 4d (50% probability level) showing a C–H⋯OC intramolecular interaction. Selected bond lengths (Å) and angles (°): Fe(1)–Fe(2) 2.4966(3), Fe(1)–C(21) 1.7983(15), Fe(1)–C(22) 1.7990(15), Fe(1)–C(23) 1.8102(15), Fe(1)–S(1) 2.2428(4), Fe(1)–S(2) 2.2473(4), Fe(2)–C(24) 1.7995(16), Fe(2)–C(25) 1.8004(16), Fe(2)–C(26) 1.8077(15), Fe(2)–S(1) 2.2521(4), Fe(2)–S(2) 2.2513(4), S(1)–C(4) 1.8289(4), S(2)–C(3) 1.8434(4), C(3)–C(4) 1.5203(18), S(1)–Fe(1)–S(2) 81.488(14), S(1)–Fe(2)–S(2) 81.197(13), Fe(1)–S(1)–Fe(2) 67.478(12), Fe(1)–S(2)–Fe(2) 67.416(12), C(31)–N(1)–C(2) 119.64(10), C(31), C(12)⋯O(23) 3.310(2) Å; H(12)⋯O(23) 2.45 Å; C(12)–H(12)⋯O(23) 150.7°.

Photoadducts derived from the reaction of 2 with olefins could be formed either as *cis*- or *trans-*isomers in the newly formed metallacycle. The symmetry of our molecules avoids the assignation of the *cis*–*trans* stereochemistry by conventional NMR techniques. Crystals of compound 4d suitable for X-ray diffraction were grown from a DCM/hexane solution. The X-ray structure determination of 4d unambiguously confirms the *cis* arrangement of the fused bicyclic system (Fig. 2). Molecular structure of 4d shows a [(μ-SR)_2_Fe_2_(CO)_6_] complex with a butterfly structure for the [2Fe–2S] cluster. Both iron atoms adopt a distorted square-pyramidal geometry. The Fe–Fe bond length (2.4966(3) Å) lies in the range found for similar ethylenedithiolate-hexacarbonyl-di-iron structures^14^ (2.454–2.546 Å). Fe–Fe bond length in compound 4d is shorter than in metalloenzymes Hydrogenase DdI (*ca.* 2.55 Å) or CpI (*ca.* 2.62 Å).^15^ The dithiolate bridging ligand and both iron atoms form two fused five-membered metallocycles with the nitrogen substituent N-Ph group bending towards the Fe(1) atom. This conformation implies short intramolecular distances between the nitrogen atom N(1) and the closest carbonyl group C(23)–O(23) [N(1)⋯C(23) 3.221(2) Å; N(1)⋯O(23) 3.609(7) Å]. An intramolecular C–H⋯OC–Fe interaction is observed [C(12)⋯O(23) 3.310(2) Å; H(12)⋯O(23) 2.45 Å; C(12)–H(12)⋯O(23) 150.7°]. This interaction lies within the expected parameters for an intramolecular C–H⋯OC–Fe hydrogen bond with the oxygen of a terminal carbonyl group acting as a hydrogen bond acceptor (*i.e.* mean values C⋯O 3.50 Å; H⋯O 2.64 Å; C–H⋯O 138.0°).^16^ Interactions between *N*-arene group and the closest carbonyl group has been previously described to produce an enlargement on the C–Fe–Fe angle for the implicated carbonyl group in azadithiolates diirion structures.^5,17^ Thus, in compound 4d the C(23)–Fe(1)–Fe(2) angle is 5.25(5)° larger than the C(26)–Fe(2)–Fe(1) angle.

Substrates having electroactive moieties were next tested. Methyl *trans*-2-ferrocenylacrylate 3i reacts with 2 and the corresponding photo-adduct 4i is isolated in 43% yield. The *trans* stereochemistry of the starting ferrocene derived olefin 3i is again maintained in the final adduct (*δ* = 4.13 and 3.16 ppm, d, *J* = 6.3 Hz for both CH̲–S groups). To confirm that the stereochemistry of the starting material is retained in the photocycloaddition, NOE experiments were performed for complex 4i on a 500 MHz NMR spectrometer. Irradiation of the signal at 3.16 ppm, corresponding to the CH–CO proton, showed a main NOE effect with the proton at 3.98 ppm (substituted Cp ring). This observed NOE effect points to a *trans* relative disposition of the CO_2_Me and the Fc moieties which is in good agreement with the concerted proposed calculated mechanism (see below).

**Scheme 5:**
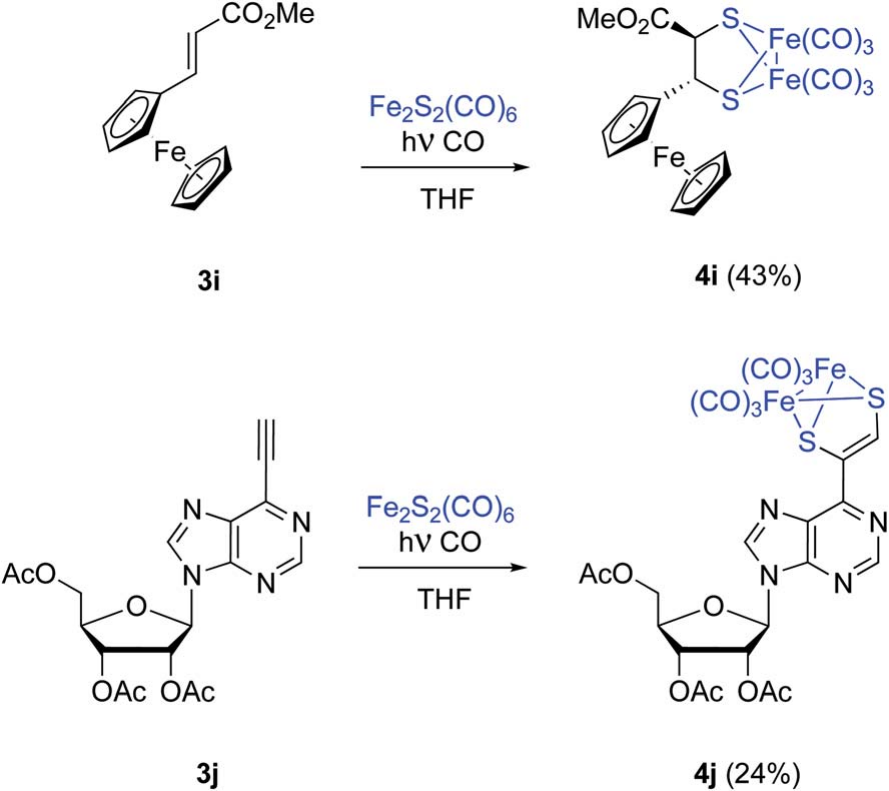
Photochemical reaction between [(μ-S)_2_Fe_2_(CO)_6_] and olefins or alkynes. Compatibility of the process with functionalized substrates. Compound 4i was a racemic material, one single enantiomer is represented for simplicity.

Nucleotide 3j was next tested. In this case the product incorporating the [(μ-S)_2_Fe_2_(CO)_6_] moiety was obtained with a 24% isolated yield. Despite the high functionalization of 3j, no by-products were obtained, and unaltered starting materials could be recovered (Scheme 5).

**Scheme 6:**
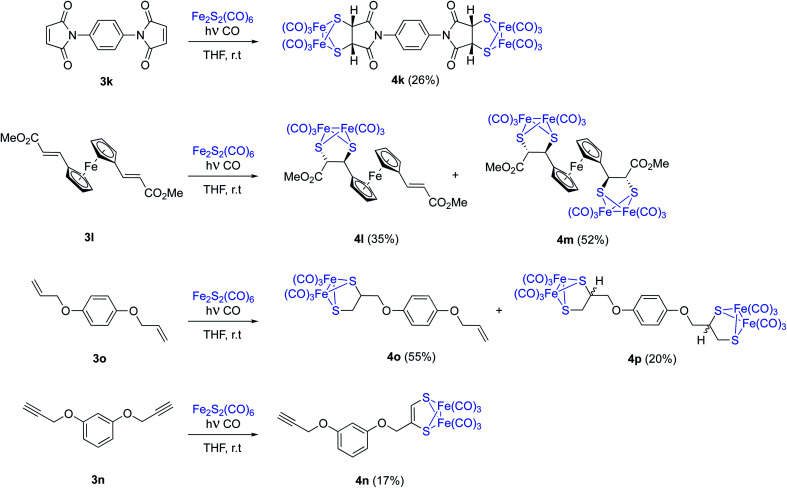
Photochemical reaction between [(μ-S)_2_Fe_2_(CO)_6_] and olefins or alkynes. Synthesis of polymetallic systems. Compounds 4l, 4m were racemic mixtures. Only one enantiomer is depicted for clarity.

The possibility of achieving a double photocycloaddition to obtain tetrametallic systems is also addressed. *N*,*N*′-(1,4-phenylene)dimaleimide 3k reacts with [(μ-S)_2_Fe_2_(CO)_6_], with no further reaction progress observed (tlc) after 15 hours of irradiation. From the crude reaction mixture, tetrametallic complex 4k was obtained with a 26% isolated yield. An analogous reaction was carried out with ferrocene complex 3l. A mixture of pentametallic complex 4m (single diastereomer, 52%) and trimetallic complex 4l (35%) was obtained. It is worthy to note that the four stereogenic centers of complex 4m are formed in a totally stereoselective way maintaining the configuration of the starting olefins (Scheme 6). A high degree of diastereoselectivity has also been achieved in this reaction. An analogous result was obtained from the *bis*-allyl derivative of hydroquinone 3o which lead to a mixture of dimetallic 4o and tetrametallic complex 4p in 55% and 20% isolated yields, respectively. Although the analysis of the crude mixtures of 4p showed a single product, there are no reasons to believe that a complete stereoselectivity was achieved in this case. Probably, 4p is a mixture of diastereomers but the chiral centres are well-separated and the differences in their NMR data may be null. Finally, the *bis*-propargyl derivative of hydroquinone 3n was not able to form the tetrametallic derivative, while bimetallic derivative 4n was obtained with just a 17% yield (Scheme 6). This is in good agreement with the observed lower reactivity of alkynes.

### Mechanistic studies

**Fig. 3:**
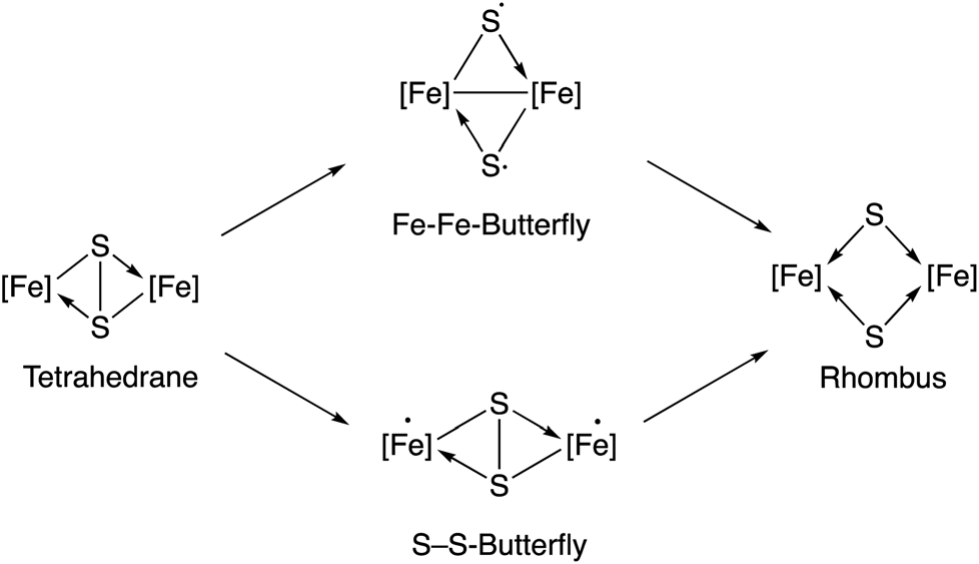
Species implied in the photolysis of complex 2 according to Bruce King *et al.*^18^

According to a previous theoretical study,^18^ irradiation of the starting diiron complex 2 with UV-light would generate two butterfly isomers or a rhombus isomer by breaking one or both of the Fe–Fe and S–S bonds (see Fig. 4 in ref. 18). This study concludes that photochemical reactions of complex 2 should proceed through Fe–Fe butterfly biradical Fe_2_(CO)_6_S_2_ intermediates (Fig. 3).

**Fig. 4:**
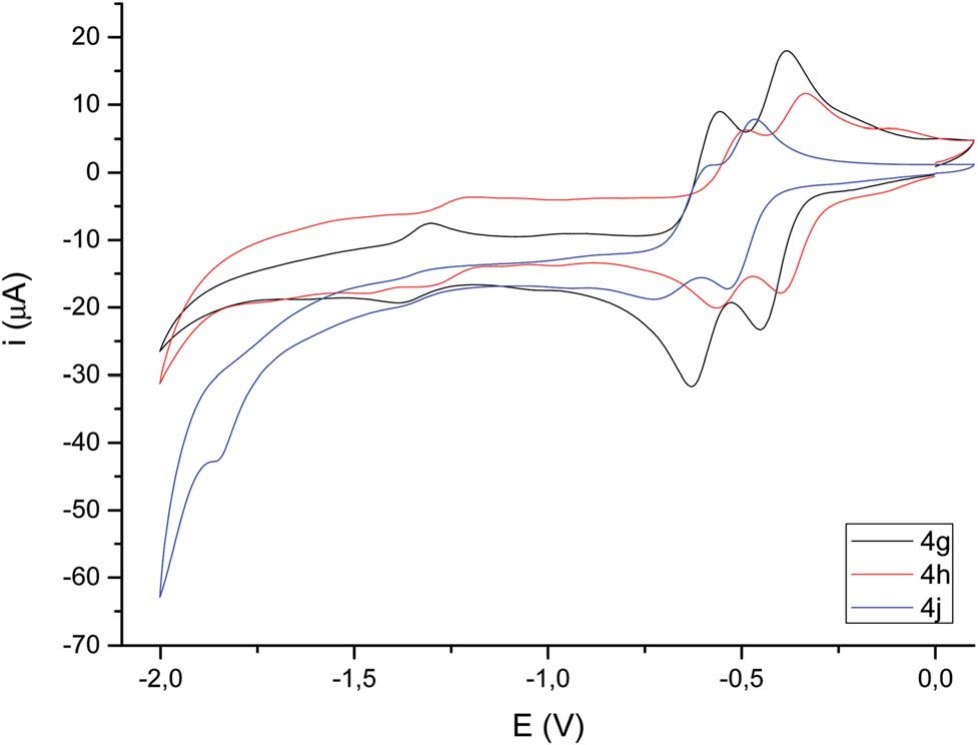
Cyclic voltammograms (focused on reduction) of selected compounds 4c,d (10^−3^ M in CH_3_CN), 10^−1^ M [*N*(^*n*^Bu)_4_]PF_6_, counter-electrode: Pt; working electrode: glassy carbon; reference electrode: Ag/AgCl; scan rate: 100 mV s^−1^; values given in V. For full voltammograms see the ESI.†

**Scheme 7:**
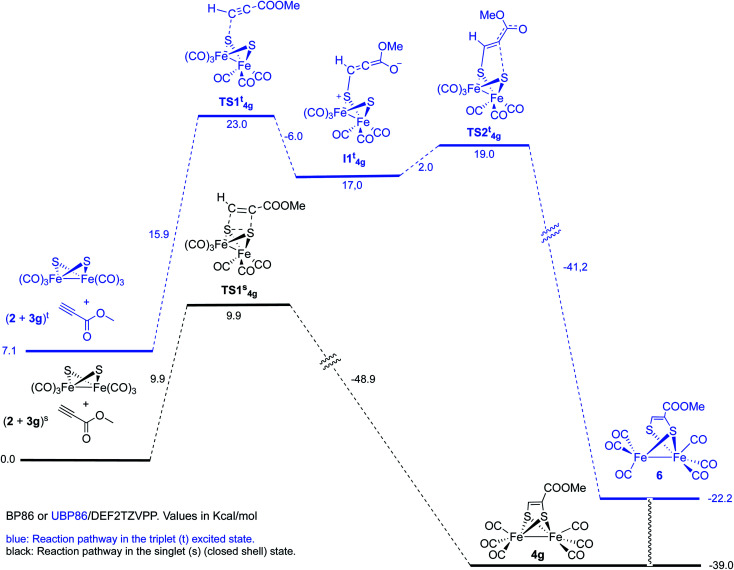
Reaction of complex 2 with methyl propiolate 3g.

**Scheme 8:**
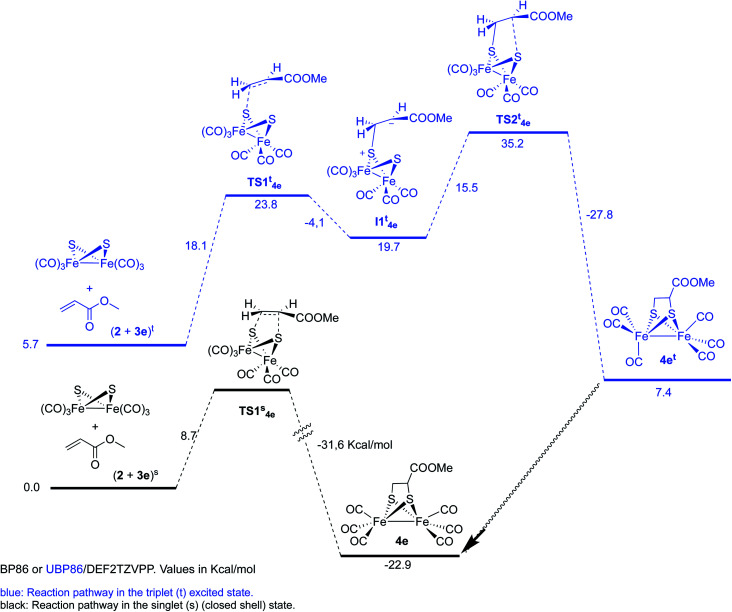
Reaction of complex 2 with methyl acrylate 3e.

However, optimization of the Fe–Fe butterfly using unrestricted uBP86 functional together with the command guess(mix, always) or restricted BP86 yielded the same energy minimum. Careful examination of the spin densities and bond distances in the output files did not match a biradical species in any case. Optimization of the reaction pathway was calculated for the cycloaddition between starting complex 2 and both, methyl propiolate 3g (Scheme 7) and methyl acrylate 3e (Scheme 8).

We first tested the possibility of reacting methyl propiolate with the FeFe-butterfly intermediate in the singlet state. In order to contemplate the possibility of biradicals implied in the process, broken spin symmetry [UBP86 + guess(mix, always)] was compared to restricted RBP86. Both calculations for the first TS of the pathway converged to the same minimum which should, in principle, discard a triplet diradical reaction pathway. Restricted singlet-state calculations for a concerted cycloaddition reaction are shown in Scheme 7. An alternative pathway involving triplet excited states was also contemplated. This process should involve a stepwise cycloaddition having a ΔΔ*G*^‡^ = 30.1 kcal mol^−1^ in the rate-determining step, which makes the process less probable than the concerted pathway (ΔΔ*G*^‡^ = 9.9 kcal mol^−1^). Moreover, the calculated final product 6 for this alternative mechanism has a structure different to the experimentally isolated complex 4g. These species, lacking one Fe–S bond were not observed in any of the experiments carried out in this work.^19^

The reaction of complex 2 and methyl acrylate 3e was also calculated in the singlet and triplet spin states. Results for the singlet state are similar to those obtained for the methyl propiolate. A concerted reaction pathway with a low activation barrier (ΔΔ*G*^‡^ = 8.7 kcal mol^−1^) drives the reaction to the formation of the experimentally isolated product 4e. Unrestricted UBP86 singlet state was also tested and again it converged to the same energy minimum obtained with restricted BP86 one. While the triplet initial state of the reagents was found to be only 5.7 kcal mol^−1^ over the singlet, the two steps process was found to have an overall ΔΔ*G*^‡^ of 35.2 kcal mol^−1^ which makes this process unfavorable when compared to the singlet concerted cycloaddition mechanism (Scheme 8). Therefore, we can safely conclude that the photoreaction of [(μ-S)_2_Fe_2_(CO)_6_] with alkenes and alkynes is a concerted process, which additionally accounts for the observed retention of the stereochemistry of the starting olefins into the obtained final products.^20^

### Electrochemistry

Cyclic voltammograms of compounds 4 were recorded in CH_3_CN solution *vs.* Ag/AgCl (3 M). The reduction of the compounds clearly depends on the presence of a double bond in the bimetallic cycle. Thus, while alkene-derived products (for example 4c–e) show a quasi-reversible reduction wave at about −1.44 V to −1.61 V, assigned to a one-electron [Fe^I^Fe^I^]/[Fe^0^Fe^I^] process,^21^ the analogous alkyne-derived compounds (for example 4f–h) show two quasi-reversible reduction waves about −0.92 V to −1.03 V and −1.08 V to −1.20 V (Fig. 4 and Table 1). Alkyne-derived compounds present a double bond within the metallacycle that is not present for alkene-derived compounds.

**Table 1:** Reduction potentials^a^

	*E*_pc1_	*E*_pa1_ (Δ*E*)	*E*_(1)_	*E*_pc2_	*E*_pa2_ (Δ*E*)	*E*_(2)_	*E*_pc3_	*E*_pa3_ (Δ*E*)	*E*_(3)_
4c	−1.15	−0.96 (0.19)	−1.06	−1.59	—	—	—	—	—
4d	−0.93	−0.84 (0.09)	−0.89	−1.74	−1.64 (0.10)	—	—	—	—
4e	−1.05	−0.89 (0.17)	−0.97	−1.45	—	—	—	—	—
4f	−0.70	−0.60 (0.09)	−0.65	−1.47	—	—	—	—	—
4g	−0.46	−0.39 (0.07)	−0.42	−0.63	−0.56 (0.07)	−0.60	−1.39	−1.30 (0.09)	−1.34
4h	−0.40	−0.34 (0.07)	−0.37	−0.57	−0.49 (0.08)	−0.53	−1.31	—	—
4i	−1.08	−0.87 (0.21)	−0.97	−1.56	—	—	—	—	—
4j	−0.54	−0.47 (0.07)	−0.50	−0.72	−0.59 (0.13)	−0.65	−1.86	—	—
4k	−1.11	−0.82 (0.29)	−0.96	−1.71	—	—	—	—	—
4l	−1.06	−0.91 (0.15)	−0.99	−1.83	—	—	—	—	—
4m	—	—	—	—	—	—	—	—	—
4n	−0.77	−0.60 (0.18)	−0.68	−1.62	—	—	—	—	—
4o	−1.10	−0.91 (0.18)	−1.00	−1.60	—	—	—	—	—
4p	−1.10	−0.99 (0.12)	−1.04	−1.50	—	—	—	—	—

a Data (V) obtained from 10^−3^ M acetonitrile solutions, containing 0.1 M [*N*(^*n*^Bu)_4_]PF_6_ as supporting electrolyte at 20 °C. Potentials are relative to Ag/AgCl.

**Fig. 5:**
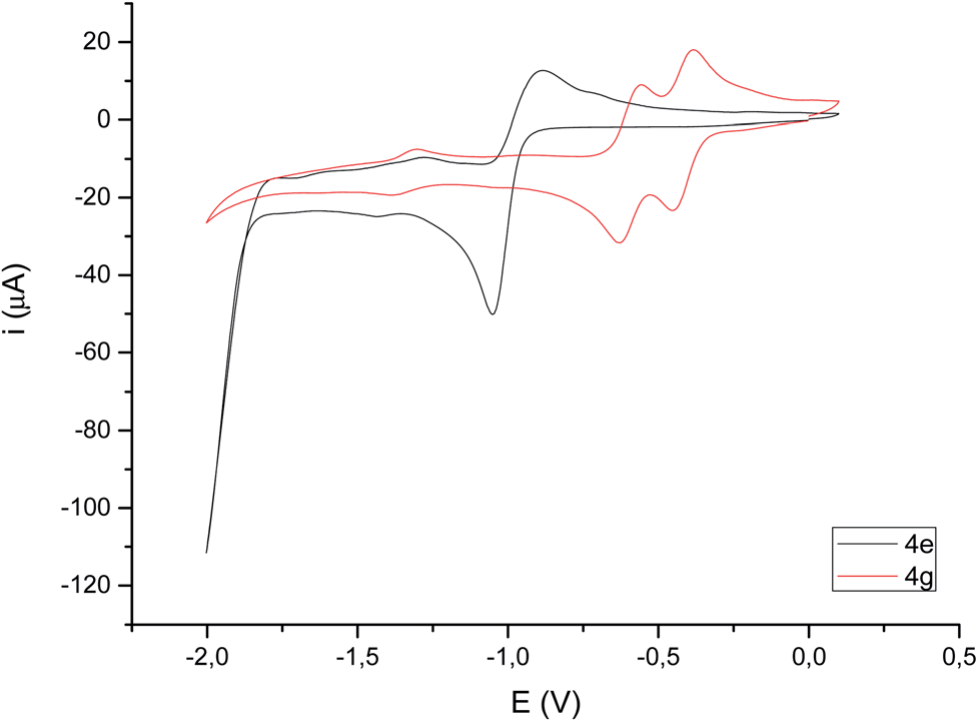
Cyclic voltammograms (focused on reduction) of compounds 4e and 4g (10^−3^ M in CH_3_CN), 10^−1^ M [*N*(^*n*^Bu)_4_]PF_6_, counter-electrode: Pt; working electrode: glassy carbon; reference electrode: Ag/AgCl; scan rate: 100 mV s^−1^; values given in V.

**Fig. 6:**
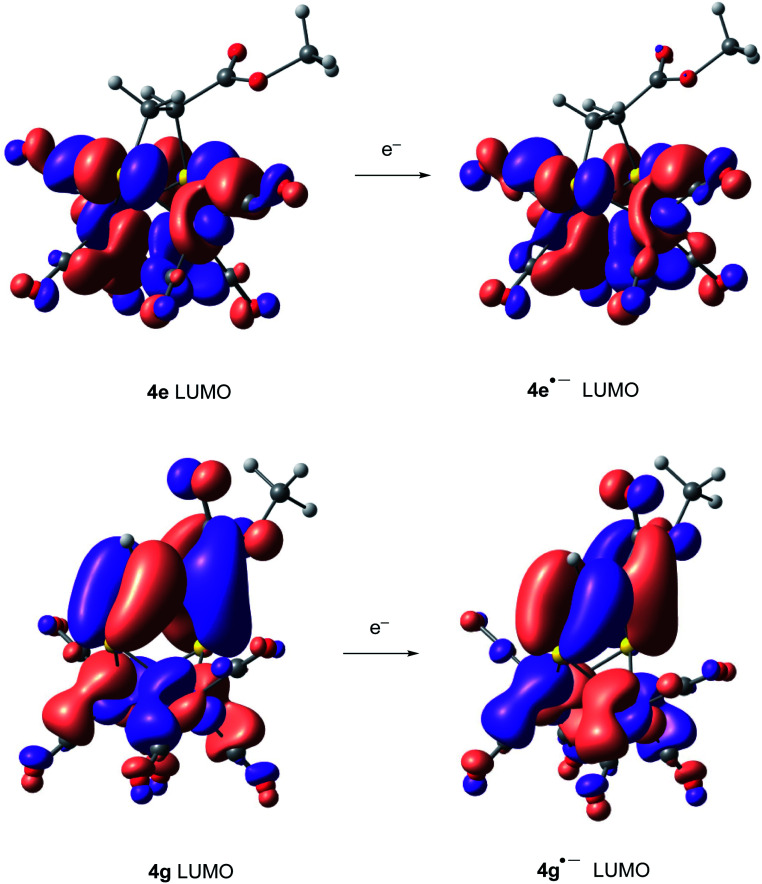
LUMO's of complexes 4e, 4e˙^−^, 4g and 4g˙^−^.

Compounds derived from alkenes behave like the analogous derivatives having [(μ-SR)_2_Fe_2_(CO)_6_] structures.^21^ However, compounds derived from alkynes show a strongly displaced anodic wave (even lower than those derivatives of type II in Fig. 1), together with the new reversible wave (see Fig. 5 for comparison). A similar behaviour has been reported for complex 4h.^22^ DFT calculations (BP86/Def2tzvpp/SCRF, CPCM-MeCN) were performed for further understanding this anodic displacement and the electrochemistry of these complexes. The LUMO in complex 4e is clearly centered in the [FeFe] moiety, while the LUMO of complex 4g having a double bond has a strong component in the organic moiety of the metallacycle (Fig. 6). Therefore, the strong anodic displacement caused by the presence of one double bond in the metallacycle may be explained by the reception of the electron by the organic moiety. Contrary to the complexes having one double bond those complexes having a saturated moiety receipt the electron into the metallic moiety, which accounts for a reduction potential in the −1.44 to −1.61 range. This situation is maintained in the radical-anions 4e˙^−^, and 4g˙^−^. The LUMO orbital of 4e˙^−^ is still localized across the [FeFe] fragment while radical anion 4g˙^−^ has the LUMO located in the organic moiety, which is easily reducible (still with strong anodic displacements, giving lower reduction potentials than their saturated congeners, due to the presence of the metals).^23^ This proposal nicely explain the “strong effect of the dithiolene and (in their case) tetrachloro-biphenyl dithiolate groups on the level of the LUMO” reported by Gloaguen and Schollhammer.^22^

**Fig. 7:**
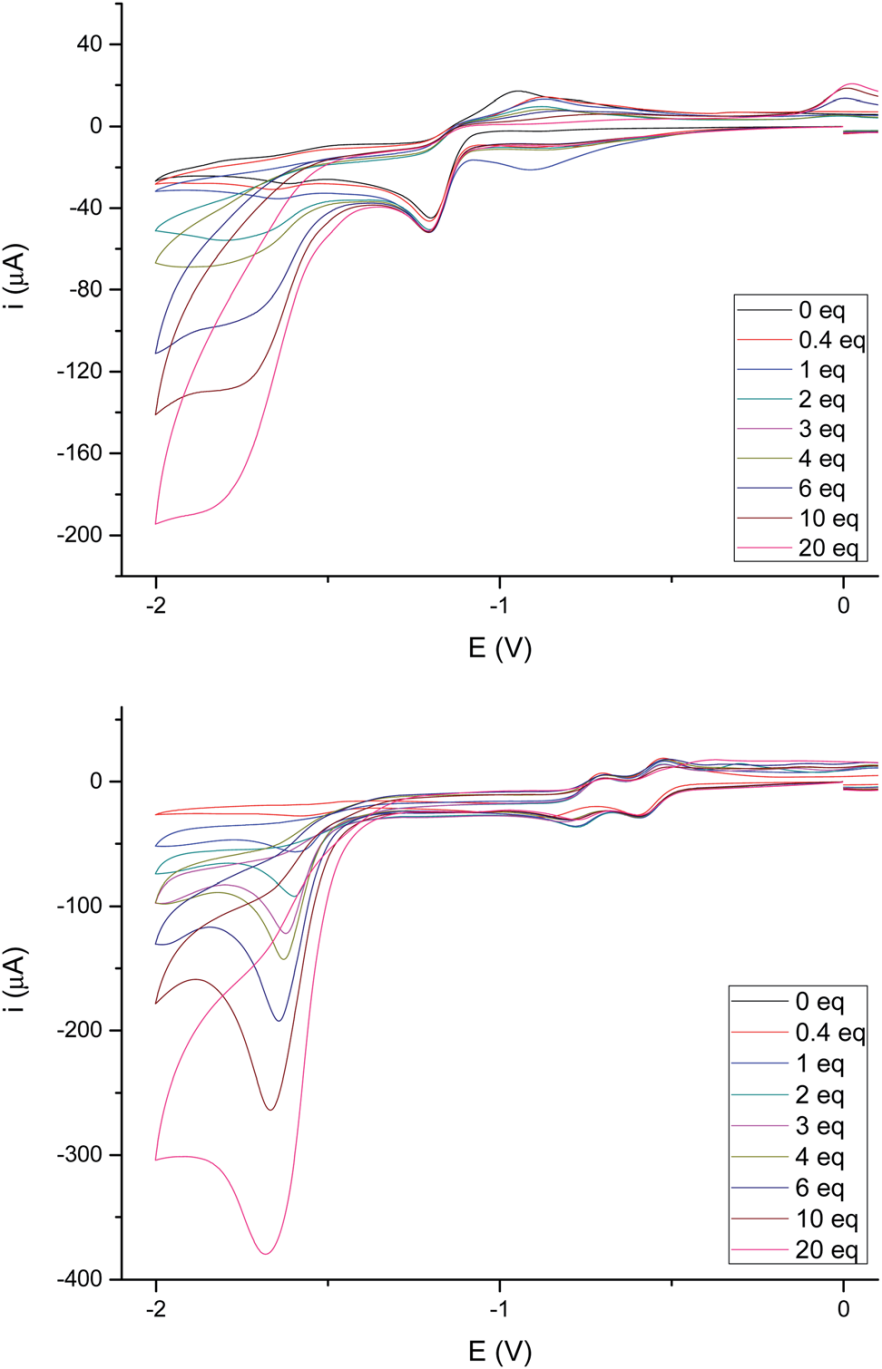
(Top) Cyclic voltammograms of 4e with added HOAc (0–20 eq.). Data (V) obtained from 10^−3^ M acetonitrile solutions, containing 0.1 M [N(^*n*^Bu)_4_]PF_6_ as supporting electrolyte at 20 °C. Potentials are relative to Ag/AgCl. (Bottom) Cyclic voltammograms of 4g with added HOAc (0–20 eq.). Data (V) obtained from 10^−3^ M acetonitrile solutions, containing 0.1 M [N(^*n*^Bu)_4_]PF_6_ as supporting electrolyte at 20 °C. Potentials are relative to Ag/AgCl.

The electrochemical behavior in acidic media of complexes 4e and 4g (as representative examples of complexes having either a saturated or double bond in the bridge joining the sulfur atoms) was next studied. None of these complexes showed electrocatalytic behavior in their first reduction wave in the presence of increasing amounts of acetic acid (p*K*_a_ ∼ 22.3 in MeCN)^24^ (up to 20 eq., see Fig. 7), while a peak appears around −1.80 V which increases its intensity with the concentration of acid. These results are fully consistent with those reported in the literature for related compounds.^25^ It should be noted that the first reduction wave at −0.42 V for compound 4g (the one attributed to the reduction of the double bond) remains quasi-reversible, while the second reduction wave at −0.60 V losses its quasi-reversibility in the presence of AcOH as previously reported.^22^

**Fig. 8:**
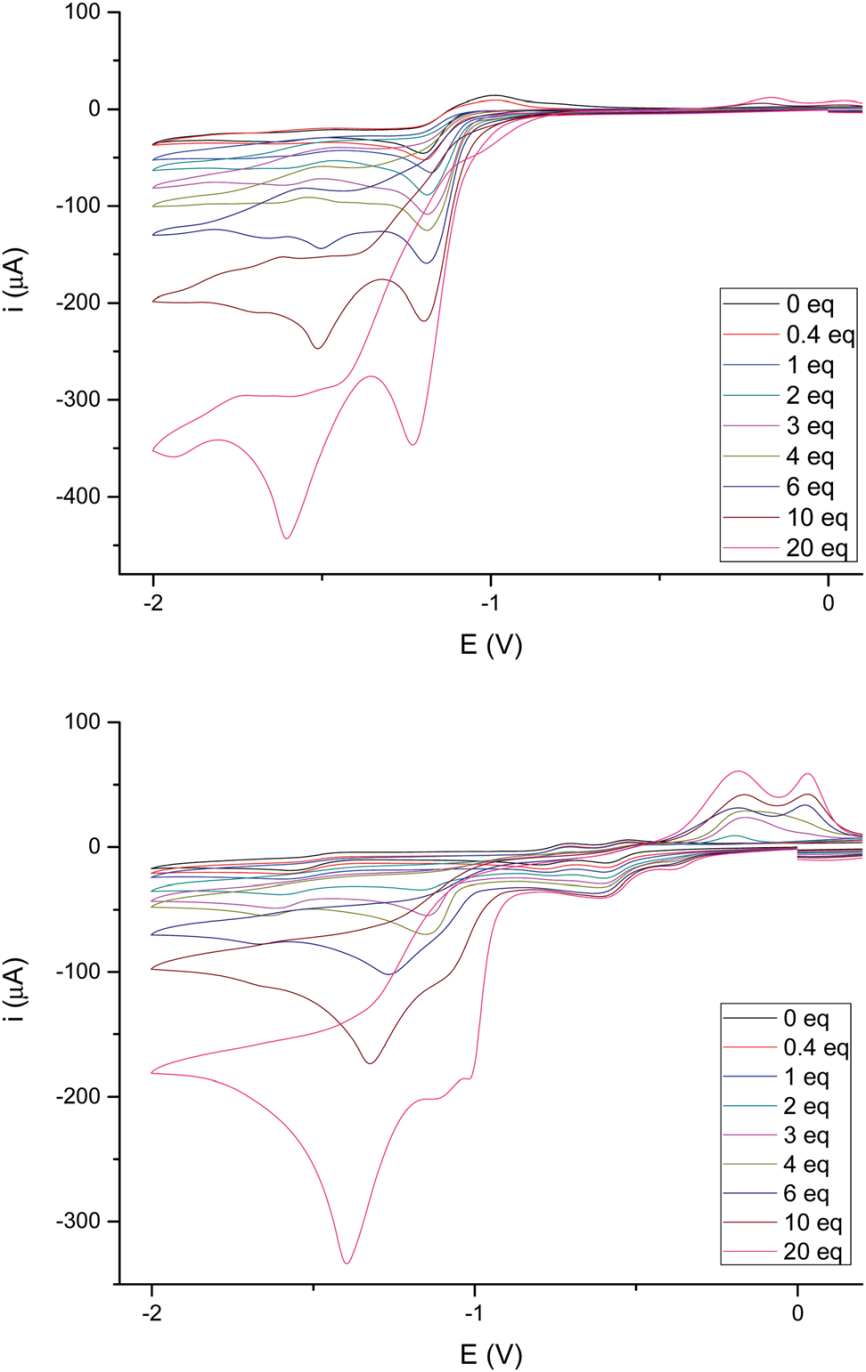
(Top) Cyclic voltammograms of 4e with added TFA (0–20 eq. of H^+^). Data (V) obtained from 10^−3^ M acetonitrile solutions, containing 0.1 M [*N*(^*n*^Bu)_4_]PF_6_ as supporting electrolyte at 20 °C. Potentials are relative to Ag/AgCl. (Bottom) Cyclic voltammograms of 4g with added TFA (0–20 eq. of H^+^). Data (V) obtained from 10^−3^ M acetonitrile solutions, containing 0.1 M [*N*(^*n*^Bu)_4_]PF_6_ as supporting electrolyte at 20 °C. Potentials are relative to Ag/AgCl.

The behavior of complexes 4e and 4g towards a stronger acid (CF_3_COOH, p*K*_a_ ∼ 12.6 in MeCN)^24^ was next studied. Fig. 8 shows the behavior of these complexes upon increasing additions of CF_3_COOH. The reduction of 4e (Fig. 8, top) becomes irreversible upon addition of less than 1 eq. of CF_3_COOH, as reported in the literature for related compounds.^26^ However, the intensity of the reduction wave at −0.97 V steadily increases with the concentration of acid and slightly shifts towards more negative values (up to 20 eq. of added CF_3_COOH). Therefore, the species generated in the electrochemical reduction are able to catalyze the proton reduction.^26*a*^ In addition, reduction of protons is also observed around −1.60 V, even at low acid concentrations (<0.5 eq.).

Complex 4g (Fig. 8, bottom) behaves differently. In this case, both waves (−0.42 V and −0.60 V) increase their intensity with the acid concentration until the ratio 4g/acid exceeds 4 equivalents. This supports the participation of the double bond in the first reduction event, generating species that are able to catalyze the reduction of protons.

## Conclusions

A smooth and efficient photochemical method to prepare functionalized [FeFe]-hydrogenase mimics has been developed. Irradiation of [(μ-S)_2_Fe_2_(CO)_6_] and alkene/alkynes under medium-low CO pressures produce the corresponding photoadducts in good (alkenes) or acceptable yields (alkynes). The formation of photoadducts derived from alkenes occurs with retention of the stereochemistry of the starting olefin, as demonstrated by NOE measurements and X-ray diffraction. The process is compatible with substrates having ferrocene moieties, as well as functional groups like imides and esters. The photocycloaddition occurs through a concerted reaction pathway as demonstrated by extensive DFT-calculations. The stereochemistry of these reactions is compatible with the computed pathway. Alternative reaction pathways involving triplet states are considerable higher in energy, and, for alkynes, predict the formation of products that have not been detected experimentally.

Photoadducts formed from alkynes present a double bond within the metallacycle, that strongly affect the electrochemistry of these compounds. Thus, in the presence of this double bond two strongly anodic displaced quasi-reversible reduction waves appear. These reduction events are compatible with the one electron^17^ reduction of the double bond “conjugated” with the [FeFe]-moiety. This one electron reduction, forms a radical-anion with a formal [Fe^0^Fe^I^] state, which facilitate the second reduction to form the [Fe^0^Fe^0^] state.

The electrochemistry of complexes 4 in the presence of acids reveals a different behaviour between complexes having a double bond in the dithiametallacycle and those lacking this insaturation. Thus, complexes lacking the insaturation in the metallacycle behave like the analogous products reported in the literature. For this compounds, in the presence of soft acids (AcOH) the species derived from the quasi-reversible reduction wave around −0.97 V are electrocatalytically inactive, and a new electrocatalytically active band appears at −1.80 V. Complexes 4 having a double bond in the metallacycle behave similarly towards soft acids. However, in the presence of strong acids (CF_3_COOH) the species formed upon reduction in the wave around −0.97 V are able to reduce protons. For these unsaturated complexes, a new reduction wave appears around −1.60 V that is also catalytically active. Therefore, for complexes having a double bond, both waves (−0.42 V and −0.60 V) become catalytically active, showing the participation of dithiolene ligand in its structure.

Further work to apply these smooth methodologies to prepare more sophisticated [FeFe]-mimics, together with post functionalization of the photoadducts, is now underway in our laboratories.

## Experimental section

### General

Flame-dried glassware was used for moisture-sensitive reactions, and anhydrous solvents were taken from a Pure Solvent PS-MD-5 apparatus. Silica gel (Merck: 230–400 mesh) was used as stationary phase for purification of crude reaction mixtures by flash column chromatography. NMR spectra were recorded at 25 °C in DMSO-*d*_6_ or CDCl_3_ on a 300 and 500 MHz spectrometers. IR spectra were taken on a MIR (8000–400 cm^−1^) spectrometer using the attenuated total reflectance (ATR) technique. HRMS experiments were recorded on an Agilent 6500 accurate mass apparatus with a Q-TOF analyzer. Cyclic voltammograms were recorded using a Metrohm Autolab Potentiostat model PGSTAT302N with a glassy carbon working electrode, Ag/AgCl 3 M as reference and a Pt wire counter electrode. All the measurements were performed under Ar, at room temperature from CH_3_CN solutions containing 0.1 M [N^*n*^Bu_4_]PF_6_ as supporting electrolyte, with analyte concentrations of 1 mM (scan rate 0.1 V s^−1^). When needed, an ultrasound bath was used to promote solubilization in those samples were a suspension was initially obtained.

### Computational details

Theoretical calculations have been performed using the Gaussian 09-D.01 software package^27^ at the BP86/Def2tzvpp^28^ level for all atoms. A SCRF, CPCM^29^ solvent model for THF was also used. Compounds 4e, 4g and their corresponding radical anions were also calculated using MeCN as solvent in order to match the conditions used in the electrochemical experiments An ultrafine-grid was used as integration grid for all the calculations as implemented in the G09 software suite.

### General procedure for the synthesis of [FeFe]-hydrogenase mimics

#### Photochemical reactions

Photochemical reactions were conducted by using a 125 W or 400 W-medium pressure mercury lamp through a pyrex filter/pyrex well. Starting materials were dissolved in dry and degassed (vacuum-Ar, four cycles) THF in a rubber septum-sealed Pyrex tube purged with argon. In a typical experiment, an equimolecular solution of [(μ-S)_2_Fe_2_(CO)_6_] 2 and the corresponding alkene or alkyne in dry THF (200 mL mmol^−1^) was bubbled with CO for 5 minutes and was irradiated overnight under CO pressure (1 atm, balloon). The solvent was then removed under reduced pressure, and the product was purified by SiO_2_ column chromatography.

#### Synthesis of 4c

Following the general procedure, a solution of bimetallic complex 2 (200 mg, 0.58 mmol) and 1-hexene 3c (49 mg, 0.58 mmol) in 100 mL of THF was irradiated (125 W) for 15 h. Purification by SiO_2_ chromatography (Hex/EtOAc 8 : 2) yielded pure 4c (159 mg, 64%) as a dark red solid. ^1^H NMR (300 MHz, CDCl_3_) *δ* 0,90 (t, 3H, *J* = 6.9 Hz, CH_3_), 1.25–1.57 (m, 6H, 3× CH_2_), 1.80 (dd, 1H, *J* = 12.3, 4.8 Hz, CH), 2.57–2.73 (m, 2H, CH_2_). ^13^C NMR (75 MHz, CDCl_3_) *δ* 208.7, 54.6, 42.2, 36.9, 31.8, 22.6, 14.0. IR (film): *ν* 2962, 2931, 2863, 2074, 2029, 1976 cm^−1^. Anal. calcd for C_12_H_12_Fe_2_O_6_S_2_: C, 33.67; H, 2.83; S, 14.98. Found C, 33.59; H, 2.91; S, 15.21.

#### Synthesis of 4d

Following the general procedure, a solution of bimetallic complex 2 (200 mg, 0.58 mmol) and *N*-phenylmaleimide 3d (101 mg, 0.58 mmol) in 100 mL of THF was irradiated (125 W) for 15 h. Purification by SiO_2_ chromatography (Hex/EtOAc 7 : 3) yielded pure 4d (211 mg, 70%) as an orange solid. ^1^H NMR (300 MHz, CDCl_3_) *δ* 3.92 (s, 2H, 2× CH), 7.25–7.34 (m, 2H, Ar), 7.38–7.51 (m, 3H, Ar). ^13^C NMR (75 MHz, CDCl_3_) *δ* 206.5, 169.9, 130.7, 129.1, 129.0, 125.7, 54.3. IR (film): *ν* 2925, 2854, 2083, 2043, 1998, 1717, 1374, 1184 cm^−1^. ESI-HRMS *m/z* calcd for C_16_H_7_Fe_2_NNaO_8_S_2_ [M + Na]^+^ 539.82047; found 539.82269.

#### Synthesis of 4e

Following the general procedure, a solution of bimetallic complex 2 (200 mg, 0.58 mmol) and methyl acrylate 3e (50 mg, 0.58 mmol) in 100 mL of THF was irradiated (125 W) for 15 h. Purification by SiO_2_ chromatography (Hex/EtOAc 8 : 2) yielded pure 4e (95 mg, 86%) as a red solid. ^1^H NMR (300 MHz, CDCl_3_) *δ* 2.57–2.53 (m, 2H, CH_2_), 3.28–3.32 (m, 1H, CH), 3.77 (s, 3H, CH_3_). ^13^C NMR (75 MHz, CDCl_3_) *δ* 207.8, 170.2, 53.3, 52.8, 38.8. IR (film): *ν* 2078, 2034, 1983, 1737 cm^−1^. Anal. calcd for C_10_H_6_Fe_2_O_8_S_2_: C, 27.94; H, 1.41; S, 14.91. Found C, 28.30; H, 1.77; S, 14.85.

#### Synthesis of 4f

Following the general procedure, a solution of bimetallic complex 2 (200 mg, 0.58 mmol) and 4-ethynyltoluene 3f (68 mg, 0.58 mmol) in 100 mL of THF was irradiated (125 W) for 15 h. Purification by SiO_2_ chromatography (Hex/EtOAc 8 : 2) yielded pure 4f (120 mg, 45%) as a red-orange solid. ^1^H NMR (300 MHz, CDCl_3_) *δ* 2.31 (s, 1H, CH_3_), 6.35 (s, 1H, CHS), 7.09 (d, 2H, *J* = 8.2 Hz, Ar), 7.21 (d, 2H, *J* = 8.2 Hz, Ar). ^13^C NMR (75 MHz, CDCl_3_) *δ* 207.7, 161.8, 140.1, 133.1, 130.7, 129.4, 125.2, 21.5. IR (film): *ν* 2074, 2029, 1976 cm^−1^. Anal. calcd for C_15_H_8_Fe_2_O_6_S_2_: C, 39.16; H, 1.75; S, 13.94. Found C, 39.04; H, 1.88; S, 13.75.

#### Synthesis of 4g

Following the general procedure, a solution of bimetallic complex 2 (200 mg, 0.58 mmol) and Methyl propiolate 3g (49 mg, 0.58 mmol) in 100 mL of THF was irradiated (125 W) for 15 h. Purification by SiO_2_ chromatography (Hex/EtOAc 9 : 1) yielded pure 4g (95 mg, 38%) as a red solid. ^1^H NMR (300 MHz, CDCl_3_) *δ* 3.68 (s, 3H, CH_3_), 7.37 (s, 1H, CH). ^13^C NMR (75 MHz, CDCl_3_) *δ* 206.2, 160.8, 157.6, 153.1, 52.2. IR (film): *ν* 2080, 2041, 1994, 1719, 1253 cm^−1^. ESI-HRMS *m/z* calcd for C_10_H_4_Fe_2_NaO_8_S_2_ [M + Na]^+^ 450.79390; found 450.79282.

#### Synthesis of 4h

Following the general procedure, a solution of bimetallic complex 2 (200 mg, 0.58 mmol) and dimethyl acetylenedicarboxylate 3h (83 mg, 0.58 mmol) in 100 mL of THF was irradiated (125 W) for 15 h. Purification by SiO_2_ chromatography (Hex/EtOAc 8 : 2) yielded pure 4h (176 mg, 62%) as a red solid. ^1^H NMR (300 MHz, CDCl_3_) *δ* 3.72 (s, 6H, 2× CH_3_). ^13^C NMR (75 MHz, CDCl_3_) *δ* 206.9, 162.3, 155.5, 53.3. IR (film): *ν* 2086, 2050, 2008, 1728, 1255 cm^−1^. ESI-HRMS *m/z* calcd for C_12_H_7_Fe_2_O_10_S_2_ [M + H]^+^ 486.81744; found 486.81522.

#### Synthesis of 4i

Following the general procedure, a solution of bimetallic complex 2 (200 mg, 0.58 mmol) and methyl *trans*-2-ferrocenylacrylate 3i (157 mg, 0.58 mmol) in 100 mL of THF was irradiated (125 W) for 15 h. Purification by SiO_2_ chromatography (Hex/EtOAc 8 : 2) yielded pure 4i (155 mg, 43%) as a red solid. ^1^H NMR (300 MHz, CDCl_3_) *δ* 3.16 (d, 1H, *J* = 6.3 Hz, CHCO), 3.86 (s, 3H, OCH_3_), 3.98 (bs, 1H, Cp), 4.08 (s, 5H, Cp), 4.13 (d, 1H, *J* = 6.3 Hz, CH–Cp), 4.21–4.28 (m, 3H, Cp). ^13^C NMR (300 MHz, CDCl_3_) *δ* 208.0, 170.9, 84.9, 69.5, 69.1, 68.9, 68.7, 65.4, 56.9, 56.0, 53.40. IR (film): *ν* 2075, 2033, 1980, 1735, 1266 cm^−1^. ESI-HRMS *m*/*z* calcd for C_20_H_15_Fe_3_O_8_S_2_ [M + H]^+^ 614.82522; found 614.82740.

#### Synthesis of 4j

Following the general procedure, a solution of bimetallic complex 2 (113 mg, 0.33 mmol) and ethynylpurine derivative 3j (133 mg, 0.33 mmol) in 100 mL of THF was irradiated (125 W) for 15 h. Purification by SiO_2_ chromatography (Hex/EtOAc 4 : 6) yielded pure 4j (59 mg, 24%) as a red solid. ^1^H NMR (500 MHz, CDCl_3_) *δ* 2.07 (s, 3H, CH_3_), 2.10 (s, 3H, CH_3_), 2.15 (s, 3H, CH_3_), 4.34–4.46 (m, 3H, 3× CH–O), 5.63 (t, 1H, *J* = 5.2 Hz, CH), 5.92 (t, 1H, *J* = 5.2 Hz, CH), 6.20 (d, 1H, *J* = 5.2 Hz, CH), 8.15 (s, 1H, Ar), 8.46 (s, 1H, =CH), 8.97 (s, 1H, Ar). ^13^C NMR (125 MHz, CDCl_3_) *δ* 207.4, 170.4, 169.7, 169.5, 157.5, 154,7, 152.6, 150.9, 148.9, 143.3, 130.2, 86.7, 80.6, 73.2, 70.6, 63.0, 20.9, 20.7, 20.5. IR (film): *ν* 2079, 2042, 2000, 1751, 1577, 1224 cm^−1^. ESI-HRMS *m/z* calcd for C_24_H_19_Fe_2_N_4_O_13_S_2_ [M + H]^+^ 746.90844; found 746.91145.

#### Synthesis of 4k

Following the general procedure, a solution of bimetallic complex 2 (200 mg, 0.58 mmol) and *N*,*N*′-(1,4-phenylene)dimaleimide 3k (117 mg, 0.58 mmol) in 100 mL of THF was irradiated (125 W) for 15 h. Purification by SiO_2_ chromatography (Hex/EtOAc 8 : 2) yielded pure 4k (107 mg, 26%) as a reddish solid. ^1^H NMR (300 MHz, DMSO-*d*_6_) *δ* 4.48 (s, 4H, CH), 7.39 (s, 4H, Ar), ^13^C NMR (75 MHz, CDCl_3_) *δ* 182.0, 170.4, 130.6, 126.4, 54.2. IR (film): *ν* 2080, 2039, 2008, 1979, 1781, 1708 cm^−1^. ESI-HRMS *m/z* calcd for C_26_H_12_Fe_4_N_3_O_16_S_4_ [M + NH_4_]^+^ 973.64953; found 973.64842.

#### Synthesis of 4l and 4m

Following the general procedure, a solution of bimetallic complex 2 (319 mg, 0.93 mmol) and (*E*,*E*)-1,1′-bis-[β-(methoxycarbonyl)ethenyl]ferrocene 3l (150 mg, 0.42 mmol) in 100 mL of THF was irradiated (125 W) for 15 h. Purification by SiO_2_ chromatography (Hex/EtOAc 7 : 3) yielded 153 mg (35%) of pure complex 4l. Pure complex 4m (154 mg, 52%) was also isolated.

##### Complex 4l (35%)

^1^H NMR (300 MHz, CDCl_3_) *δ* 3.04 (d, 1H, *J* = 6.1 Hz, CHCO), 3.77 (s, 3H, OCH_3_), 3.87 (s, 3H, OCH_3_), 3.95 (s, 1H, Cp), 4.03 (d, 1H, *J* = 6.1 Hz, CHS), 4.14–4.49 (m, 7H, Cp), 6.03 (d, 1H, *J* = 15.8 Hz, CH), 7.49 (d, 1H, *J* = 15.8 Hz, CH). ^13^C NMR (300 MHz, CDCl_3_) *δ* 207.9, 170.6, 167.4, 144.6, 115.9, 86.5, 79.8, 72.0, 71.9, 71.4, 70.7, 69.7, 69.4, 67.0, 56.5, 55.9, 53.5, 51.6. IR (film): *ν* 2075, 2034, 1976, 1723(sh), 1701, 1629, 1435, 1264, 1195, 1157 cm^−1^. ESI-HRMS *m*/*z* calcd for C_24_H_19_Fe_3_O_10_S_2_ [M + H]^+^ 698.84638; found 698.84799.

##### Complex 4m (52%)

^1^H NMR (300 MHz, CDCl_3_) *δ* 3.09 (d, 1H, *J* = 6 Hz, CHCO), 3.11 (d, 1H, *J* = 6 Hz, CHCO), 3.86 (s, 3H, OCH_3_), 3.87–3.91 (m, 2H, Cp), 3.89 (s, 3H, OCH_3_), 4.03–4.08 (m, 3H, CHS, Cp), 4.12–4.20 (m, 5H, Cp). ^13^C NMR (300 MHz, CDCl_3_) *δ* 208.5, 170.8, 170.8, 86.1, 86.0, 70.7, 70.4, 70.1, 70.1, 69.7, 69.5, 66.5, 65.6, 56.2, 56.1, 55.8, 53.6, 53.5. IR (film): *ν* 2075, 2034, 1976, 1701, 1629, 1434, 1306, 1263, 1194 cm^−1^. ESI-HRMS *m*/*z* calcd for C_30_H_18_Fe_5_NaO_16_S_4_ [M + Na]^+^ 1064.61211; found 1064.60748.

#### Synthesis of 4n

Following the general procedure, a solution of bimetallic complex 2 (331 mg, 0.97 mmol) and 1,3-bis(2-propynyloxy)benzene 3n (80 mg, 0.44 mmol) in 100 mL of THF was irradiated (400 W) for 15 h. Purification by SiO_2_ chromatography (Hex/EtOAc 9 : 1) yielded pure 4n (40 mg, 17%) as a red solid. ^1^H NMR (300 MHz, CDCl_3_) *δ* 2.53 (t, 1H, *J* = 2.4 Hz, ≡CH), 4.21 (d, 2H, *J* = 1.9 Hz, OCH_2_), 4.66 (d, 2H, *J* = 2.4 Hz, ≡C–CH_2_O), 6.23 (t, 1H, *J* = 1.9 Hz, =CH), 6.37–6.41 (m, 2H, Ar), 6.59–6.62 (m, 1H, Ar), 7.18 (dd, 1H, *J* = 9.2, 7.4 Hz, Ar). ^13^C NMR (75 MHz, CDCl_3_) *δ* 207.5, 158.9, 158.7, 158.6, 158.3, 138.8, 130.2, 108.1, 107.6, 102.5, 75.8, 66.6, 56.0. IR (film): *ν* 2074, 2030, 1970, 1886, 1502, 1208 cm^−1^. Anal. calcd for C_18_H_10_Fe_2_O_8_S_2_: C, 40.79; H, 1.90; S, 12.10. Found, C, 40.43; H, 2.14; S, 12.54.

#### Synthesis of complexes 4o and 4p

Following the general procedure, a solution of bimetallic complex 2 (198 mg, 0.58 mmol) and 1,4-bis(prop-2-yn-1-yloxy)benzene 3o (50 mg, 0.26 mmol) in 100 mL of THF was irradiated (125 W) for 15 h. Purification by SiO_2_ chromatography (Hex/EtOAc 20 : 1) yielded 77 mg (55%) of pure 4o and 46 mg (20%) of pure 4p as red solids.

##### Complex 4o (55%)

^1^H NMR (300 MHz, CDCl_3_) *δ* 2.10 (dd, 1H, *J* = 13.2, 5.5 Hz, C*H*_2_S), 2.75 (dd, 1H, *J* = 13.2, 7.4 Hz, CH̲_2_S), 2.98–3.06 (m, 1H, CHS), 3.79 (dd, 1H, *J* = 9.7, 7.0 Hz, SCHCH̲_2_), 3.97 (dd, 1H, *J* = 9.7, 5.8 Hz, SCHCH̲_2_), 4.49 (bd, 2H, *J* = 5.4 Hz, CH̲_2_CH=), 5.27 (d, 1H, *J* = 10.5 Hz, =CH̲_2_), 5.39 (d, 1H, *J* = 16.1 Hz, =CH̲_2_), 6.04 (ddt, 1H, *J* = 16.1, 10.5, 5.4 Hz, CH=), 6.74–6.91 (m, 4H, Ar). ^13^C NMR (75 MHz, CDCl_3_) *δ* 208.4, 153.5, 152.3, 133.50, 117.8, 115.9, 115.7, 70.8, 69.6, 52.7, 39.1. IR (film): *ν* 2074, 2029, 1969, 1885, 1502, 1208 cm^−1^. Anal. calcd for C_18_H_14_Fe_2_O_8_S_2_: C, 40.48; H, 2.64; S, 12.00. Found, C, 40.31; H, 2.74; S, 12.20.

##### Complex 4p (20%)

^1^H NMR (300 MHz, CDCl_3_) *δ* 2.10 (dd, 2H, *J* = 13.1, 5.6 Hz, CH̲_2_S), 2.75 (dd, 2H, *J* = 13.1, 7.5 Hz, CH̲_2_S), 2.97–3.06 (m, 2H, CHS), 3.79 (dd, 2H, *J* = 9.7, 7.0 Hz, OCH̲_2_), 3.96 (dd, 2H, *J* = 9.7, 5.6 Hz, OC*H*_2_), 6.81 (s, 4H, Ar). ^13^C NMR (75 MHz, CDCl_3_) *δ* 208.3, 152.9, 115.8, 70.7, 52.6, 39.1. IR (film): *ν* 2075, 2032, 1985, 1506, 1223 cm^−1^. Anal. calcd for C_24_H_14_Fe_4_O_14_S_4_: C, 32.83; H, 1.61; S, 14.61. Found, C, 33.11; H, 1.97; S, 15.10.

### Crystal data for compound 4d

C_16_H_7_Fe_2_NO_8_S_2_, *M* = 517.05, monoclinic, *a* = 13.23734(18), *b* = 7.41493(10), *c* = 18.8284(2) Å, *β* = 92.6332(12)°, *V* = 1846.13 Å^3^, space group *P*_2(1)/c_, *Z* = 4, *T* = 120(2) K, *λ* = 0.71073 Å, *D*_calcd_ = 1.860 g cm^−3^, *μ* = 1.844 cm^−1^, 42 161 reflections measured, 6112 unique (*R*_int_ = 0.0427), dark red tablets obtained by CH_2_Cl_2_/*n*-hexane diffusion, crystal structure solved by dual-space methods with all non-hydrogen atoms refined anisotropically on *F*^2^ using the programs SHELXT^30^ and SHELXL-2018,^31^ hydrogen atoms were included using a *riding* model, GOF = 1.028, *R* (Fo, *I* > 2*σ*(*I*)) = 0.0270, *R*_w_ (Fo^2^, all data) = 0.0610.

## Conflicts of interest

The authors declare no competing financial interest.

## Supplementary Material

RA-010-D0RA06002J-s001

RA-010-D0RA06002J-s002
