# Supplementary material for: Revisiting the photochemical synthesis of [FeFe]-hydrogenase mimics: reaction optimization, mechanistic study and electrochemical behaviour
Source: RSC Adv. 2020 Aug 12;10(50):29855–67. doi: 10.1039/d0ra06002j (PMC9056276; doi:10.1039/d0ra06002j)
Supplement: RA-010-D0RA06002J-s001 [file RA-010-D0RA06002J-s001.pdf]

Electronic Supplementary Material (ESI) for RSC Advances.
This journal is © The Royal Society of Chemistry 2020

Supporting Information Belonging to the Manuscript:

Revisiting the Photochemical Synthesis of [FeFe]- Hydrogenase Mimics: Reaction Optimization, Mechanistic Study and Electrochemical Behaviour

Sergio Aguado,[†] Luis Casarrubios,[†] Miguel A. Sierra,[†]* Carmen Ramírez de Arellano[≠]*

[†]Departamento de Química Orgánica I, Facultad de Química, and Center for Innovation in Advanced Chemistry (ORFEO-CINQA), Universidad Complutense 28040-Madrid. Spain.

[≠]Departamento de Química Orgánica and Center for Innovation in Advanced Chemistry (ORFEO-CINQA), Universidad de Valencia 46100-Valencia. Spain.

SUPPORTING INFORMATION

Electrochemical study:

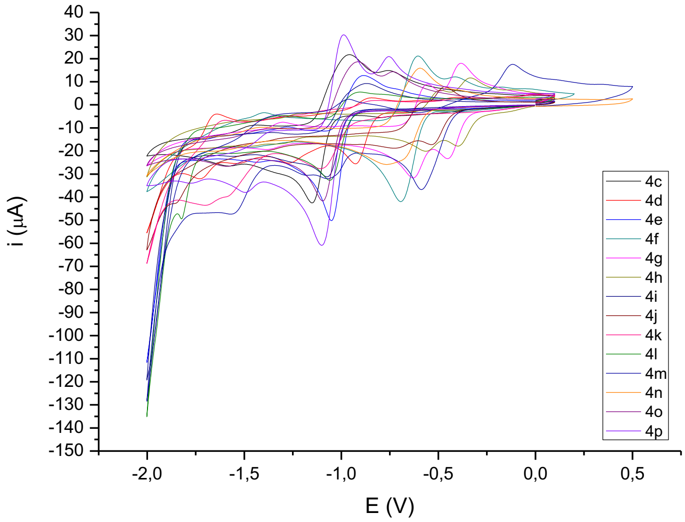

Figure 1. Cyclic voltammograms (focused on reduction) of selected compounds **4c-p** (10^{-3} M in CH_3CN), 10^{-1} M $[N(^nBu)_4]PF_6$, counter-electrode: Pt; working electrode: Glassy Carbon; Reference electrode: Ag/AgCl; scan rate: 100 mV/s; values given in V.

Electrochemical studies in the presence of acids.

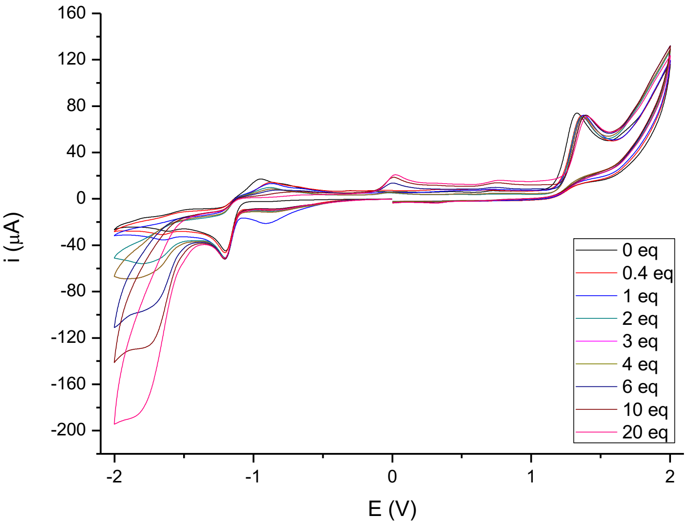

Figure 2. Cyclic voltammograms of **4e** with added HOAc (0-20 eq.). Data (V) obtained from 10-3M acetonitrile solutions, containing 0.1 M $[N(^nBu)_4]PF_6$ as supporting electrolyte at 20 ºC. Potentials are relative to Ag/AgCl.

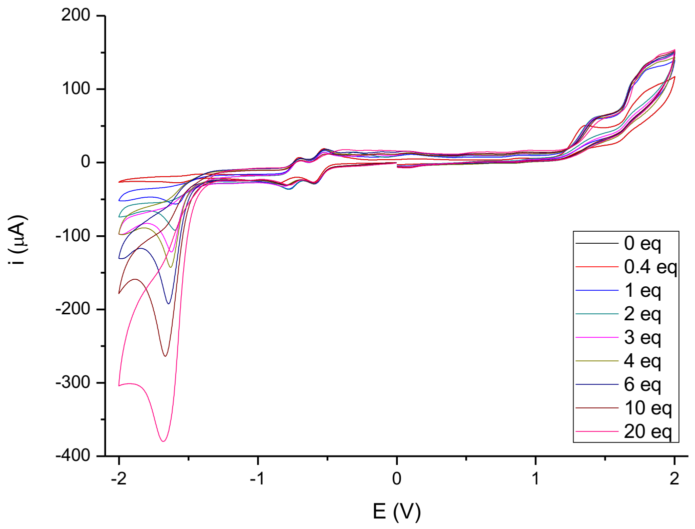

Figure 3. Cyclic voltammograms of **4g** with added HOAc (0-20 eq.). Data (V) obtained from 10-3M acetonitrile solutions, containing 0.1 M $[N(^nBu)_4]PF_6$ as supporting electrolyte at 20 ºC. Potentials are relative to Ag/AgCl.

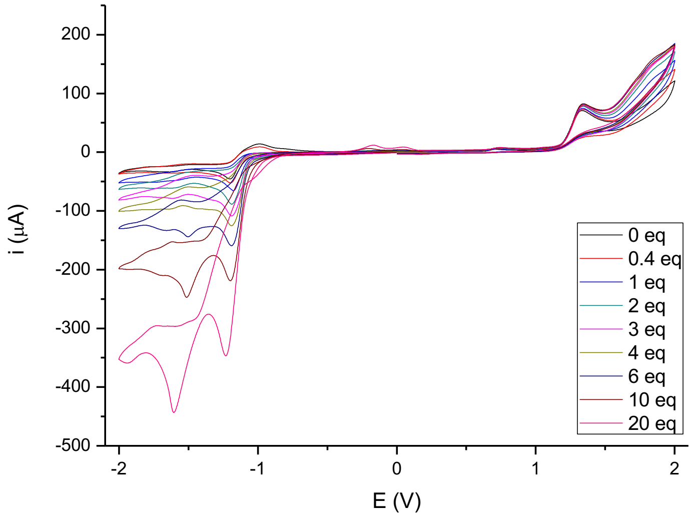

Figure 4. Cyclic voltammograms of **4e** with added TFA (0-20 eq.). Data (V) obtained from 10-3M acetonitrile solutions, containing 0.1 M $[N(^nBu)_4]PF_6$ as supporting electrolyte at 20 ºC. Potentials are relative to Ag/AgCl.

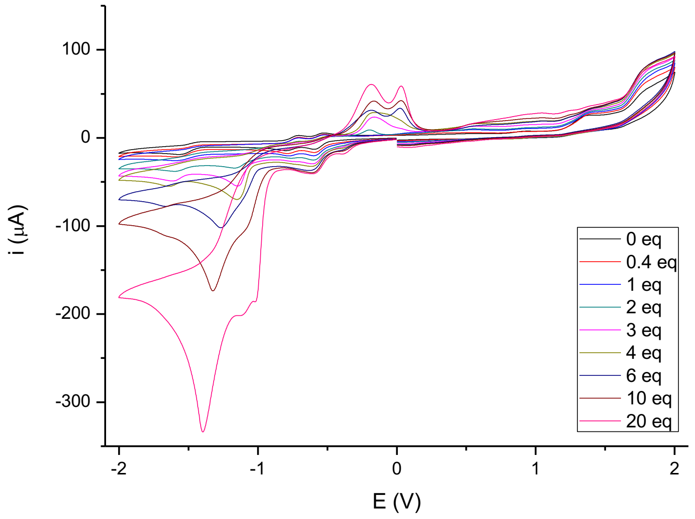

Figure 5. Cyclic voltammograms of **4g** with added TFA (0-20 eq.). Data (V) obtained from 10-3M acetonitrile solutions, containing 0.1 M $[N(^nBu)_4]PF_6$ as supporting electrolyte at 20 ºC. Potentials are relative to Ag/AgCl.

DFT Calculations:

Theoretical calculations have been performed using the Gaussian 09-D.01 software package[1] at the BP86/Def2tzvpp[2] level for all atoms. A SCRF, CPCM[3] solvent model for THF was also used. Compounds **3e**, **3g** and their corresponding radical anions were also calculated using MeCN as solvent in order to match the conditions used in the electrochemistry experiments. An ultrafine-grid was used as integration grid for all the calculations as implemented in the Gaussian 09 software suite. Transition states[4] were confirmed by the existence of a single imaginary frequency and by performing the corresponding IRC calculations in each case. Reactive complexes and products were

obtained by subsequent optimization of the two final geometries obtained in the IRC calculation.

Cartesian coordinates of the implied species.

1. Reaction of complex 1 with methyl propiolate 2g.

1a. Singlet state.

Reactive complex (1 + 2g)^s

Sum of electronic and zero-point Energies=	-4310.156267
Sum of electronic and thermal Energies=	-4310.128279
Sum of electronic and thermal Enthalpies=	-4310.127335
Sum of electronic and thermal Free Energies=	-4310.229299

26	1.783293000	-1.390398000	-0.000156000
26	1.813272000	1.382775000	0.059834000
16	1.488941000	0.036802000	-1.660818000
16	0.726035000	-0.021595000	1.372660000
8	-0.276144000	3.342552000	-0.426843000
8	2.858300000	2.556891000	2.570656000
8	3.906141000	2.638128000	-1.619173000
8	2.803609000	-2.684122000	2.461471000
8	-0.339261000	-3.290789000	-0.572428000
8	3.858984000	-2.605646000	-1.728558000
6	0.564261000	2.575730000	-0.230046000
6	2.451998000	2.096715000	1.595989000
6	3.093486000	2.146512000	-0.967424000
6	2.406672000	-2.178195000	1.505750000
6	0.514154000	-2.547850000	-0.342227000
6	3.052220000	-2.130009000	-1.057832000
6	-6.016836000	-0.159358000	-2.638286000
1	-5.650578000	-0.317273000	-3.634071000
6	-6.430518000	0.014961000	-1.512070000
6	-6.930622000	0.315998000	-0.187241000
8	-7.223170000	1.440701000	0.187607000
8	-7.025887000	-0.802677000	0.562563000
6	-7.519930000	-0.599671000	1.915040000
1	-8.530303000	-0.173676000	1.888171000
1	-7.529989000	-1.594895000	2.367258000
1	-6.849322000	0.071414000	2.465236000

TS1$^s_{3g}$

Sum of electronic and zero-point Energies=	-4310.152735
Sum of electronic and thermal Energies=	-4310.126251
Sum of electronic and thermal Enthalpies=	-4310.125307

Sum of electronic and thermal Free Energies= -4310.213566

26	-0.650264000	1.430022000	0.024369000
26	-1.088977000	-1.229216000	0.113402000
16	-0.921168000	0.051576000	-1.671565000
16	0.603882000	-0.113771000	0.964066000
8	0.156048000	-3.698989000	-0.811302000
8	-1.622914000	-2.125491000	2.883453000
8	-3.832025000	-1.845353000	-0.816273000
8	-0.903529000	2.583851000	2.737945000
8	1.320186000	3.324022000	-0.993367000
8	-3.054662000	2.828787000	-0.989580000
6	-0.344766000	-2.728263000	-0.438575000
6	-1.417115000	-1.774171000	1.805509000
6	-2.764861000	-1.604119000	-0.454711000
6	-0.804046000	2.135355000	1.681133000
6	0.534582000	2.583331000	-0.584772000
6	-2.118708000	2.283836000	-0.595900000
6	1.721138000	-0.404265000	-2.352512000
1	1.298609000	-0.366683000	-3.337265000
6	2.422252000	-0.484280000	-1.352101000
6	3.436678000	-0.711607000	-0.345600000
8	3.738982000	-1.817391000	0.079058000
8	4.022350000	0.448266000	0.029991000
6	5.072350000	0.314964000	1.025142000
1	5.886625000	-0.308913000	0.636838000
1	5.420913000	1.334558000	1.210320000
1	4.670176000	-0.131103000	1.942966000

Complex 3g (THF)

Sum of electronic and zero-point Energies= -4310.233299
Sum of electronic and thermal Energies= -4310.207945
Sum of electronic and thermal Enthalpies= -4310.207001
Sum of electronic and thermal Free Energies= -4310.291591

26	0.841043000	-1.254521000	0.014707000
26	0.841957000	1.254284000	0.014864000
16	0.309671000	0.000111000	-1.849489000
16	-0.893012000	0.000532000	0.846396000
8	-0.477172000	3.823663000	-0.559981000
8	1.787070000	1.890943000	2.721218000
8	3.494875000	1.897813000	-1.076376000
8	1.787849000	-1.889681000	2.720842000
8	-0.481481000	-3.822793000	-0.557235000
8	3.492023000	-1.902712000	-1.078452000
6	0.030095000	2.809094000	-0.340456000
6	1.415566000	1.631846000	1.657697000
6	2.453294000	1.636492000	-0.648876000
6	1.415694000	-1.631200000	1.657399000

6	0.027125000	-2.808607000	-0.339048000
6	2.451249000	-1.639488000	-0.650158000
6	-1.472835000	0.000812000	-1.768716000
1	-2.025008000	0.001126000	-2.709017000
6	-2.044269000	0.000914000	-0.559132000
6	-3.517249000	0.000977000	-0.362196000
8	-4.327069000	0.001707000	-1.276999000
8	-3.843761000	0.000168000	0.948746000
6	-5.266213000	-0.000310000	1.236519000
1	-5.737209000	0.896294000	0.815331000
1	-5.736707000	-0.896880000	0.814685000
1	-5.339028000	-0.000702000	2.327353000

Complex 3g (MeCN)

Sum of electronic and zero-point Energies= -4310.234450
Sum of electronic and thermal Energies= -4310.209093
Sum of electronic and thermal Enthalpies= -4310.208148
Sum of electronic and thermal Free Energies= -4310.292765

26	-0.841036000	1.254488000	0.014822000
26	-0.841488000	-1.254374000	0.015042000
16	-0.310572000	-0.000161000	-1.849515000
16	0.893533000	-0.000246000	0.845205000
8	0.477405000	-3.823601000	-0.560344000
8	-1.785250000	-1.891428000	2.721657000
8	-3.494569000	-1.898973000	-1.074775000
8	-1.786628000	1.889931000	2.721189000
8	0.480177000	3.823092000	-0.558008000
8	-3.492541000	1.902515000	-1.076769000
6	-0.029670000	-2.808862000	-0.340648000
6	-1.414196000	-1.632123000	1.657874000
6	-2.452771000	-1.637138000	-0.647781000
6	-1.414834000	1.631284000	1.657505000
6	-0.027837000	2.808569000	-0.339488000
6	-2.451402000	1.639275000	-0.649031000
6	1.471888000	-0.000483000	-1.770035000
1	2.021929000	-0.000599000	-2.711516000
6	2.044126000	-0.000560000	-0.560792000
6	3.516693000	-0.000889000	-0.363020000
8	4.327551000	-0.000782000	-1.277755000
8	3.842477000	-0.000006000	0.947372000
6	5.264736000	0.000382000	1.238323000
1	5.736062000	-0.896749000	0.818905000
1	5.735789000	0.897194000	0.817915000
1	5.335109000	0.001006000	2.329203000

Anion radical 3g$^{\bullet-}$ (MeCN)

Sum of electronic and zero-point Energies= -4310.377769

Sum of electronic and thermal Energies= -4310.351856
Sum of electronic and thermal Enthalpies= -4310.350912
Sum of electronic and thermal Free Energies= -4310.437520

26	0.859801000	-1.263759000	0.027380000
26	0.859189000	1.263958000	0.027369000
16	0.247521000	-0.000149000	-1.875669000
16	-0.932941000	-0.000321000	0.819640000
8	-0.442227000	3.828863000	-0.555143000
8	1.802724000	1.870562000	2.756062000
8	3.510174000	1.862138000	-1.112801000
8	1.804935000	-1.868184000	2.755992000
8	-0.441228000	-3.829331000	-0.553050000
8	3.510308000	-1.862184000	-1.113791000
6	0.065858000	2.806110000	-0.331482000
6	1.408699000	1.610267000	1.692705000
6	2.457132000	1.611260000	-0.685630000
6	1.410243000	-1.608824000	1.692656000
6	0.066736000	-2.806321000	-0.330296000
6	2.457475000	-1.611179000	-0.686187000
6	-1.482872000	-0.000532000	-1.793351000
1	-2.042441000	-0.000627000	-2.728413000
6	-2.067740000	-0.000669000	-0.549832000
6	-3.511844000	-0.000984000	-0.354075000
8	-4.346964000	-0.000637000	-1.262684000
8	-3.873416000	-0.000344000	0.968922000
6	-5.294993000	0.000089000	1.219060000
1	-5.765934000	0.895072000	0.791223000
1	-5.766338000	-0.895037000	0.791970000
1	-5.398924000	0.000569000	2.308738000

1b. Triplet state.

Reactive complex (1 + 2g)^t

Sum of electronic and zero-point Energies= -4310.141792
Sum of electronic and thermal Energies= -4310.113538
Sum of electronic and thermal Enthalpies= -4310.112594
Sum of electronic and thermal Free Energies= -4310.217990

26	-2.474875000	1.123693000	0.124399000
26	-1.284708000	-1.285990000	-0.136668000
16	-0.844218000	0.568394000	-1.284652000
16	-0.892459000	0.253091000	1.423962000
8	1.244268000	-2.762829000	-0.310307000
8	-2.550994000	-3.066299000	1.849247000
8	-2.550085000	-2.544196000	-2.488424000
8	-4.443968000	0.898418000	2.312394000
8	-2.134485000	4.031922000	0.316905000
8	-4.471338000	1.151331000	-2.050813000
6	0.246609000	-2.185730000	-0.242653000

6	-2.063885000	-2.375034000	1.064886000
6	-2.063178000	-2.058499000	-1.562407000
6	-3.684086000	0.984326000	1.448888000
6	-2.272660000	2.888050000	0.242330000
6	-3.699570000	1.141212000	-1.193639000
6	7.079012000	2.996555000	-0.623395000
1	6.915371000	4.039746000	-0.811866000
6	7.262471000	1.818210000	-0.405266000
6	7.513465000	0.402692000	-0.234683000
8	7.964033000	-0.316440000	-1.112744000
8	7.185118000	-0.003040000	1.010161000
6	7.403324000	-1.415589000	1.277131000
1	8.464444000	-1.663708000	1.153457000
1	7.088507000	-1.561738000	2.313681000
1	6.798251000	-2.026409000	0.596258000

TS1$^{t}_{3g}$

Sum of electronic and zero-point Energies= -4310.128461
Sum of electronic and thermal Energies= -4310.101600
Sum of electronic and thermal Enthalpies= -4310.100656
Sum of electronic and thermal Free Energies= -4310.192662

26	-1.791094000	0.790910000	0.121051000
26	-0.121652000	-1.243215000	-0.071457000
16	-0.040675000	0.646036000	-1.277115000
16	-0.152564000	0.289435000	1.526613000
8	2.628119000	-2.274246000	-0.033402000
8	-1.180192000	-3.261277000	1.796095000
8	-1.015687000	-2.705680000	-2.478590000
8	-3.798834000	-0.041834000	2.112688000
8	-2.191811000	3.676667000	0.499879000
8	-3.620787000	0.448723000	-2.173284000
6	1.551474000	-1.856050000	-0.049336000
6	-0.770173000	-2.473979000	1.058805000
6	-0.670644000	-2.135014000	-1.537313000
6	-3.017212000	0.280647000	1.327399000
6	-2.030485000	2.542484000	0.349993000
6	-2.908018000	0.578688000	-1.275317000
6	1.583983000	2.190670000	-0.763501000
1	0.946874000	3.051831000	-0.892692000
6	2.727376000	1.768932000	-0.518126000
6	3.985078000	1.100030000	-0.356404000
8	4.717773000	0.793172000	-1.290950000
8	4.263548000	0.880012000	0.952158000
6	5.537976000	0.233374000	1.209853000
1	6.360006000	0.851964000	0.828686000
1	5.595840000	0.139099000	2.297512000
1	5.567318000	-0.753491000	0.732027000

I1$^{t}_{3g}$

Sum of electronic and zero-point Energies= -4310.139008
Sum of electronic and thermal Energies= -4310.112165
Sum of electronic and thermal Enthalpies= -4310.111221
Sum of electronic and thermal Free Energies= -4310.202280

26	1.633897000	-0.893772000	0.095293000
26	0.163722000	1.280509000	0.001884000
16	0.140791000	-0.379705000	-1.567613000
16	-0.010870000	-0.367442000	1.472020000
8	-2.477958000	2.502972000	-0.409441000
8	0.828687000	3.015012000	2.284204000
8	1.668528000	3.005219000	-1.876948000
8	3.414430000	-0.770435000	2.433848000
8	1.620856000	-3.823830000	-0.090441000
8	3.862791000	-0.319644000	-1.769750000
6	-1.453324000	1.992696000	-0.252791000
6	0.575764000	2.332857000	1.388575000
6	1.082771000	2.323115000	-1.154237000
6	2.716893000	-0.809801000	1.515355000
6	1.626718000	-2.670823000	-0.021619000
6	2.985818000	-0.539699000	-1.053294000
6	-1.343013000	-1.395236000	-1.401309000
1	-1.233175000	-2.369950000	-1.893472000
6	-2.440793000	-1.003405000	-0.826705000
6	-3.803903000	-0.856161000	-0.441820000
8	-4.701414000	-0.567884000	-1.234670000
8	-3.983749000	-1.049022000	0.890948000
6	-5.349877000	-0.890721000	1.354049000
1	-6.000052000	-1.639772000	0.884579000
1	-5.304998000	-1.042362000	2.435902000
1	-5.718269000	0.115238000	1.116633000

TS2$^{t}_{3g}$

Sum of electronic and zero-point Energies= -4310.138193
Sum of electronic and thermal Energies= -4310.112269
Sum of electronic and thermal Enthalpies= -4310.111325
Sum of electronic and thermal Free Energies= -4310.199096

26	-1.557045000	0.893483000	0.052557000
26	-0.099515000	-1.275412000	0.062649000
16	-0.312560000	0.153803000	-1.733816000
16	0.261042000	0.569382000	1.267558000
8	2.159339000	-2.806183000	-1.066261000
8	0.018115000	-2.708816000	2.630011000
8	-2.257382000	-3.024180000	-0.953301000
8	-3.007359000	0.985377000	2.611921000
8	-1.511440000	3.781750000	-0.435934000

8	-4.040218000	0.308094000	-1.449943000
6	1.299850000	-2.174022000	-0.627541000
6	-0.037957000	-2.142496000	1.626574000
6	-1.418812000	-2.330202000	-0.569737000
6	-2.435693000	0.943373000	1.611129000
6	-1.537368000	2.643056000	-0.247463000
6	-3.062341000	0.523507000	-0.877663000
6	1.275021000	0.965551000	-1.725927000
1	1.401868000	1.723199000	-2.508256000
6	2.186344000	0.649919000	-0.831692000
6	3.567061000	0.736848000	-0.441927000
8	4.490692000	0.303313000	-1.130561000
8	3.725845000	1.304302000	0.780856000
6	5.097378000	1.390200000	1.245524000
1	5.691012000	2.019247000	0.570209000
1	5.035931000	1.843447000	2.238795000
1	5.544402000	0.389639000	1.302044000

Complex 5 (triplet state)

Sum of electronic and zero-point Energies= -4310.203450
Sum of electronic and thermal Energies= -4310.177324
Sum of electronic and thermal Enthalpies= -4310.176380
Sum of electronic and thermal Free Energies= -4310.264808

26	1.293232000	-0.981191000	0.196619000
26	0.232722000	1.482542000	-0.120081000
16	0.345267000	-2.005758000	-1.574602000
16	-0.755303000	-0.335545000	0.839666000
8	-1.778025000	2.734798000	-1.895886000
8	0.320266000	3.672428000	1.881197000
8	2.500121000	2.508148000	-1.705031000
8	2.595509000	0.625533000	2.303812000
8	1.687018000	-3.455532000	1.717807000
8	3.825538000	-1.023233000	-1.316143000
6	-1.002194000	2.215535000	-1.212558000
6	0.298434000	2.793819000	1.128727000
6	1.621714000	2.083949000	-1.079830000
6	2.035000000	0.066447000	1.460996000
6	1.530871000	-2.485129000	1.114675000
6	2.834306000	-1.002236000	-0.727916000
6	-1.333755000	-1.810689000	-1.343168000
1	-1.991886000	-2.301055000	-2.064662000
6	-1.861498000	-1.079031000	-0.316532000
6	-3.331490000	-0.940559000	-0.152623000
8	-4.152901000	-1.447002000	-0.904099000
8	-3.650666000	-0.183230000	0.921853000
6	-5.070100000	0.006488000	1.152555000
1	-5.558338000	-0.960547000	1.325236000
1	-5.135014000	0.636738000	2.043675000

1	-5.531618000	0.503784000	0.290691000

2. Reaction of complex 1 with methyl acrylate 2e.

2a. Singlet state.

Reactive complex (1 + 2e)^{s}

Sum of electronic and zero-point Energies= -4311.393755
Sum of electronic and thermal Energies= -4311.365550
Sum of electronic and thermal Enthalpies= -4311.364605
Sum of electronic and thermal Free Energies= -4311.466465

26	-2.716148000	2.434278000	0.400560000
26	-4.220599000	0.111403000	0.607698000
16	-3.489756000	1.135919000	-1.208043000
16	-2.371530000	0.664619000	1.676626000
8	-3.634194000	-2.643036000	-0.108934000
8	-5.322473000	-0.419065000	3.304825000
8	-6.894632000	0.280549000	-0.654973000
8	-2.534747000	3.990169000	2.914795000
8	-0.000581000	2.895175000	-0.525083000
8	-4.010708000	4.641826000	-1.090342000
6	-3.882093000	-1.552958000	0.179981000
6	-4.892191000	-0.210306000	2.256839000
6	-5.853402000	0.216082000	-0.166696000
6	-2.604993000	3.382845000	1.938588000
6	-1.082079000	2.728094000	-0.156643000
6	-3.510829000	3.780646000	-0.511165000
6	3.355548000	-2.099367000	-2.453129000
1	2.632670000	-2.483624000	-3.173635000
6	3.721851000	-2.828979000	-1.391120000
6	4.692783000	-2.401573000	-0.354669000
8	4.999416000	-3.096722000	0.606589000
8	5.206294000	-1.166182000	-0.576916000
6	6.159574000	-0.701838000	0.408971000
1	7.025483000	-1.375041000	0.451928000
1	6.463249000	0.293891000	0.072761000
1	5.690412000	-0.647967000	1.399798000
1	3.764046000	-1.104590000	-2.631778000
1	3.309627000	-3.824253000	-1.216830000

TS1$^{s}_{3e}$

Sum of electronic and zero-point Energies= -4311.390701
Sum of electronic and thermal Energies= -4311.363983
Sum of electronic and thermal Enthalpies= -4311.363038
Sum of electronic and thermal Free Energies= -4311.452551

26	-0.127091000	1.404209000	0.012266000

26	-1.626655000	-0.832651000	0.069777000
16	-0.634533000	0.078057000	-1.677133000
16	0.182050000	-0.386302000	1.252979000
8	-1.373935000	-3.689450000	-0.444837000
8	-2.999627000	-1.073931000	2.681587000
8	-4.140904000	-0.430655000	-1.441060000
8	-0.234832000	2.859177000	2.586547000
8	2.474983000	2.427200000	-0.816052000
8	-1.699204000	3.446944000	-1.442103000
6	-1.486999000	-2.558576000	-0.238108000
6	-2.464376000	-0.979423000	1.665689000
6	-3.161869000	-0.584880000	-0.853239000
6	-0.193538000	2.289665000	1.585559000
6	1.449725000	2.016265000	-0.481047000
6	-1.082168000	2.653764000	-0.878389000
6	1.838611000	-1.708753000	-1.801838000
1	1.056588000	-2.257168000	-2.324765000
6	2.203487000	-2.048820000	-0.547848000
6	3.356316000	-1.480801000	0.190549000
8	3.726343000	-1.891996000	1.284014000
8	3.981068000	-0.481597000	-0.480535000
6	5.136616000	0.085450000	0.181704000
1	5.903829000	-0.683829000	0.336140000
1	5.503918000	0.865017000	-0.491814000
1	4.851760000	0.515380000	1.150202000
1	2.363137000	-0.932052000	-2.357305000
1	1.711928000	-2.864825000	-0.017781000

Complex 3e (THF)

Sum of electronic and zero-point Energies=	-4311.444358
Sum of electronic and thermal Energies=	-4311.418896
Sum of electronic and thermal Enthalpies=	-4311.417952
Sum of electronic and thermal Free Energies=	-4311.502874

26	-0.091817000	1.286118000	0.004270000
26	-1.460506000	-0.854451000	0.024714000
16	-0.251205000	-0.131867000	-1.756070000
16	0.578677000	-0.639704000	0.990631000
8	-1.702892000	-3.747565000	-0.441281000
8	-2.751740000	-0.774716000	2.666906000
8	-3.955684000	-0.048849000	-1.309536000
8	-0.617005000	2.459203000	2.651111000
8	2.333818000	2.838450000	-0.588432000
8	-1.970133000	3.144476000	-1.285751000
6	-1.593772000	-2.609816000	-0.260933000
6	-2.239342000	-0.801179000	1.630874000
6	-2.970397000	-0.356743000	-0.788681000
6	-0.412911000	1.989904000	1.614386000
6	1.399421000	2.197722000	-0.355832000

6	-1.234592000	2.408505000	-0.781464000
6	1.331686000	-1.074314000	-1.674493000
1	1.215119000	-1.938479000	-2.342069000
6	1.669928000	-1.516121000	-0.256535000
6	3.119585000	-1.346123000	0.192410000
8	3.688093000	-2.140099000	0.921317000
8	3.674787000	-0.215892000	-0.285312000
6	5.042849000	0.039629000	0.133080000
1	5.697435000	-0.771543000	-0.207513000
1	5.317196000	0.985224000	-0.341710000
1	5.093288000	0.122398000	1.225356000
1	2.098355000	-0.409869000	-2.089078000
1	1.430771000	-2.574805000	-0.100594000

Complex 3e (MeCN)

Sum of electronic and zero-point Energies=	-4311.444358
Sum of electronic and thermal Energies=	-4311.418896
Sum of electronic and thermal Enthalpies=	-4311.417952
Sum of electronic and thermal Free Energies=	-4311.502874

26	-0.088617000	1.285059000	0.007254000
26	-1.463654000	-0.851534000	0.022032000
16	-0.247531000	-0.131235000	-1.754922000
16	0.573622000	-0.643824000	0.992908000
8	-1.718904000	-3.742734000	-0.449037000
8	-2.760591000	-0.772595000	2.661303000
8	-3.951785000	-0.034523000	-1.317903000
8	-0.617455000	2.456904000	2.653733000
8	2.342892000	2.831079000	-0.577508000
8	-1.956926000	3.150168000	-1.287166000
6	-1.603317000	-2.605886000	-0.267098000
6	-2.245922000	-0.798875000	1.626135000
6	-2.969099000	-0.347131000	-0.794515000
6	-0.411865000	1.988164000	1.616816000
6	1.406179000	2.192493000	-0.348229000
6	-1.225248000	2.411274000	-0.781100000
6	1.330565000	-1.080626000	-1.671379000
1	1.208330000	-1.946898000	-2.335002000
6	1.668655000	-1.518615000	-0.252548000
6	3.115668000	-1.342881000	0.202996000
8	3.668476000	-2.112985000	0.969837000
8	3.686810000	-0.239008000	-0.312482000
6	5.054414000	0.019867000	0.107014000
1	5.702129000	-0.813257000	-0.190664000
1	5.344032000	0.939471000	-0.407811000
1	5.095510000	0.152406000	1.194712000
1	2.099653000	-0.422009000	-2.090499000
1	1.431644000	-2.577559000	-0.094721000

Anion radical 3e$^{•-}$ (MeCN)

Sum of electronic and zero-point Energies= -4311.570534
Sum of electronic and thermal Energies= -4311.544253
Sum of electronic and thermal Enthalpies= -4311.543309
Sum of electronic and thermal Free Energies= -4311.631139

26	0.078137000	1.409472000	0.013478000
26	-1.573872000	-0.854734000	0.019246000
16	-0.321983000	-0.028803000	-1.741195000
16	0.512840000	-0.635699000	1.008201000
8	-2.003959000	-3.728694000	-0.509405000
8	-2.835845000	-0.785889000	2.663870000
8	-4.030559000	0.116358000	-1.253292000
8	-0.236693000	2.604426000	2.672645000
8	2.590862000	2.795669000	-0.683425000
8	-1.735595000	3.385792000	-1.173020000
6	-1.705244000	-2.611788000	-0.335159000
6	-2.331951000	-0.798640000	1.610560000
6	-3.046492000	-0.250356000	-0.742183000
6	-0.123197000	2.122445000	1.615412000
6	1.662252000	2.132758000	-0.432584000
6	-1.031118000	2.586810000	-0.694114000
6	1.208231000	-1.060778000	-1.683619000
1	1.062010000	-1.896876000	-2.382079000
6	1.503809000	-1.580606000	-0.277121000
6	2.957562000	-1.585225000	0.167713000
8	3.433652000	-2.444010000	0.896485000
8	3.656408000	-0.528188000	-0.297401000
6	5.036773000	-0.444817000	0.141980000
1	5.594180000	-1.329602000	-0.189117000
1	5.435018000	0.459537000	-0.326251000
1	5.083564000	-0.368038000	1.235211000
1	2.021057000	-0.426574000	-2.055026000
1	1.149634000	-2.612171000	-0.162637000

2b. Triplet state.

Reactive complex (1 + 2e)^{t}

Sum of electronic and zero-point Energies= -4311.379395
Sum of electronic and thermal Energies= -4311.350902
Sum of electronic and thermal Enthalpies= -4311.349958
Sum of electronic and thermal Free Energies= -4311.457336

26	2.381621000	1.204296000	-0.016125000
26	1.511987000	-1.352077000	0.000861000
16	0.636563000	0.379359000	1.090558000
16	1.168272000	0.179354000	-1.577018000

8	-0.783524000	-3.162410000	-0.246309000
8	3.303619000	-2.877103000	-1.782655000
8	2.570567000	-2.502410000	2.506040000
8	4.723559000	1.289518000	-1.813107000
8	1.670485000	4.037167000	-0.294550000
8	3.934361000	1.499918000	2.477437000
6	0.122895000	-2.454002000	-0.148457000
6	2.610546000	-2.284437000	-1.076377000
6	2.165229000	-2.058555000	1.521620000
6	3.816321000	1.255230000	-1.101711000
6	1.954972000	2.923570000	-0.184793000
6	3.337330000	1.383799000	1.497340000
6	-6.704581000	2.443560000	-0.795971000
1	-6.487029000	3.512119000	-0.800588000
6	-7.133132000	1.834146000	0.317066000
6	-7.455855000	0.391019000	0.430820000
8	-7.843406000	-0.130759000	1.469324000
8	-7.279411000	-0.291755000	-0.727121000
6	-7.581224000	-1.706612000	-0.667720000
1	-8.632594000	-1.862676000	-0.394786000
1	-7.384574000	-2.087771000	-1.673991000
1	-6.936015000	-2.205314000	0.066582000
1	-6.559671000	1.896447000	-1.727693000
1	-7.276279000	2.386441000	1.247331000

TS1$^{t}_{3e}$

Sum of electronic and zero-point Energies= -4311.390701
Sum of electronic and thermal Energies= -4311.363983
Sum of electronic and thermal Enthalpies= -4311.363038
Sum of electronic and thermal Free Energies= -4311.452551

26	-0.127723000	1.404457000	0.012444000
26	-1.626665000	-0.832968000	0.069655000
16	-0.634304000	0.077936000	-1.676942000
16	0.181513000	-0.385791000	1.253480000
8	-1.372551000	-3.689817000	-0.443926000
8	-3.000360000	-1.073936000	2.681125000
8	-4.140526000	-0.432928000	-1.442342000
8	-0.234823000	2.859916000	2.586481000
8	2.473797000	2.427785000	-0.817175000
8	-1.700913000	3.446347000	-1.441940000
6	-1.486149000	-2.558917000	-0.237621000
6	-2.464853000	-0.979571000	1.665349000
6	-3.161647000	-0.586351000	-0.854054000
6	-0.193841000	2.290209000	1.585591000
6	1.448757000	2.016766000	-0.481609000
6	-1.083434000	2.653521000	-0.878215000
6	1.838854000	-1.709194000	-1.800538000
1	1.056494000	-2.257813000	-2.322735000

6	2.204089000	-2.048328000	-0.546402000
6	3.357429000	-1.480064000	0.191008000
8	3.728019000	-1.890707000	1.284490000
8	3.981988000	-0.481371000	-0.481017000
6	5.138212000	0.085635000	0.180075000
1	5.905112000	-0.683912000	0.334722000
1	5.505533000	0.864436000	-0.494321000
1	4.854138000	0.516547000	1.148365000
1	2.363381000	-0.933083000	-2.356827000
1	1.712545000	-2.863776000	-0.015467000

I1$^{t}_{3e}$

Sum of electronic and zero-point Energies= -4311.372087
Sum of electronic and thermal Energies= -4311.345194
Sum of electronic and thermal Enthalpies= -4311.344250
Sum of electronic and thermal Free Energies= -4311.434906

26	-1.721325000	-0.826439000	-0.117733000
26	-0.156114000	1.268979000	0.041149000
16	0.101419000	-0.651756000	1.251481000
16	-0.367323000	-0.077488000	-1.700036000
8	2.588656000	2.307322000	0.011572000
8	-1.228600000	3.399664000	-1.685368000
8	-1.070447000	2.633296000	2.504901000
8	-3.944977000	-0.277539000	-1.969115000
8	-1.813523000	-3.744934000	-0.429711000
8	-3.465428000	-0.563291000	2.259991000
6	1.514134000	1.882037000	0.025272000
6	-0.816423000	2.563113000	-1.005320000
6	-0.714933000	2.092163000	1.549876000
6	-3.074579000	-0.483152000	-1.239325000
6	-1.775362000	-2.596606000	-0.304227000
6	-2.778648000	-0.662580000	1.337899000
6	1.479204000	-1.725974000	0.552542000
1	1.165574000	-2.741725000	0.843958000
6	2.761624000	-1.366554000	1.178645000
6	3.956249000	-0.943741000	0.466194000
8	5.018989000	-0.662849000	1.022016000
8	3.782304000	-0.901220000	-0.885824000
6	4.942676000	-0.490105000	-1.645410000
1	5.775248000	-1.185283000	-1.476920000
1	4.627871000	-0.511841000	-2.692645000
1	5.250979000	0.522505000	-1.355311000
1	1.484733000	-1.636904000	-0.538533000
1	2.864042000	-1.420066000	2.262938000

TS2$^{t}_{3e}$

Sum of electronic and zero-point Energies= -4311.348729

Sum of electronic and thermal Energies= -4311.322333
Sum of electronic and thermal Enthalpies= -4311.321389
Sum of electronic and thermal Free Energies= -4311.410112

26	0.966752000	1.191594000	0.046092000
26	-2.018016000	-0.392577000	0.082244000
16	-0.366566000	0.067161000	-1.491262000
16	-0.285384000	-0.007209000	1.430599000
8	-3.619357000	-1.888355000	-1.905734000
8	-3.543829000	-1.383920000	2.405269000
8	-3.574948000	2.092316000	-0.166773000
8	2.663309000	1.977775000	2.330634000
8	3.073920000	1.601241000	-1.988917000
8	-0.203182000	3.866371000	-0.276547000
6	-2.995487000	-1.305619000	-1.126318000
6	-2.949358000	-1.008170000	1.489926000
6	-2.958463000	1.117080000	-0.091178000
6	2.011617000	1.665208000	1.431233000
6	2.254224000	1.443984000	-1.190207000
6	0.256664000	2.810555000	-0.172876000
6	0.523901000	-1.603726000	-1.660062000
1	-0.166242000	-2.174801000	-2.296847000
6	0.797443000	-2.311801000	-0.403518000
6	2.106208000	-2.440845000	0.220918000
8	2.323021000	-3.121394000	1.223391000
8	3.078028000	-1.740951000	-0.432648000
6	4.405286000	-1.852486000	0.132315000
1	4.737261000	-2.898611000	0.120589000
1	5.046552000	-1.237862000	-0.505763000
1	4.415147000	-1.480515000	1.164714000
1	1.436483000	-1.366606000	-2.217426000
1	0.006953000	-2.885032000	0.077828000

Complex 6 (triplet state)

Sum of electronic and zero-point Energies= -4311.392450
Sum of electronic and thermal Energies= -4311.365960
Sum of electronic and thermal Enthalpies= -4311.365016
Sum of electronic and thermal Free Energies= -4311.454462

26	0.339510000	1.410979000	0.050314000
26	-1.700973000	-0.820903000	-0.037494000
16	-0.364293000	0.127238000	-1.716722000
16	0.390913000	-0.678564000	1.011672000
8	-3.777480000	-1.478341000	-2.021901000
8	-2.623645000	-2.801854000	1.945807000
8	-3.326020000	1.375249000	1.161663000
8	0.408369000	2.667390000	2.712509000
8	3.093987000	2.242263000	-0.695912000
8	-0.922174000	3.794623000	-1.133513000

6	-2.959803000	-1.234972000	-1.241327000
6	-2.261592000	-2.035964000	1.159018000
6	-2.638633000	0.572386000	0.692097000
6	0.370909000	2.165095000	1.670079000
6	2.042374000	1.853794000	-0.410077000
6	-0.442000000	2.850202000	-0.666472000
6	0.936951000	-1.182026000	-1.704653000
1	0.612243000	-1.967653000	-2.399917000
6	1.170199000	-1.761600000	-0.303984000
6	2.622881000	-2.011447000	0.101433000
8	2.973285000	-2.969841000	0.767359000
8	3.449330000	-1.043798000	-0.338115000
6	4.843799000	-1.180888000	0.047424000
1	5.253985000	-2.116253000	-0.351588000
1	5.349701000	-0.316626000	-0.390736000
1	4.934117000	-1.172831000	1.140286000
1	1.844025000	-0.713048000	-2.102039000
1	0.662296000	-2.727349000	-0.192945000

1 Gaussian 09, Revision D.01: M. J. Frisch, G. W. Trucks, H. B. Schlegel, G. E. Scuseria, M. A. Robb, J. R. Cheeseman, G. Scalmani, V. Barone, B. Mennucci, G. A. Petersson, H. Nakatsuji, M. Caricato, X. Li, H. P. Hratchian, A. F. Izmaylov, J. Bloino, G. Zheng, J. L. Sonnenberg, M. Hada, M. Ehara, K. Toyota, R. Fukuda, J. Hasegawa, M. Ishida, T. Nakajima, Y. Honda, O. Kitao, H. Nakai, T. Vreven, J. A. Montgomery, Jr., J. E. Peralta, F. Ogliaro, M. Bearpark, J. J. Heyd, E. Brothers, K. N. Kudin, V. N. Staroverov, T. Keith, R. Kobayashi, J. Normand, K. Raghavachari, A. Rendell, J. C. Burant, S. S. Iyengar, J. Tomasi, M. Cossi, N. Rega, J. M. Millam, M. Klene, J. E. Knox, J. B. Cross, V. Bakken, C. Adamo, J. Jaramillo, R. Gomperts, R. E. Stratmann, O. Yazyev, A. J. Austin, R. Cammi, C. Pomelli, J. W. Ochterski, R. L. Martin, K. Morokuma, V. G. Zakrzewski, G. A. Voth, P. Salvador, J. J. Dannenberg, S. Dapprich, A. D. Daniels, O. Farkas, J. B. Foresman, J. V. Ortiz, J. Cioslowski, D. J. Fox, Gaussian, Inc., Wallingford CT, 2013.

2 a) A. D. Becke, *Phys. Rev. A Gen. Phys.*, 1988, **38**, 3098. b) F. Weigend, R. Ahlrichs, *Phys. Chem. Chem. Phys.* 2005, **7**, 3297. C) F. Weigend, *Phys. Chem. Chem. Phys.*, 2008, **8**, 1057.

3 a) V. Barone, M. J. Cossi, *Phys. Chem. A.*, 1988, **102**, 1995. b) M. Cossi, N. Rega, N. G. Scalmani, V. J. Barone, *Comp. Chem.*, 2003, **24**, 669.

4 Optimizations of TS1s3g and TS1s3e were performed using both, UBP86 + guess(mix,always) and BP86 functionals in order to cover the possibility of biradical singlet TS's in accordance with the previously described singlet biradical states for our starting complex 1. Both calculations converged to the same restricted RBP86 structure discarding the implication of biradical TS's or intermediates in the photochemical reaction of complex 1 with olefins or akynes 2.

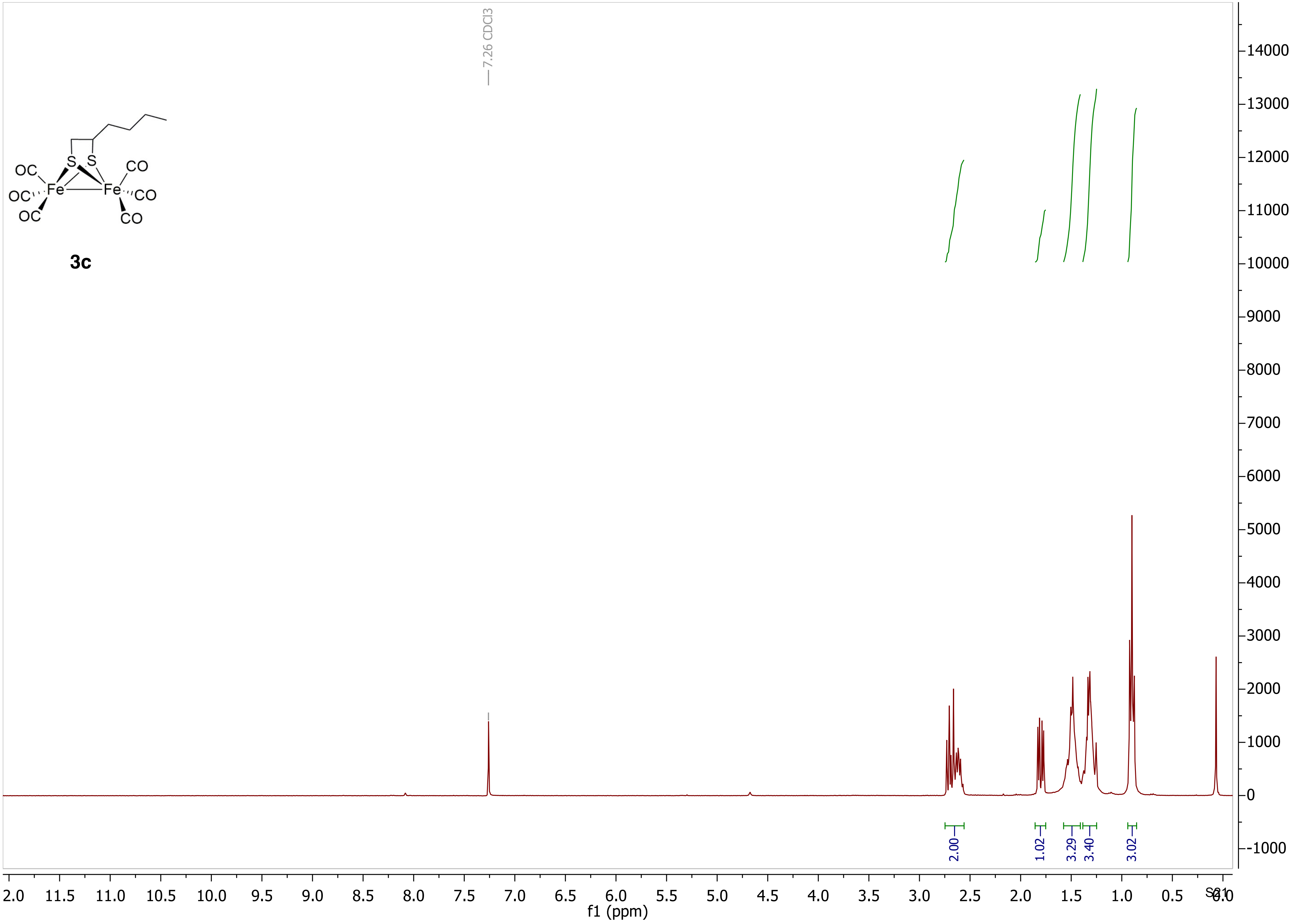
OC
OC
OC
Fe
S
S
Fe
CO
CO
CO
3c
7.26 CDCl3
2.00
1.02
3.29
3.40
3.02
f1 (ppm)
2.0 11.5 11.0 10.5 10.0 9.5 9.0 8.5 8.0 7.5 7.0 6.5 6.0 5.5 5.0 4.5 4.0 3.5 3.0 2.5 2.0 1.5 1.0 0.5 0.0
14000 13000 12000 11000 10000 9000 8000 7000 6000 5000 4000 3000 2000 1000 0 -1000

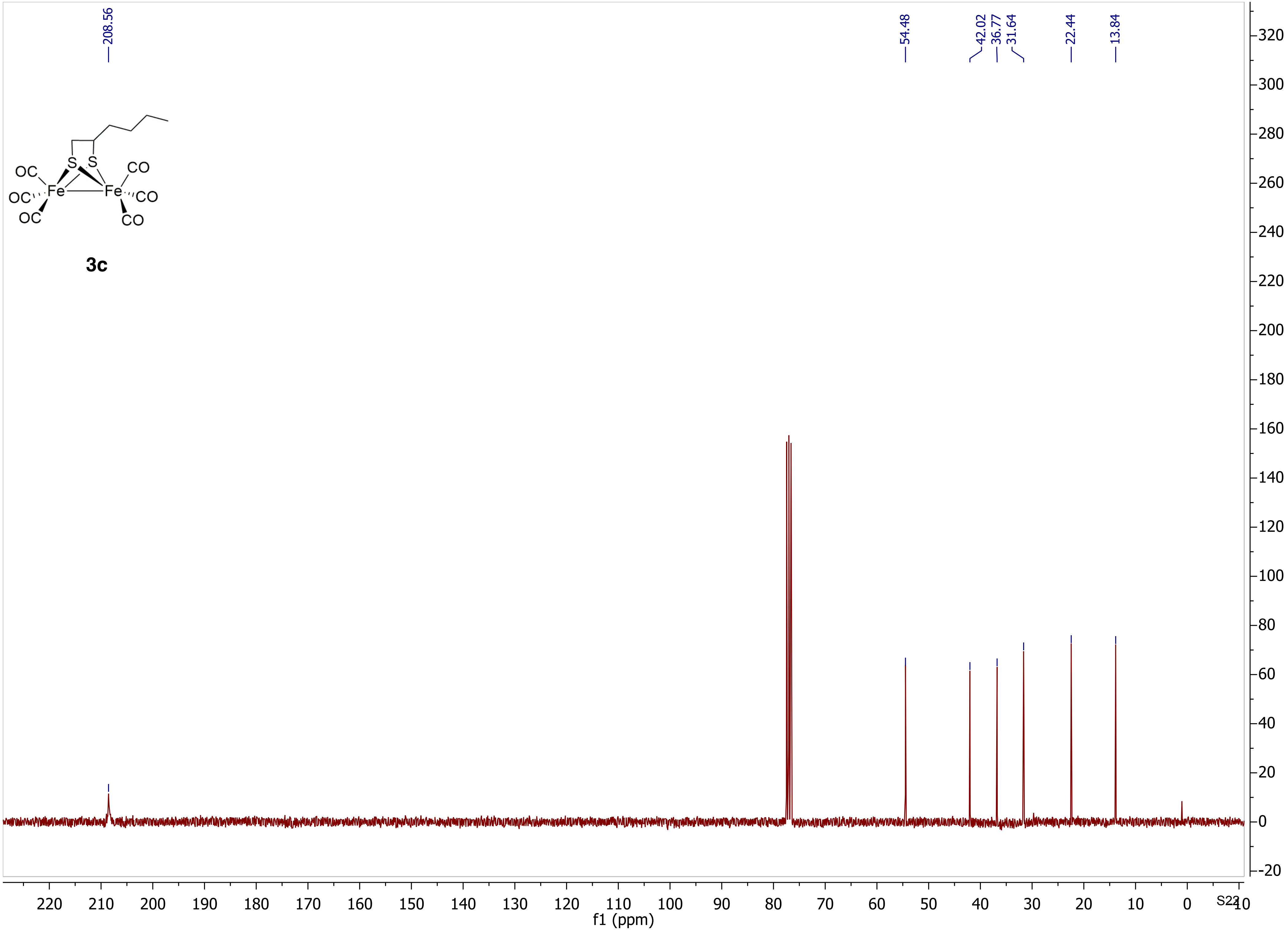
208.56
54.48
42.02
36.77
31.64
22.44
13.84
OC
OC
OC
CO
CO
CO
Fe
Fe
S
S
3c
f1 (ppm)

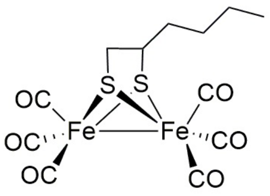
OC
OC
OC
Fe
S
S
Fe
CO
CO
CO

3c

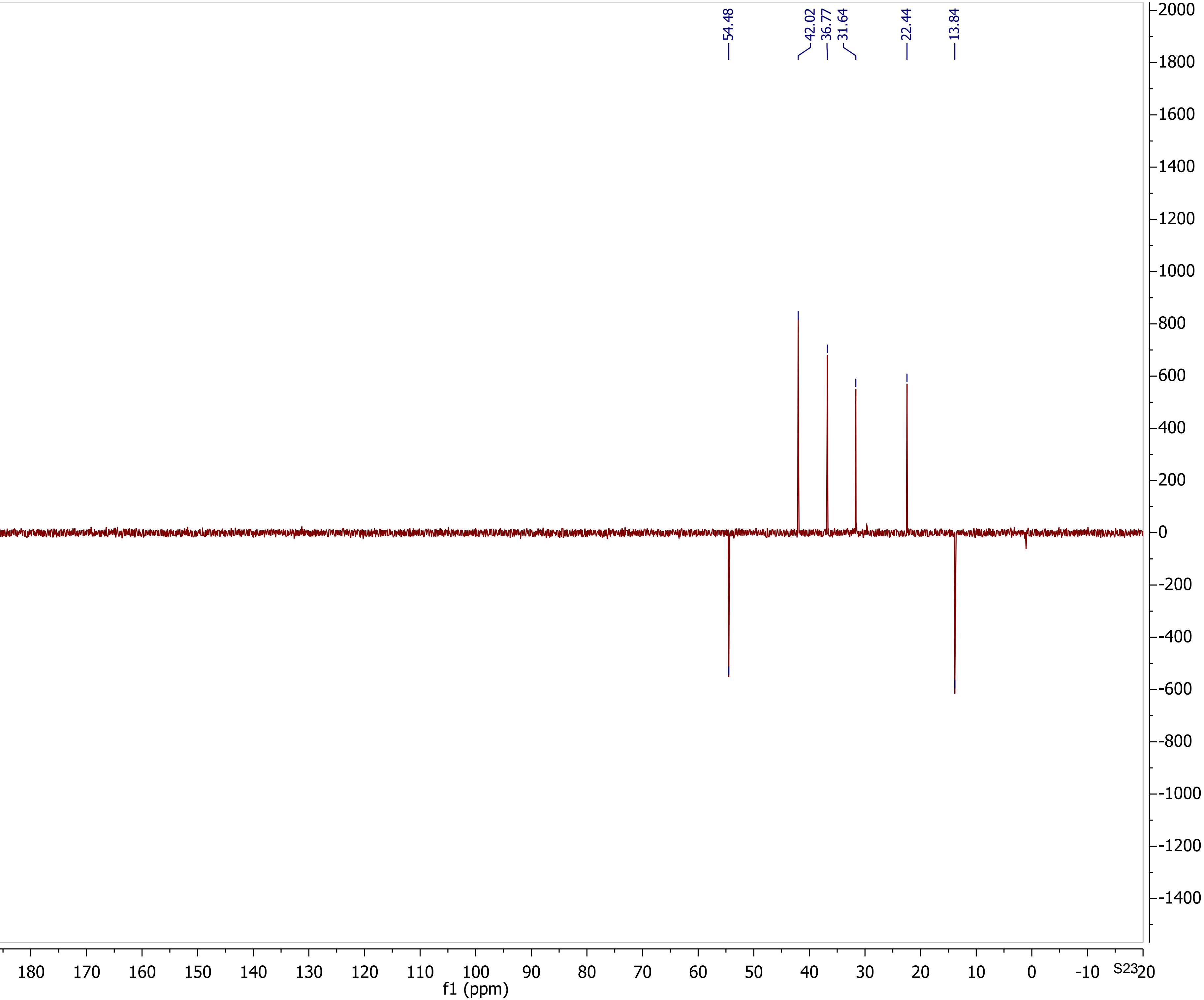
54.48
42.02
36.77
31.64
22.44
13.84
2000
1800
1600
1400
1200
1000
800
600
400
200
0
-200
-400
-600
-800
-1000
-1200
-1400
210 200 190 180 170 160 150 140 130 120 110 100 90 80 70 60 50 40 30 20 10 0 -10 -20
f1 (ppm)

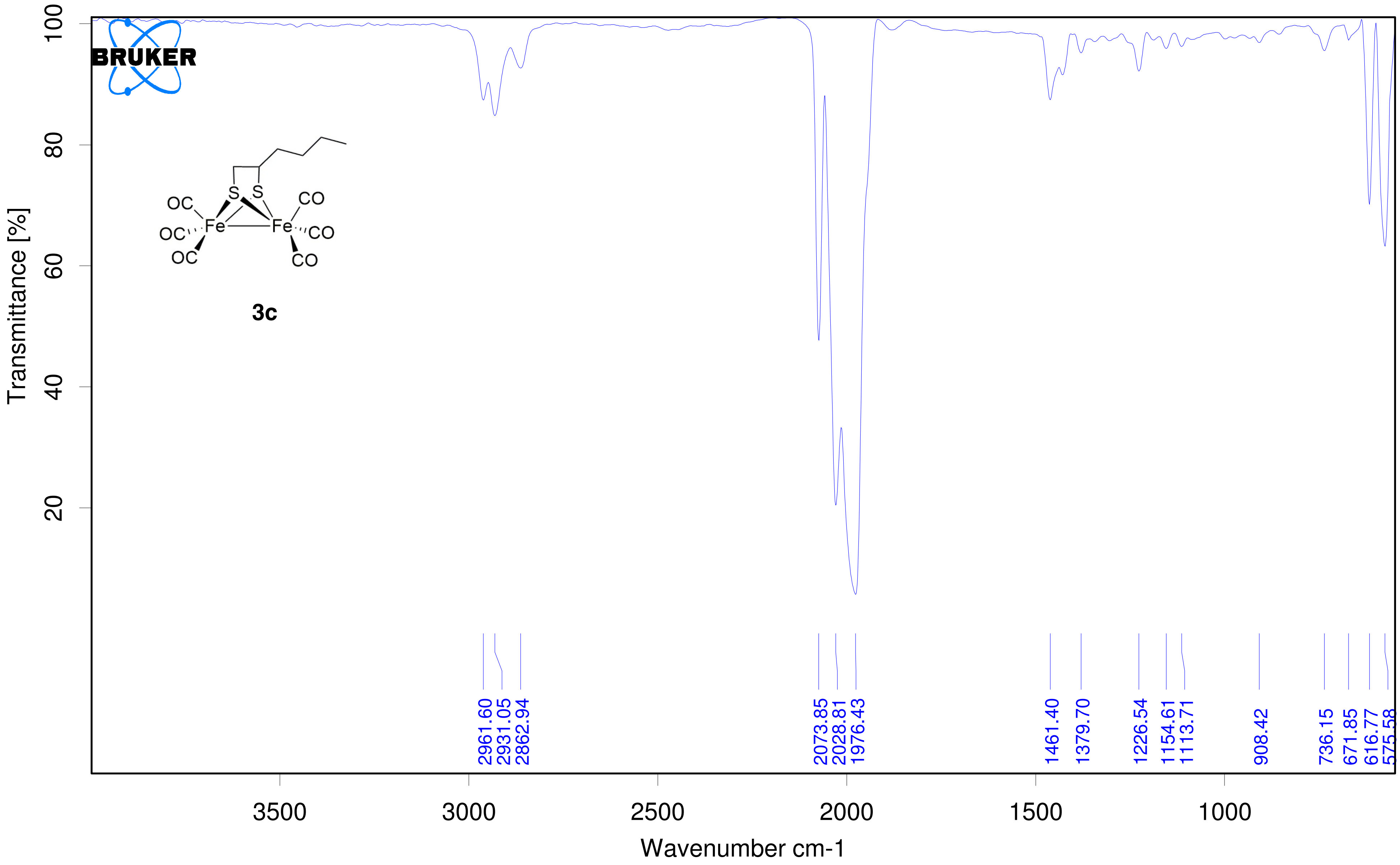
BRUKER
Transmittance [%]
100
80
60
40
20
3c
OC
OC
OC
Fe
S
S
Fe
CO
CO
CO
2961.60
2931.05
2862.94
2073.85
2028.81
1976.43
1461.40
1379.70
1226.54
1154.61
1113.71
908.42
736.15
671.85
616.77
575.58
3500
3000
2500
2000
1500
1000
Wavenumber cm-1

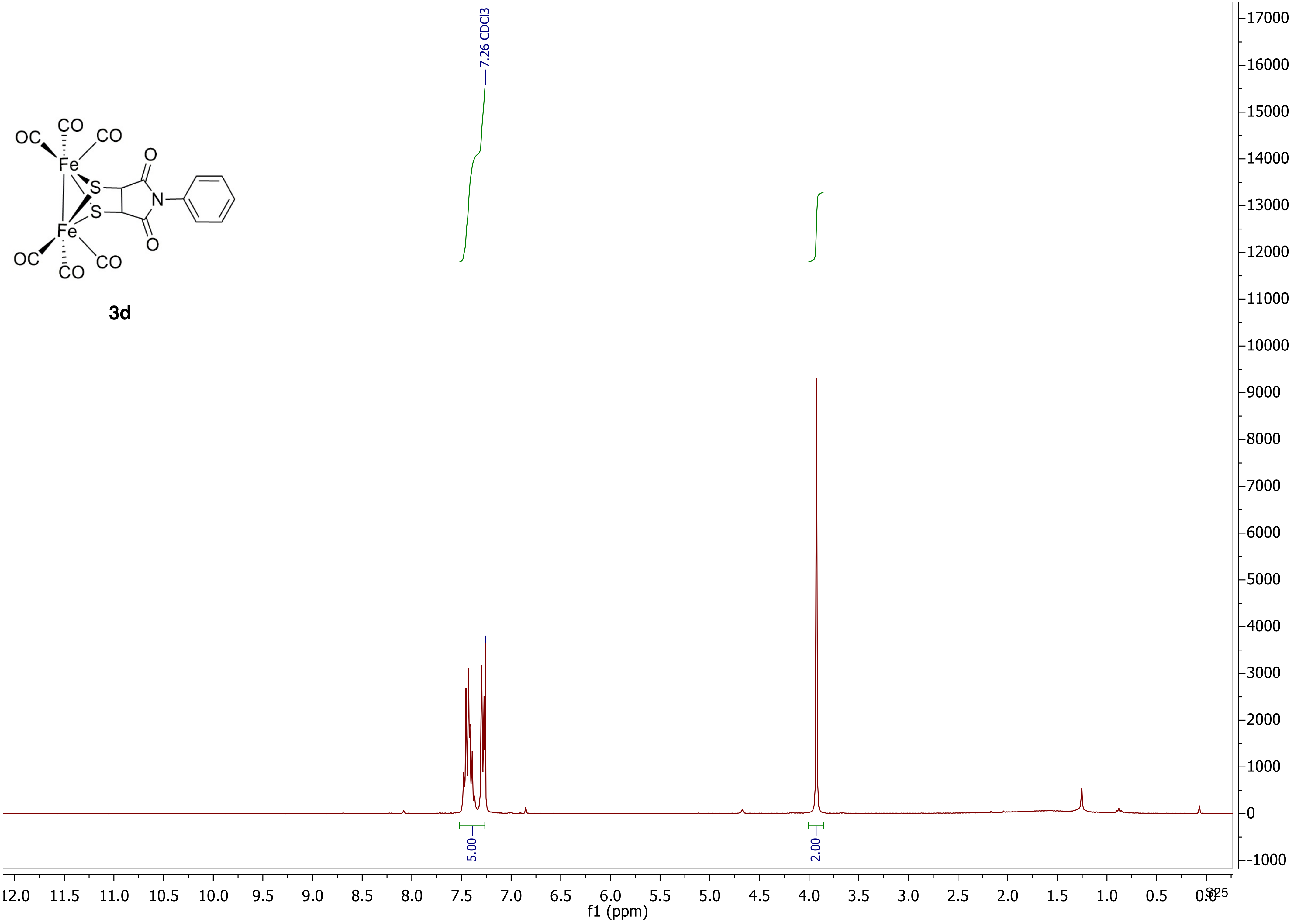

OC
CO
CO
Fe
S
S
Fe
O
N
O
OC
CO
CO
3d
7.26 CDCl3
5.00
2.00
f1 (ppm)

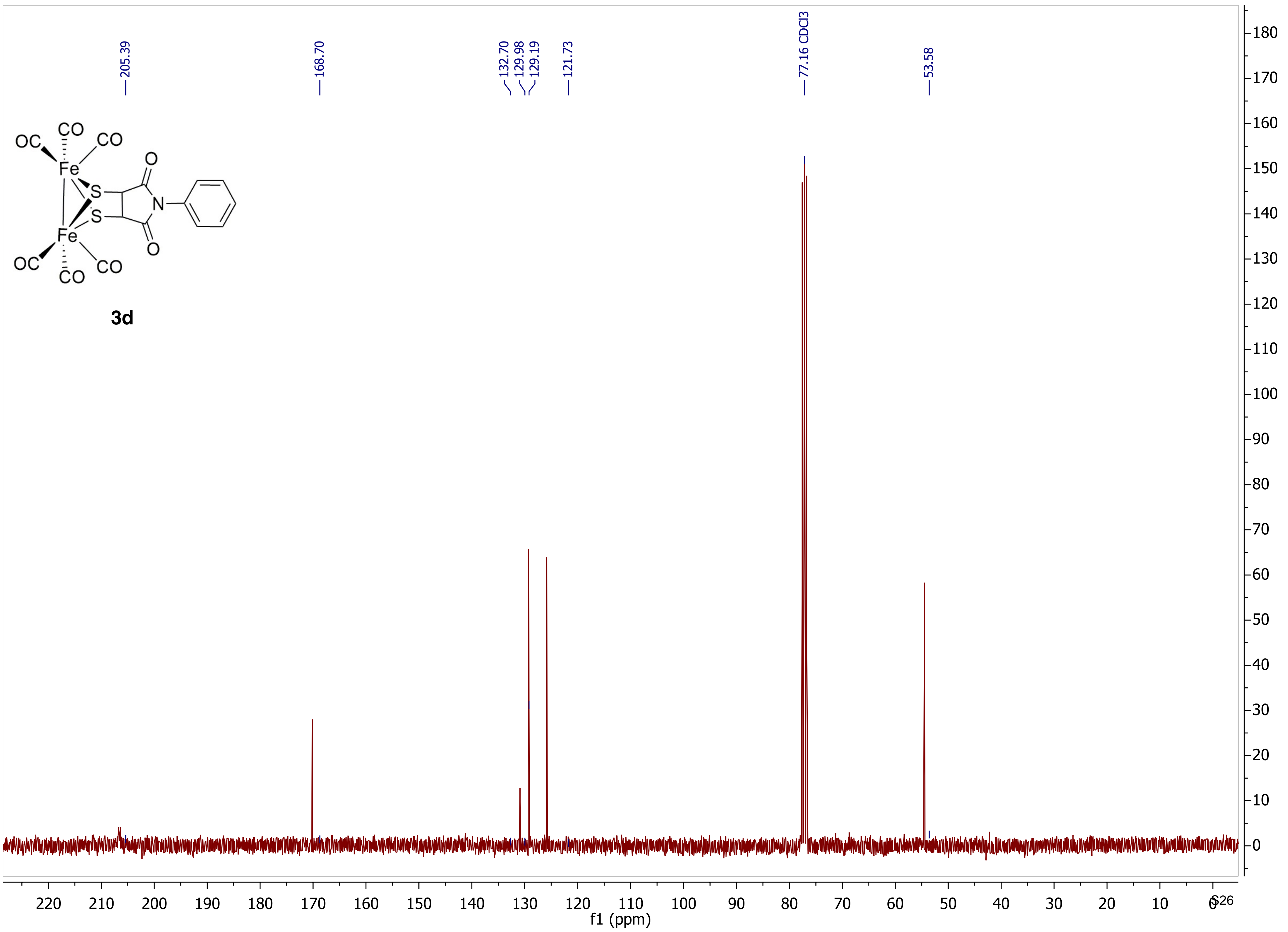

3d

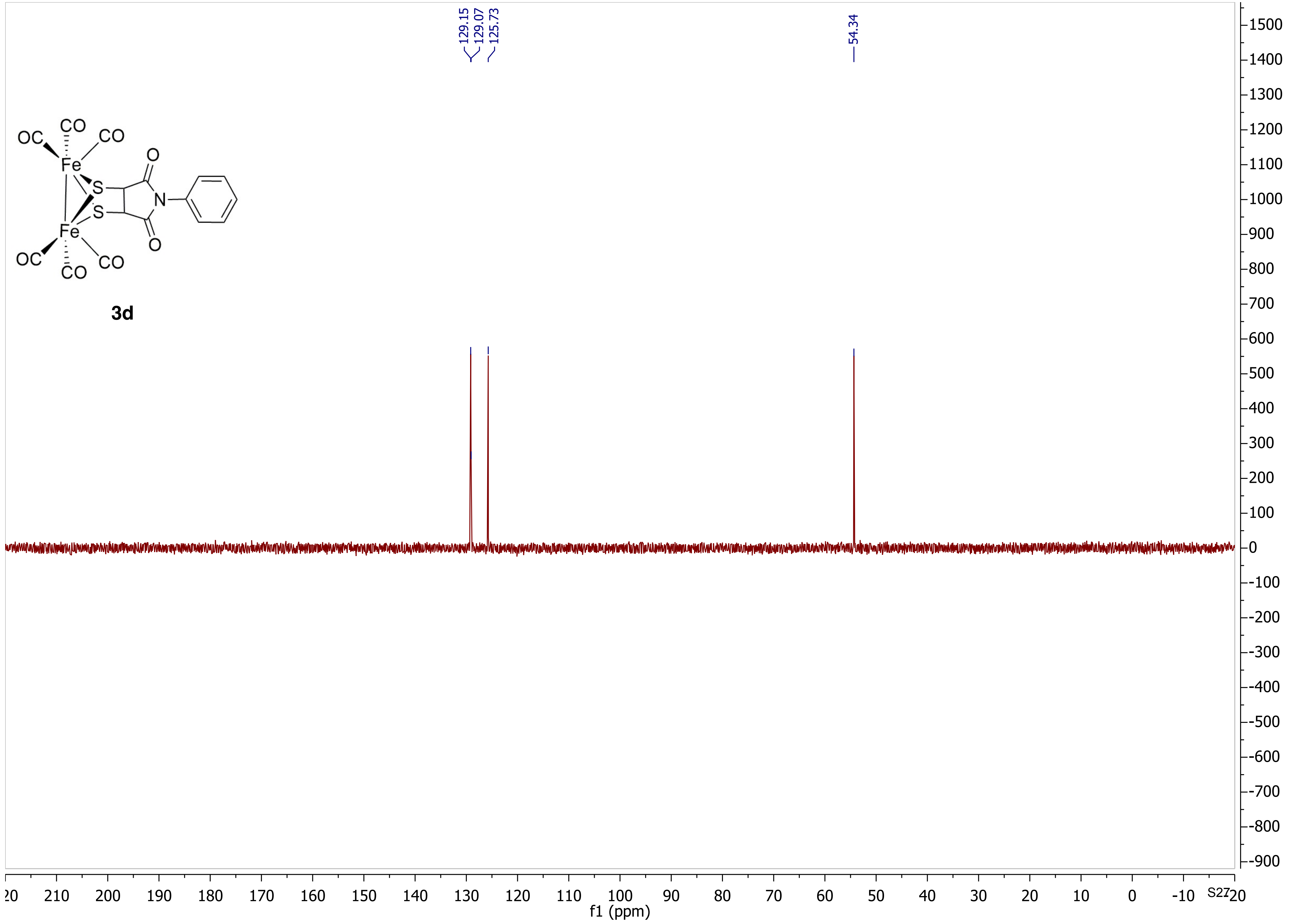

129.15
129.07
125.73
54.34
OC
CO
CO
Fe
O
S
S
N
Fe
O
OC
CO
CO
3d
1500
1400
1300
1200
1100
1000
900
800
700
600
500
400
300
200
100
0
-100
-200
-300
-400
-500
-600
-700
-800
-900
210
200
190
180
170
160
150
140
130
120
110
100
90
80
70
60
50
40
30
20
10
0
-10
f1 (ppm)

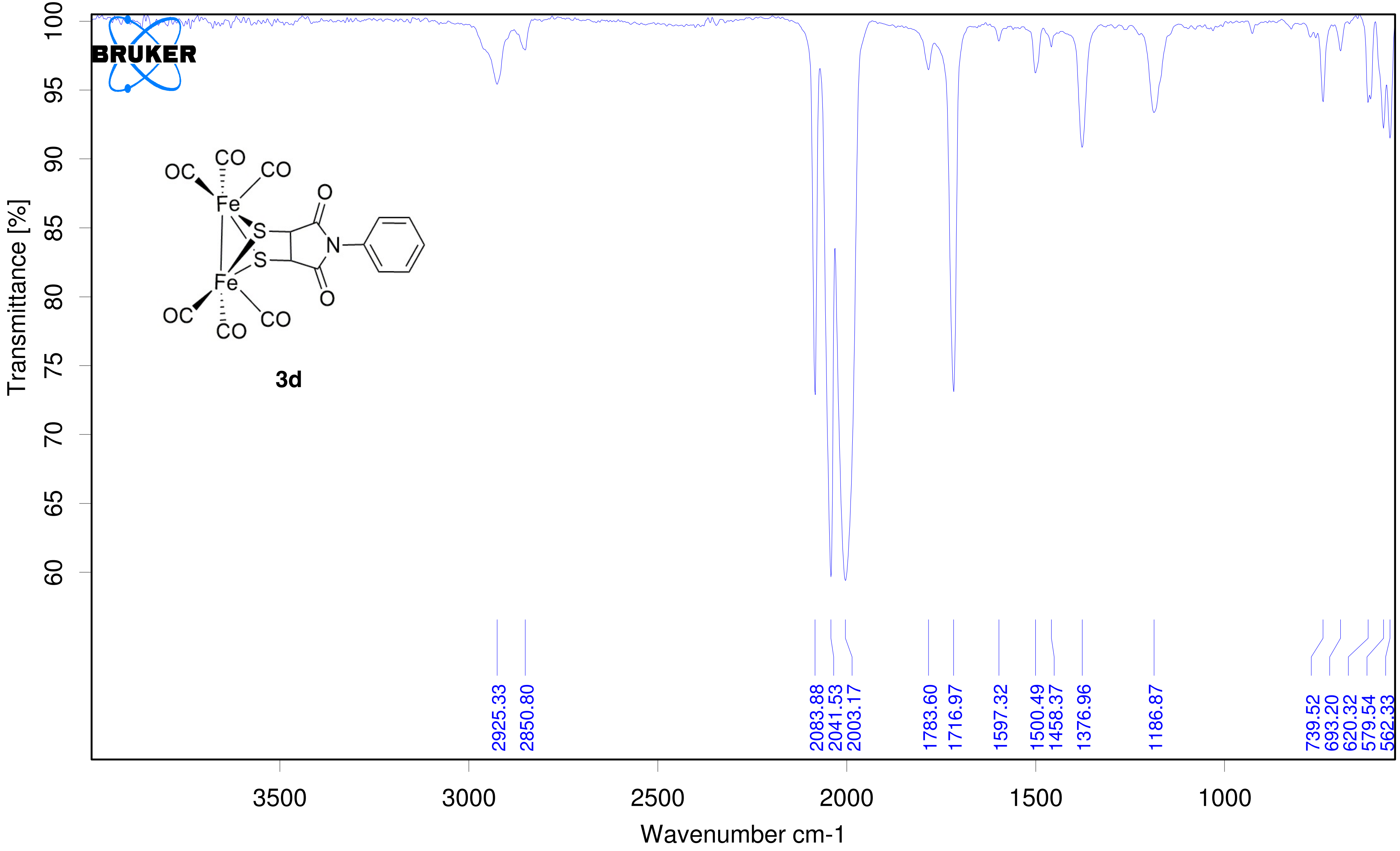
BRUKER
Transmittance [%]
Wavenumber cm-1
3d
2925.33
2850.80
2083.88
2041.53
2003.17
1783.60
1716.97
1597.32
1500.49
1458.37
1376.96
1186.87
739.52
693.20
620.32
579.54
562.33

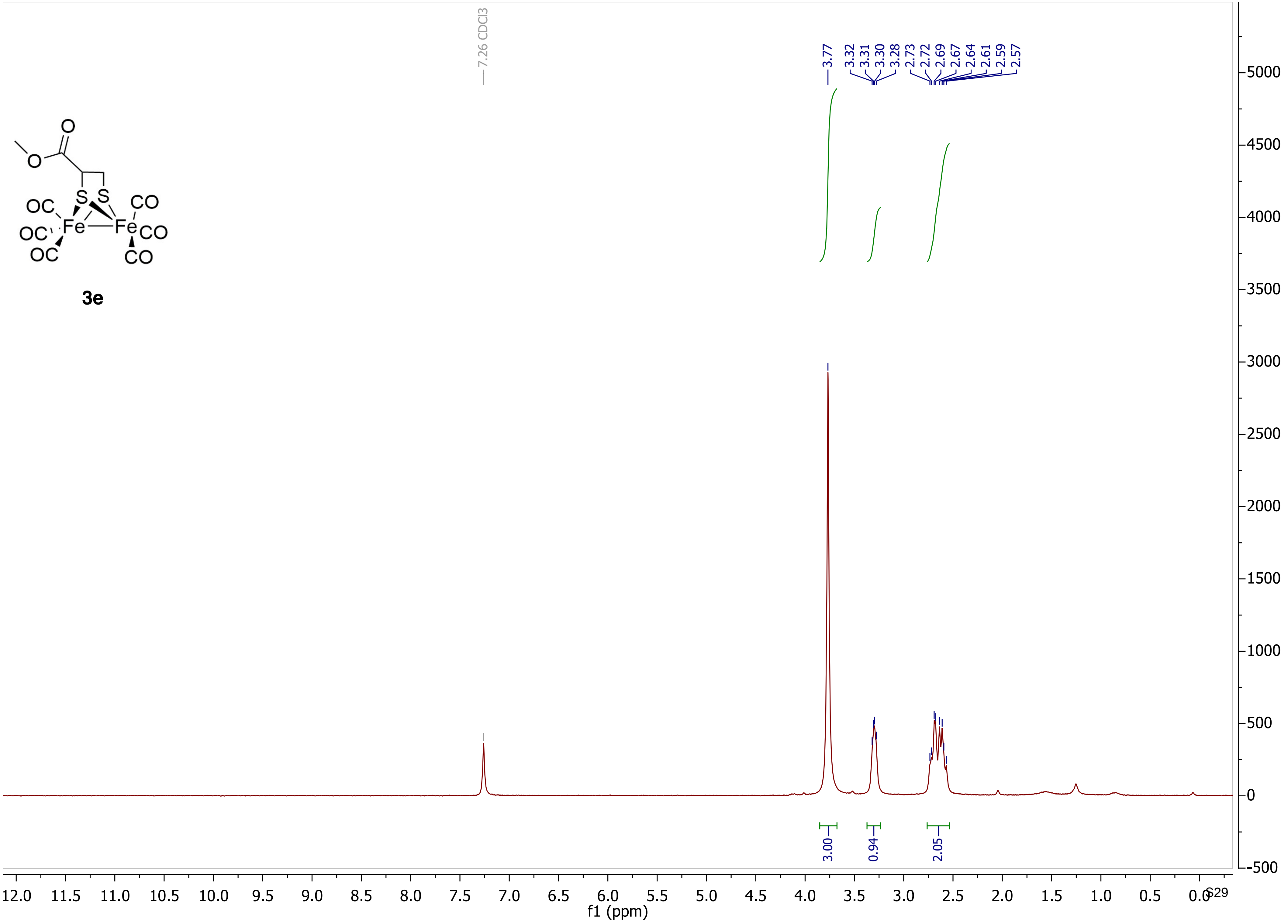
7.26 CDCl3
3.77
3.32
3.31
3.30
3.28
2.73
2.72
2.69
2.67
2.64
2.61
2.59
2.57
3.00
0.94
2.05
f1 (ppm)

3e

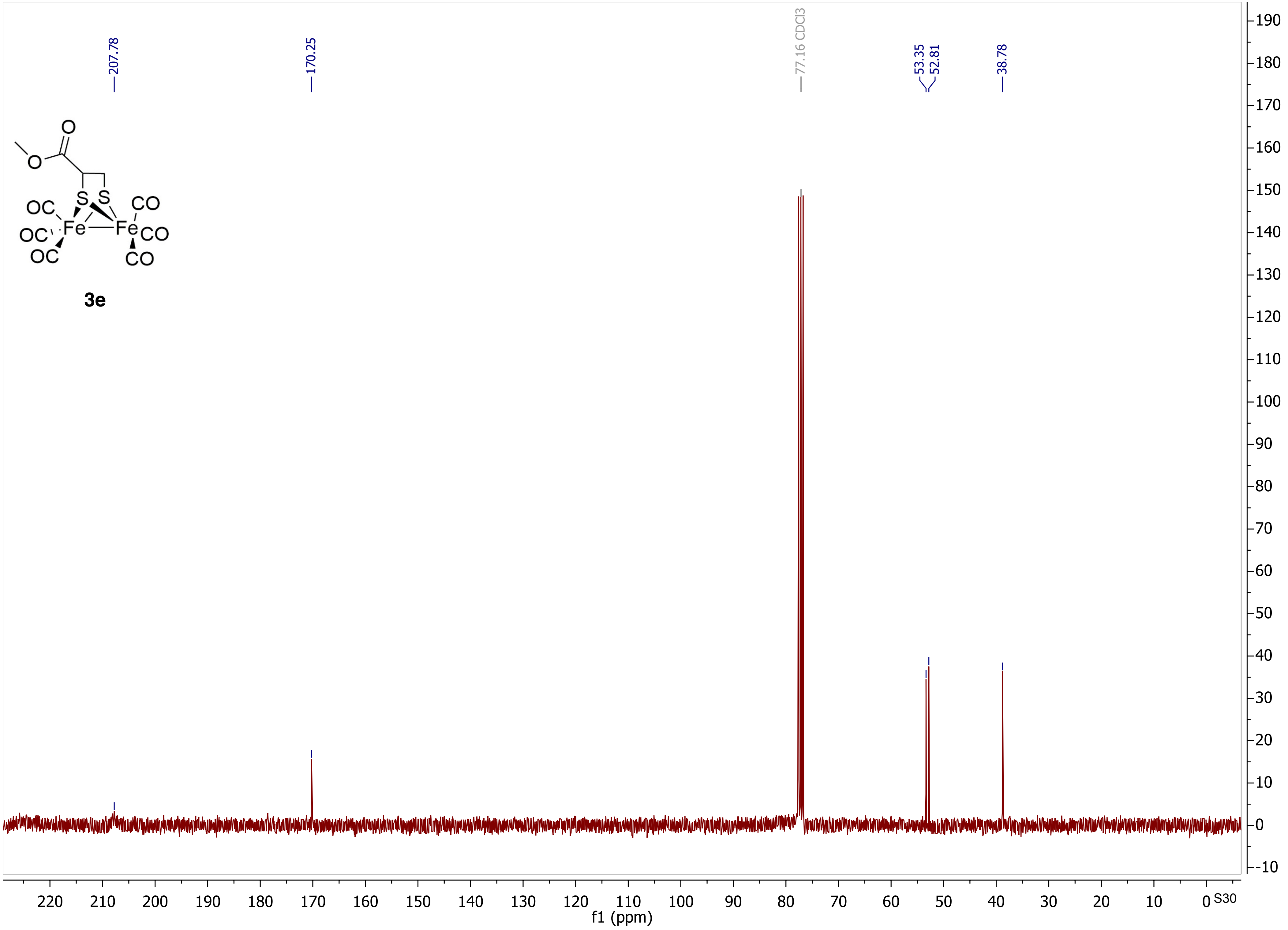
3e
207.78
170.25
77.16 CDCl3
53.35
52.81
38.78
f1 (ppm)

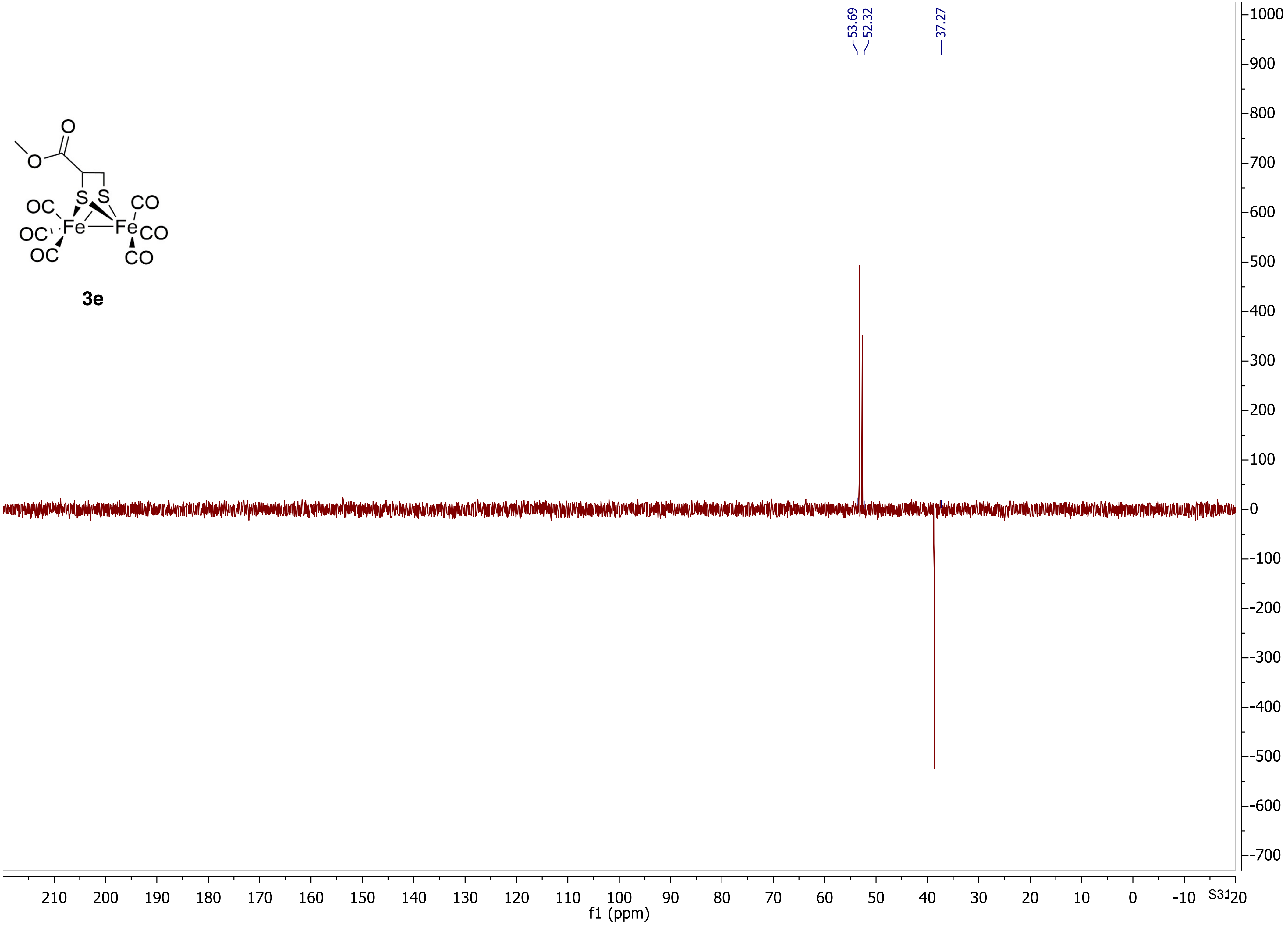

53.69
52.32
37.27
3e
f1 (ppm)

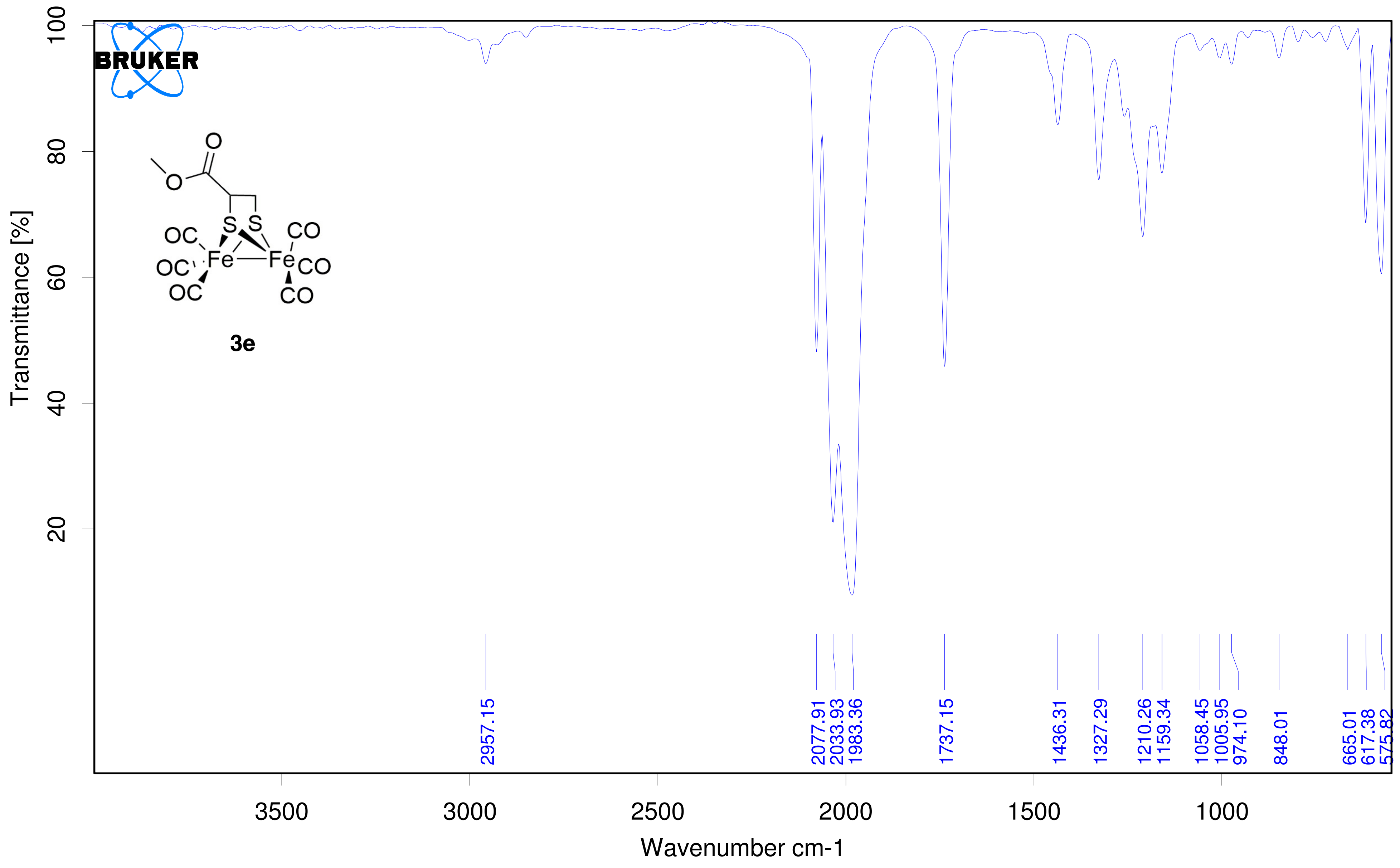
BRUKER
Transmittance [%]
Wavenumber cm-1
2957.15
2077.91
2033.93
1983.36
1737.15
1436.31
1327.29
1210.26
1159.34
1058.45
1005.95
974.10
848.01
665.01
617.38
575.82
3e

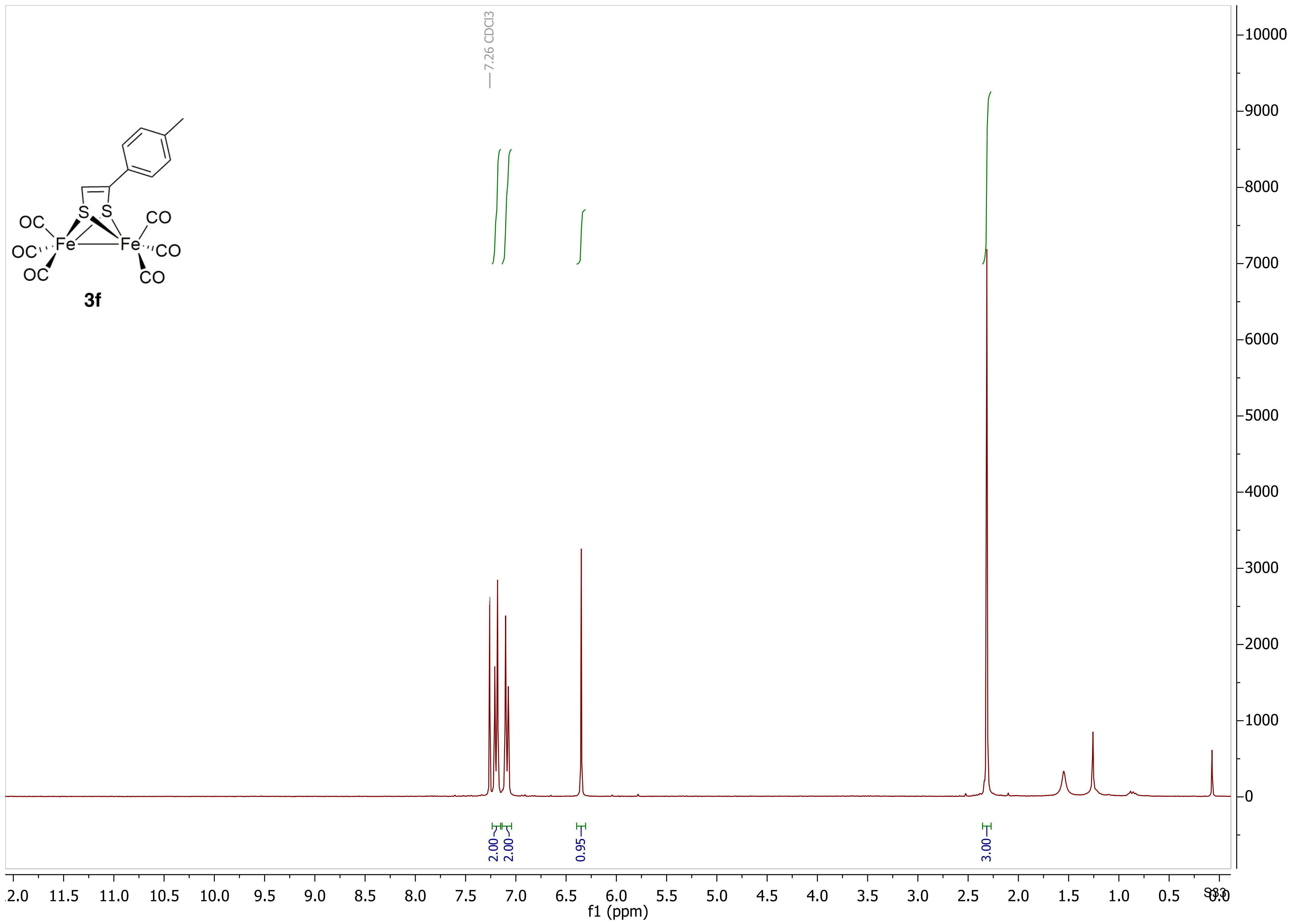

7.26 CDCl3
OC
OC
OC
CO
CO
CO
Fe
Fe
S
S
3f
2.00
2.00
0.95
3.00
f1 (ppm)

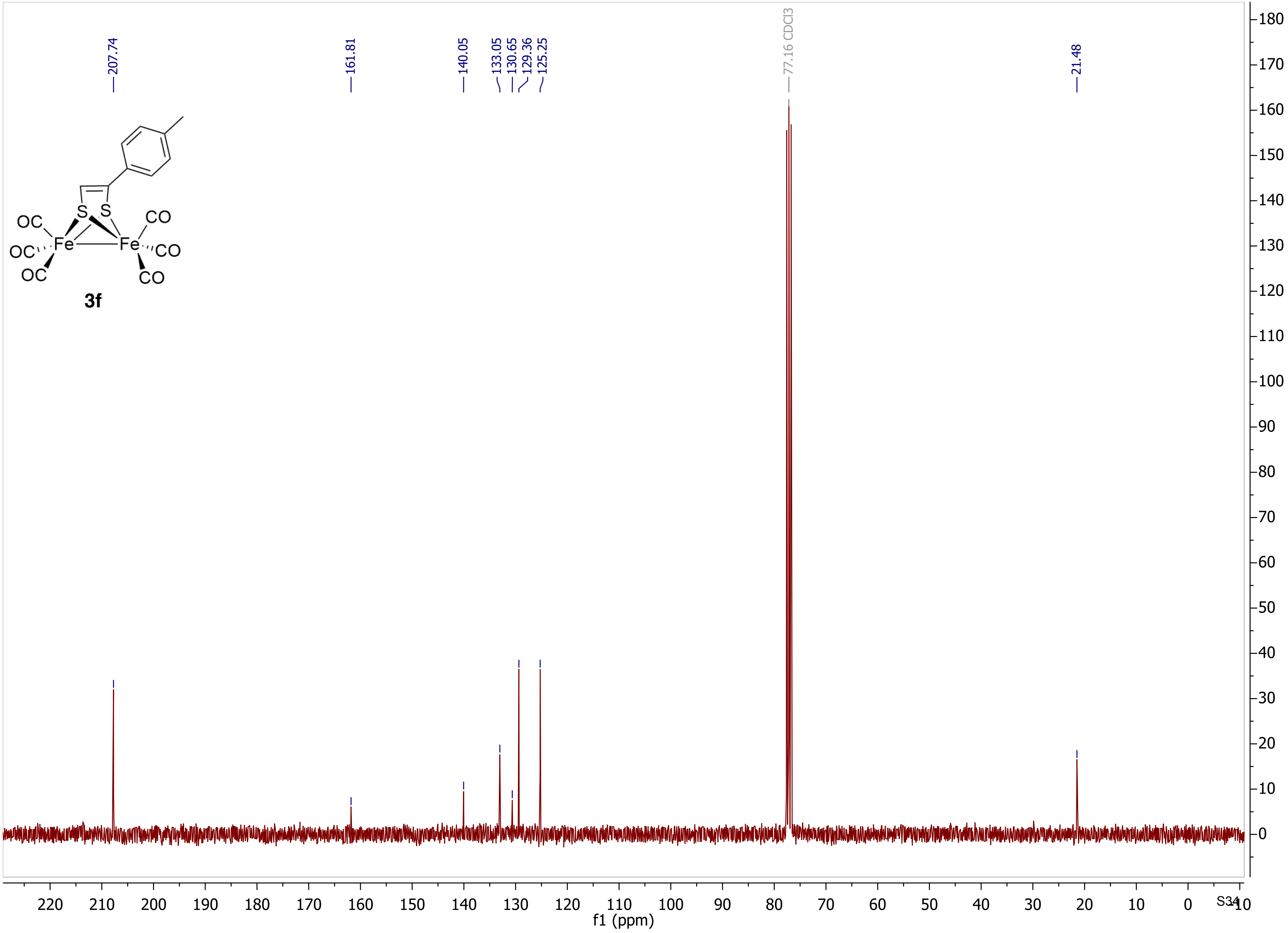
207.74
161.81
140.05
133.05
130.65
129.36
125.25
77.16 CDCl3
21.48
3f
f1 (ppm)

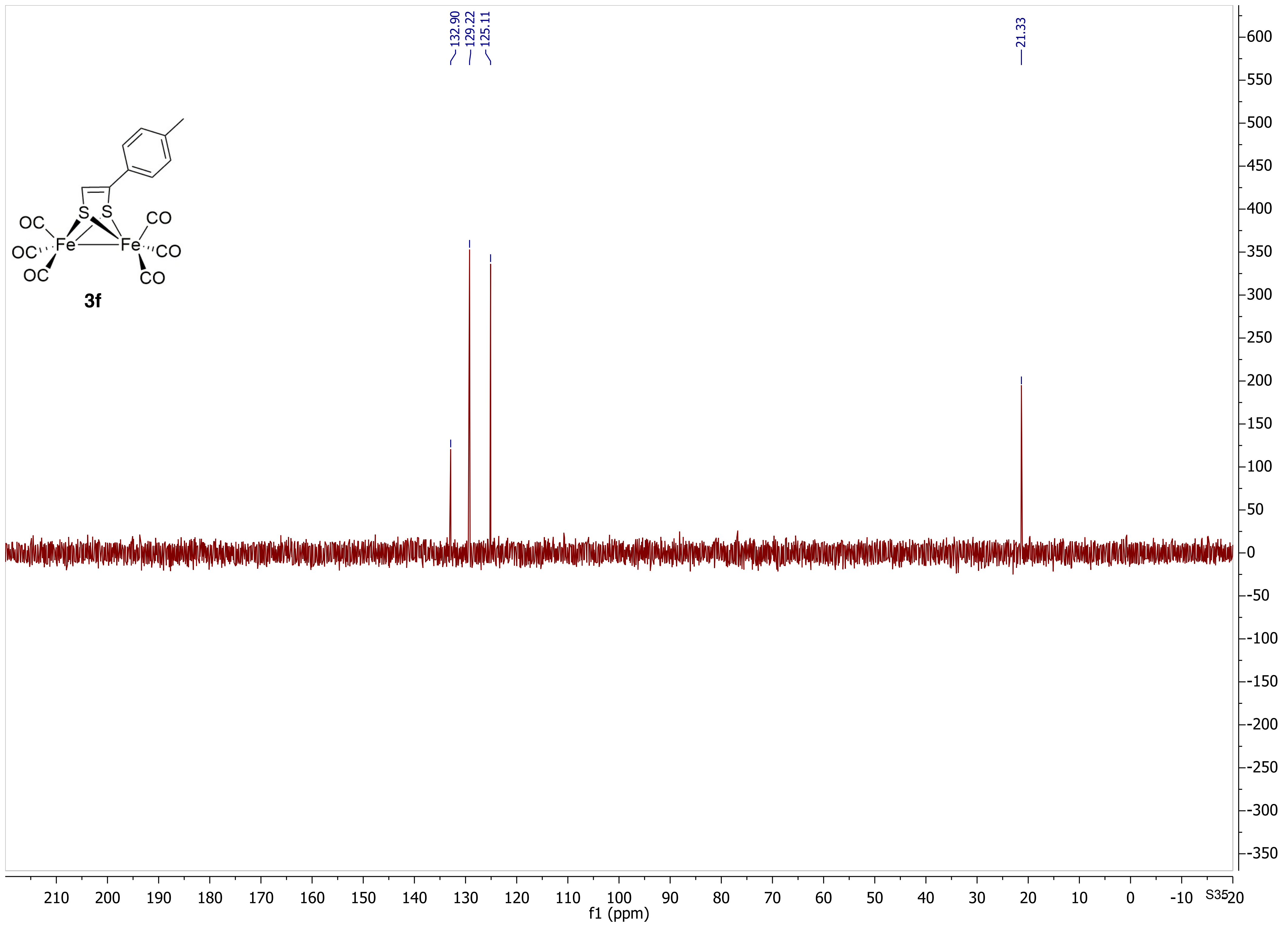
132.90
129.22
125.11
21.33
OC
OC
OC
Fe
Fe
S
S
CO
CO
CO
3f
f1 (ppm)

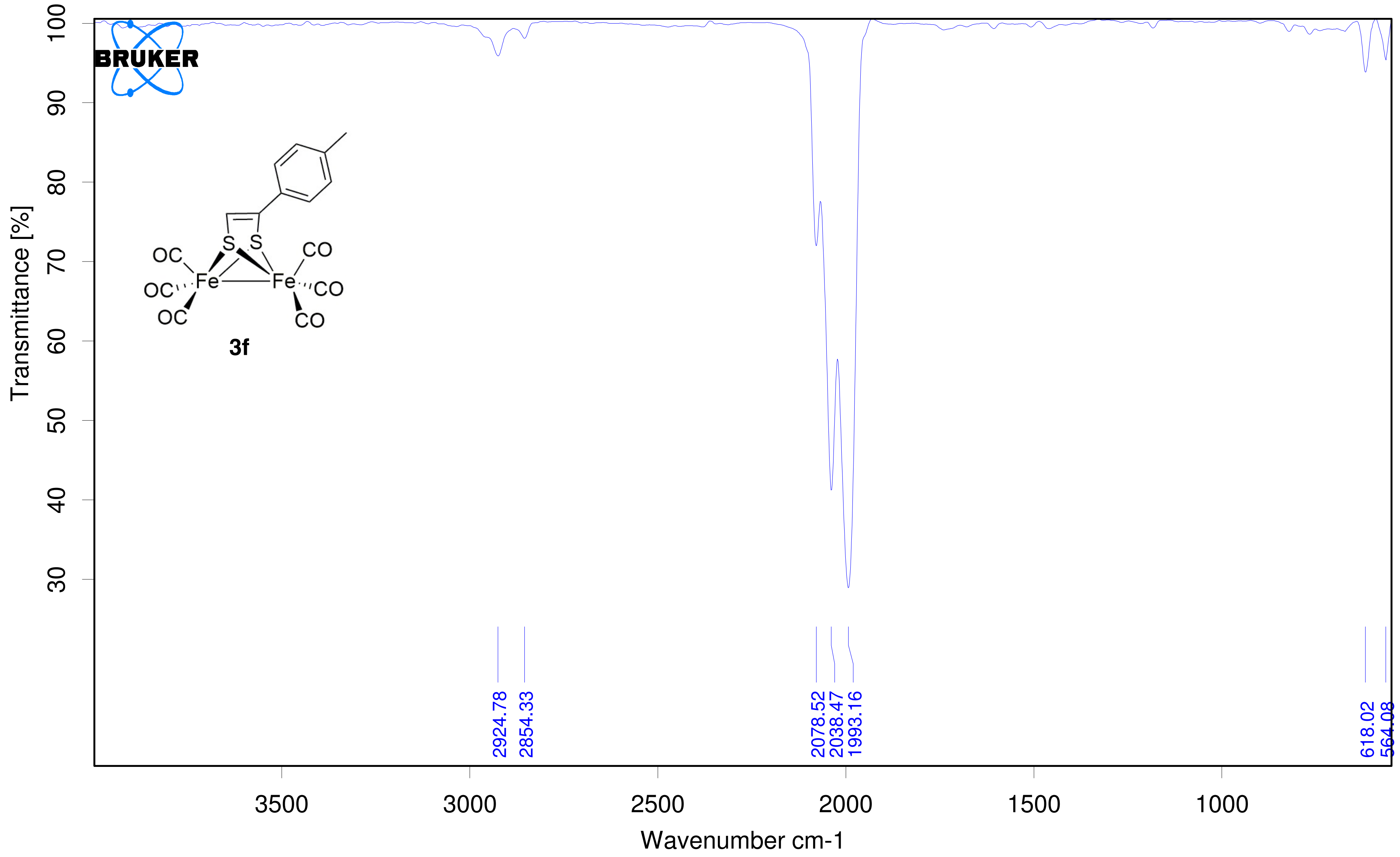
BRUKER
Transmittance [%]
Wavenumber cm-1
3f
2924.78
2854.33
2078.52
2038.47
1993.16
618.02
564.08

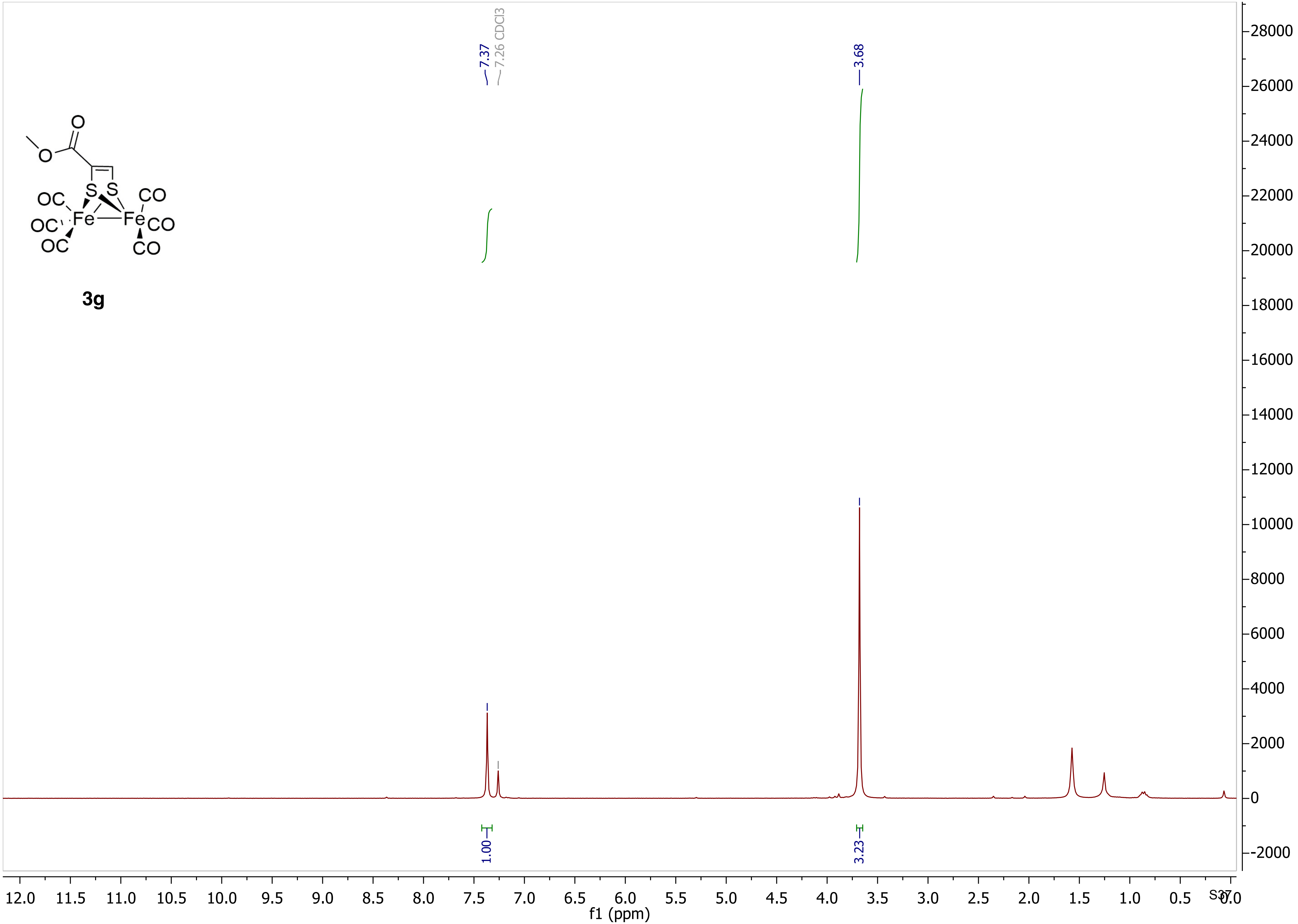
OC
OC
OC
CO
CO
CO
Fe
Fe
S
S
O
O
3g
7.37
7.26 CDCl3
3.68
1.00
3.23
f1 (ppm)

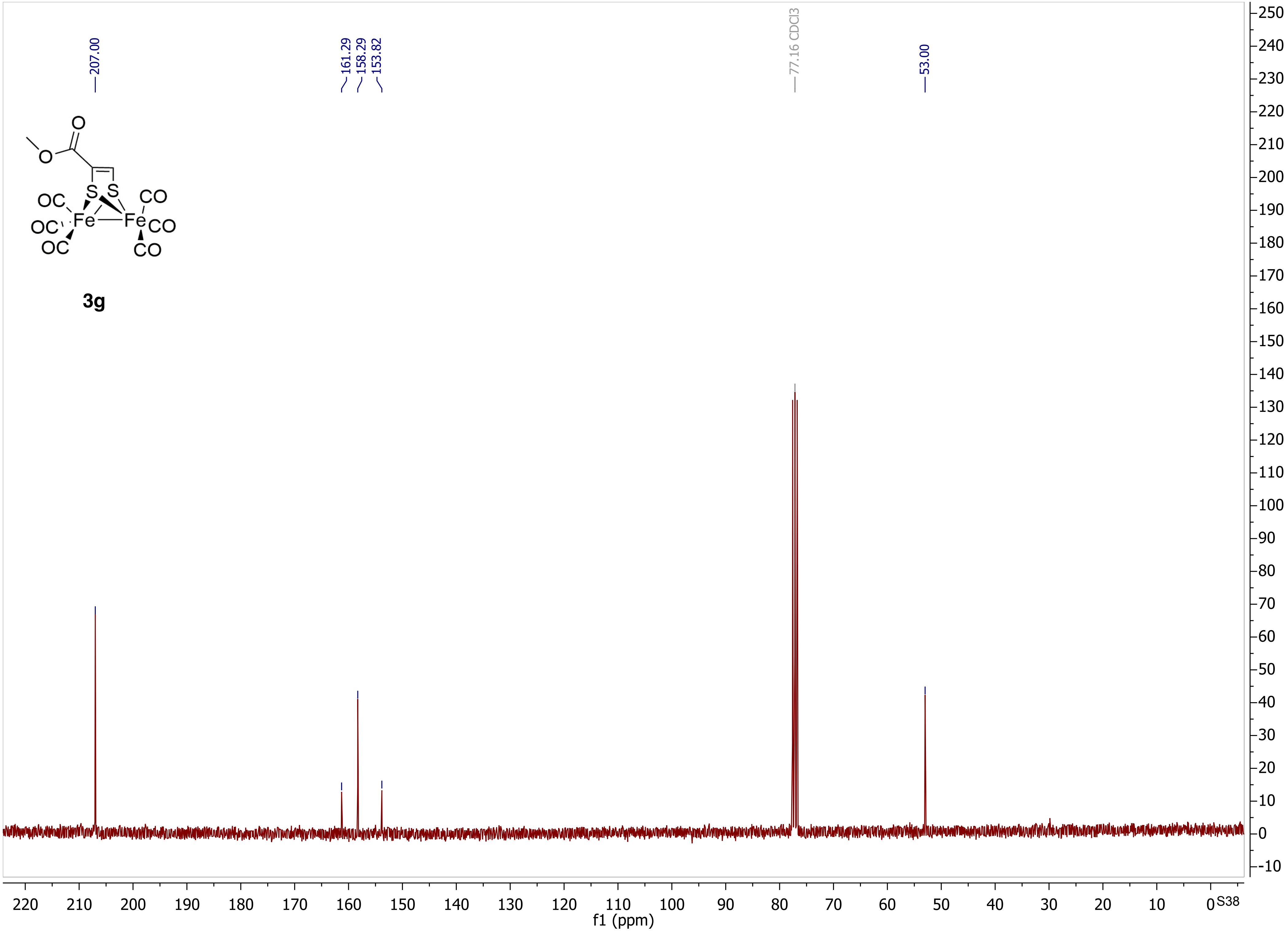
O
O
S
S
OC
CO
OC
Fe
Fe
CO
OC
CO
3g
207.00
161.29
158.29
153.82
77.16 CDCl3
53.00
f1 (ppm)

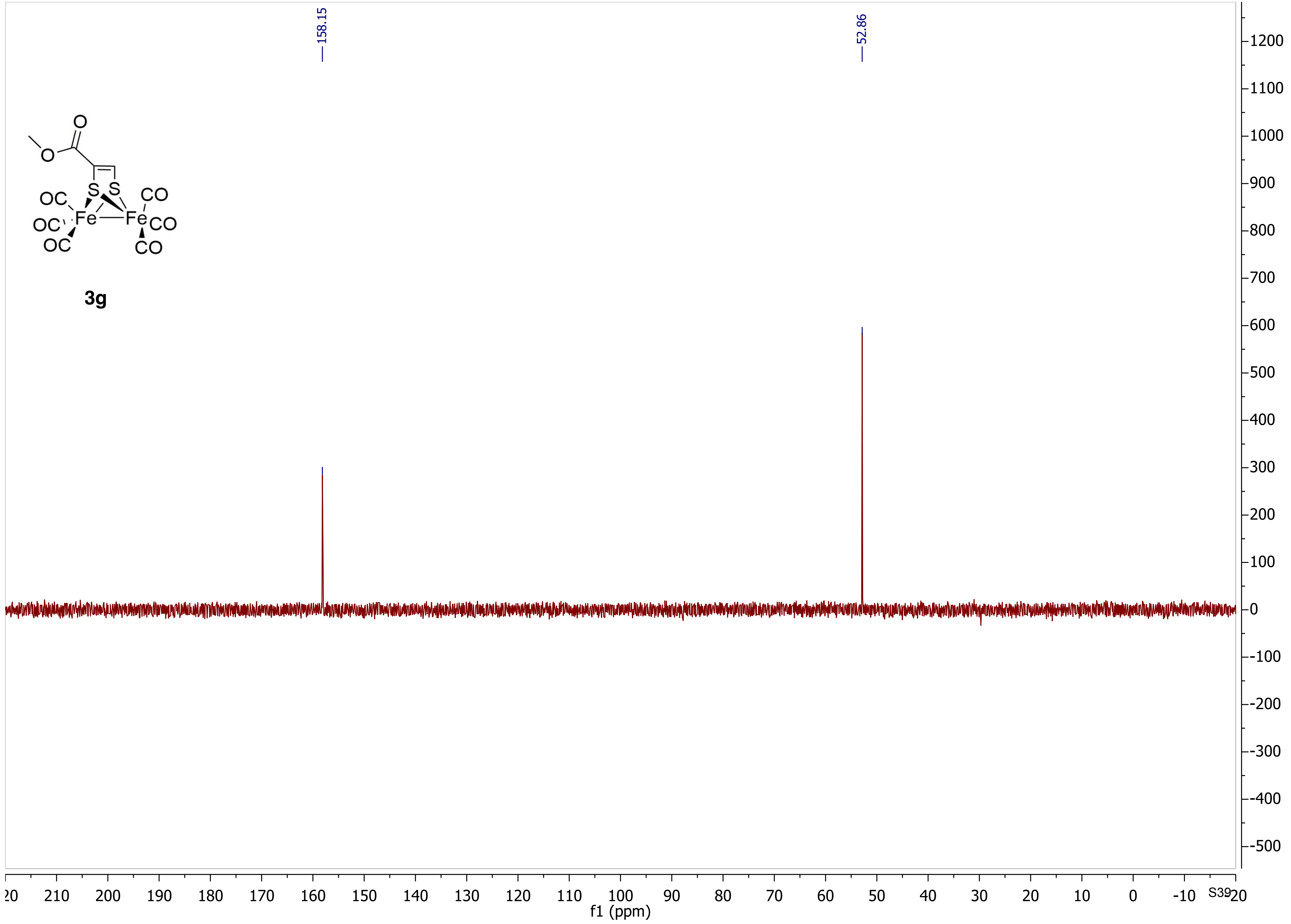

158.15
52.86
O
O
S
S
OC
CO
OC
Fe
Fe
CO
OC
CO
3g
f1 (ppm)

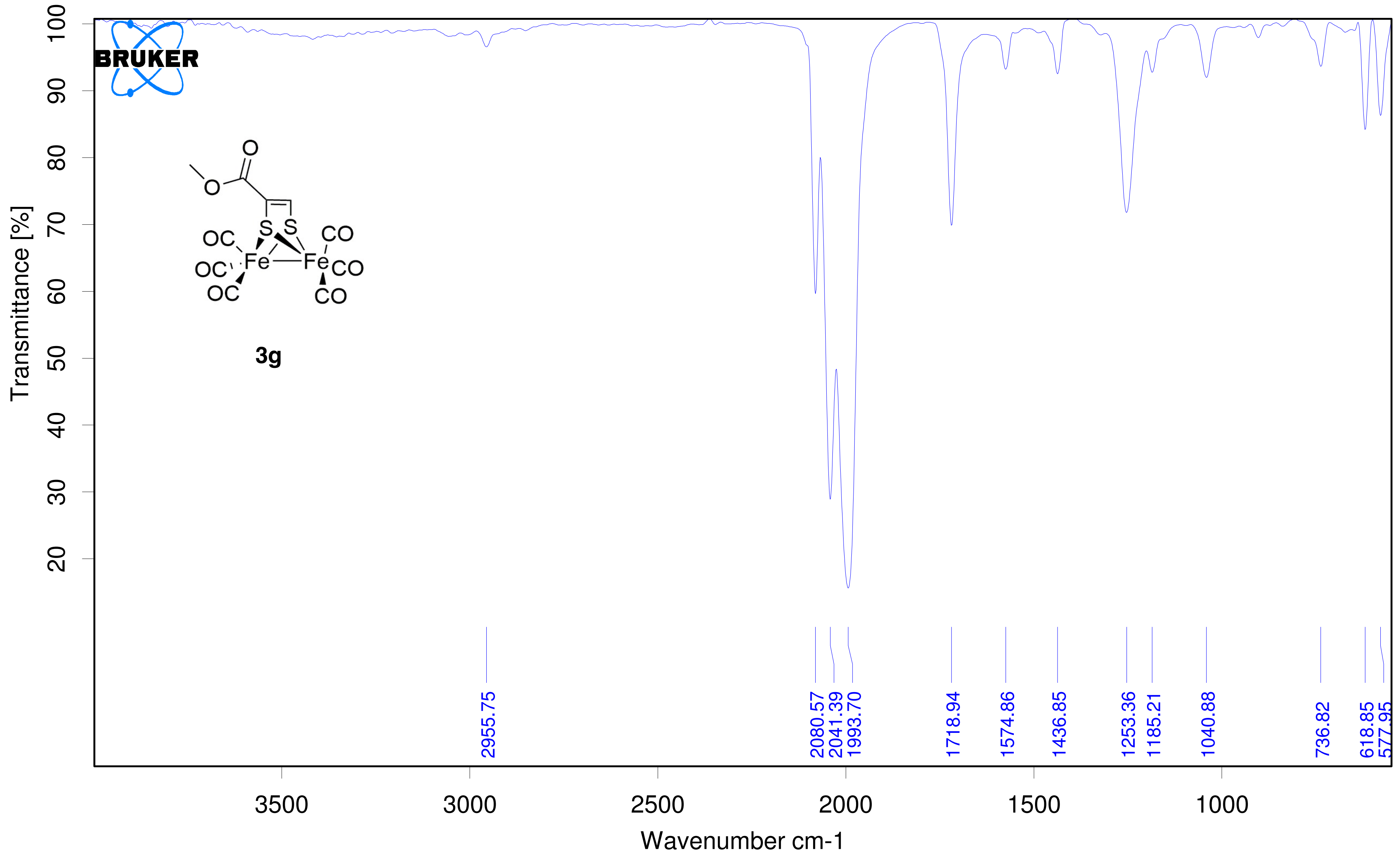

BRUKER
Transmittance [%]
100
90
80
70
60
50
40
30
20
3g
2955.75
2080.57
2041.39
1993.70
1718.94
1574.86
1436.85
1253.36
1185.21
1040.88
736.82
618.85
577.95
3500
3000
2500
2000
1500
1000
Wavenumber cm-1

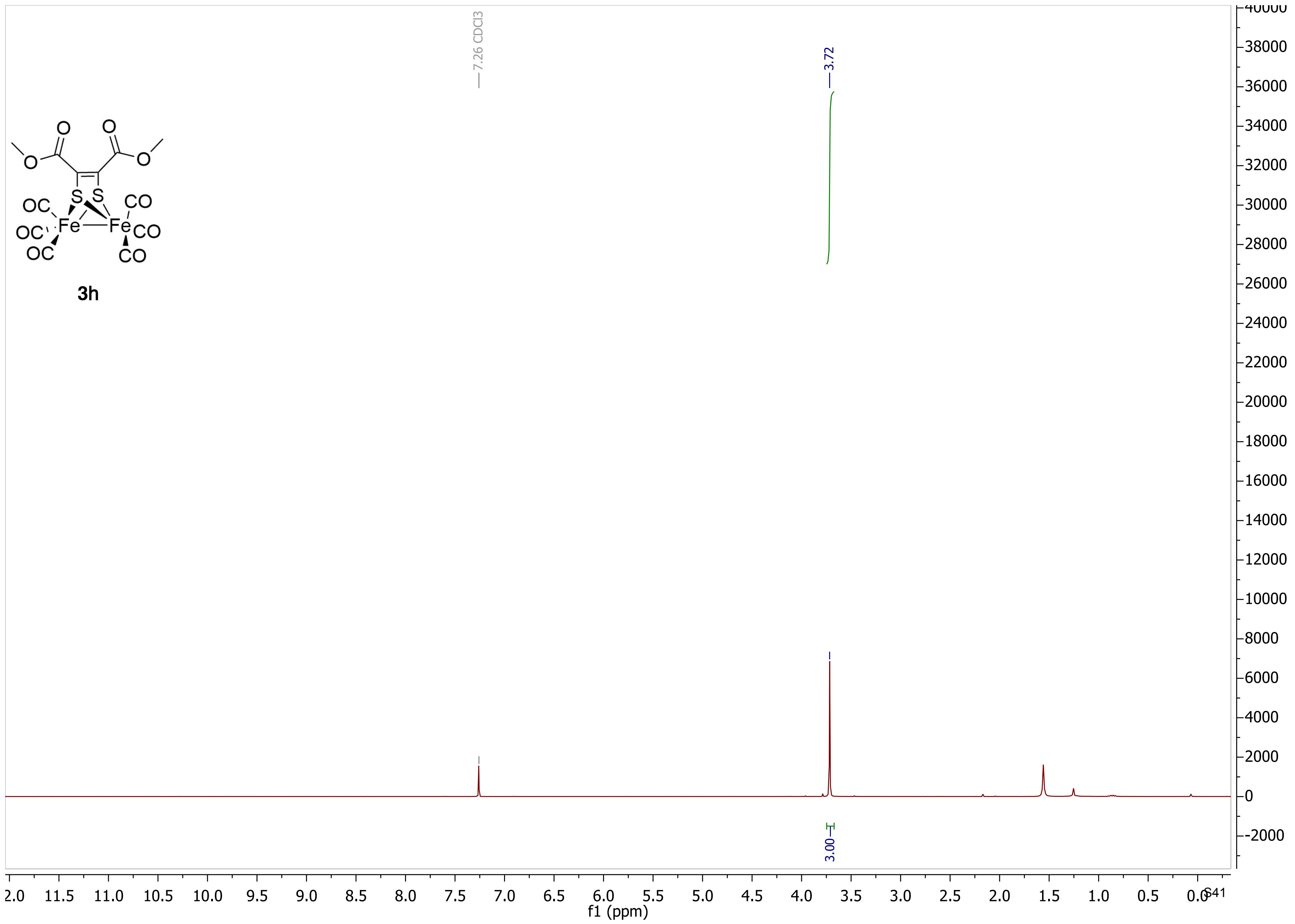
7.26 CDCl3
3.72
3h
OC
OC
OC
Fe
Fe
CO
CO
CO
S
S
O
O
O
O
3.00
f1 (ppm)
2.0
11.5
11.0
10.5
10.0
9.5
9.0
8.5
8.0
7.5
7.0
6.5
6.0
5.5
5.0
4.5
4.0
3.5
3.0
2.5
2.0
1.5
1.0
0.5
0.0
40000
38000
36000
34000
32000
30000
28000
26000
24000
22000
20000
18000
16000
14000
12000
10000
8000
6000
4000
2000
0
-2000

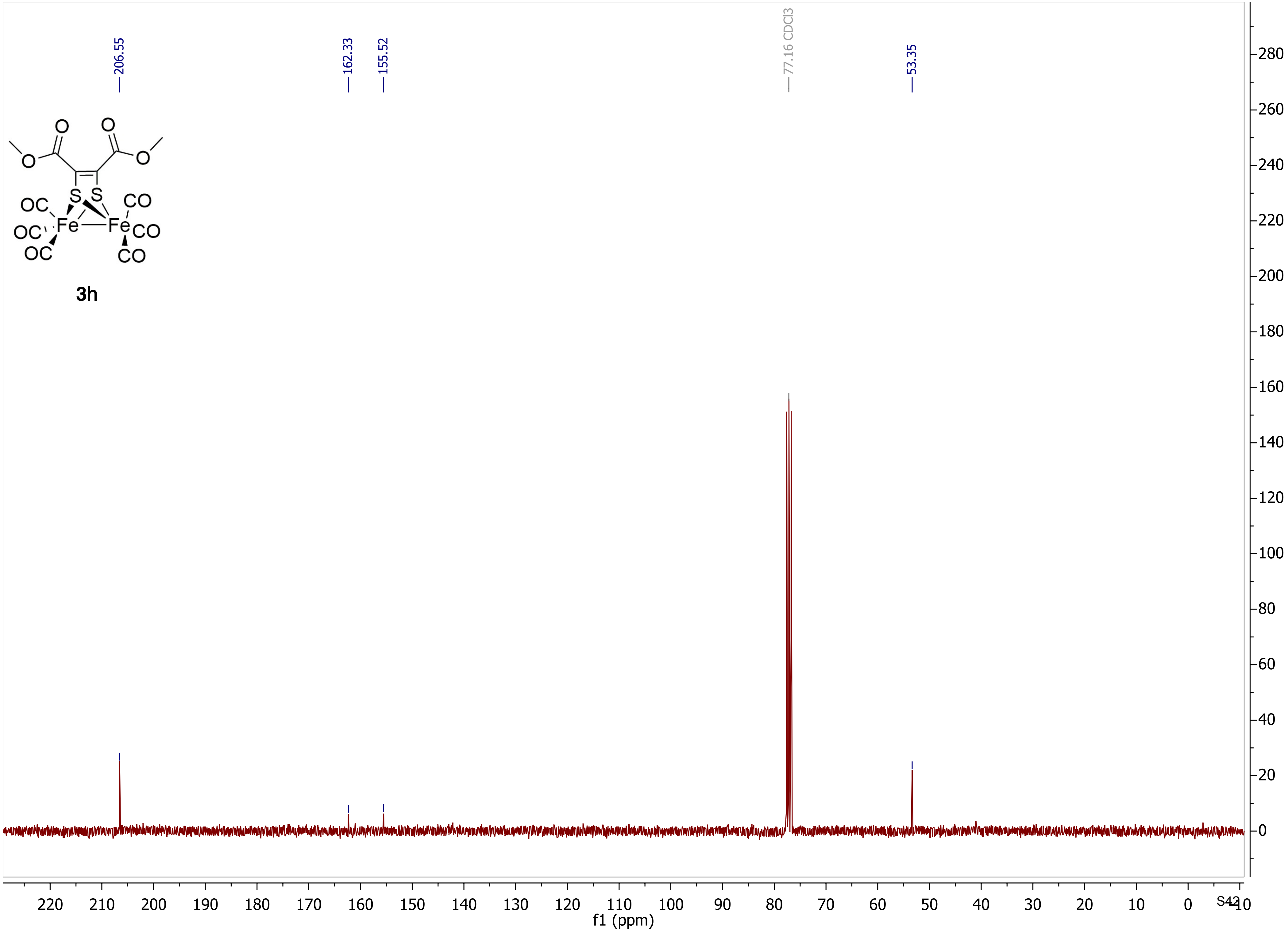
206.55
162.33
155.52
77.16 CDCl3
53.35
3h
f1 (ppm)

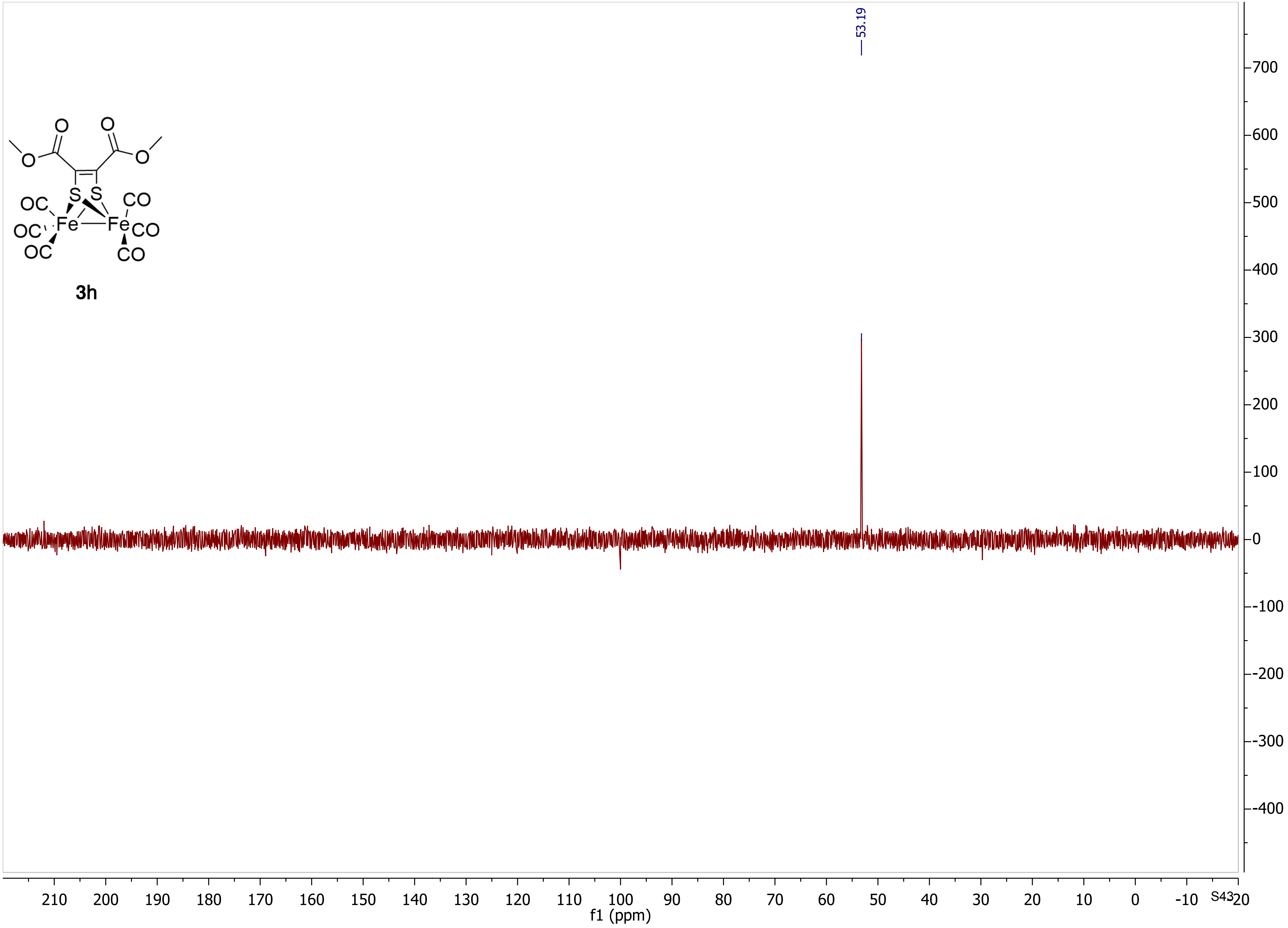

53.19
3h
f1 (ppm)

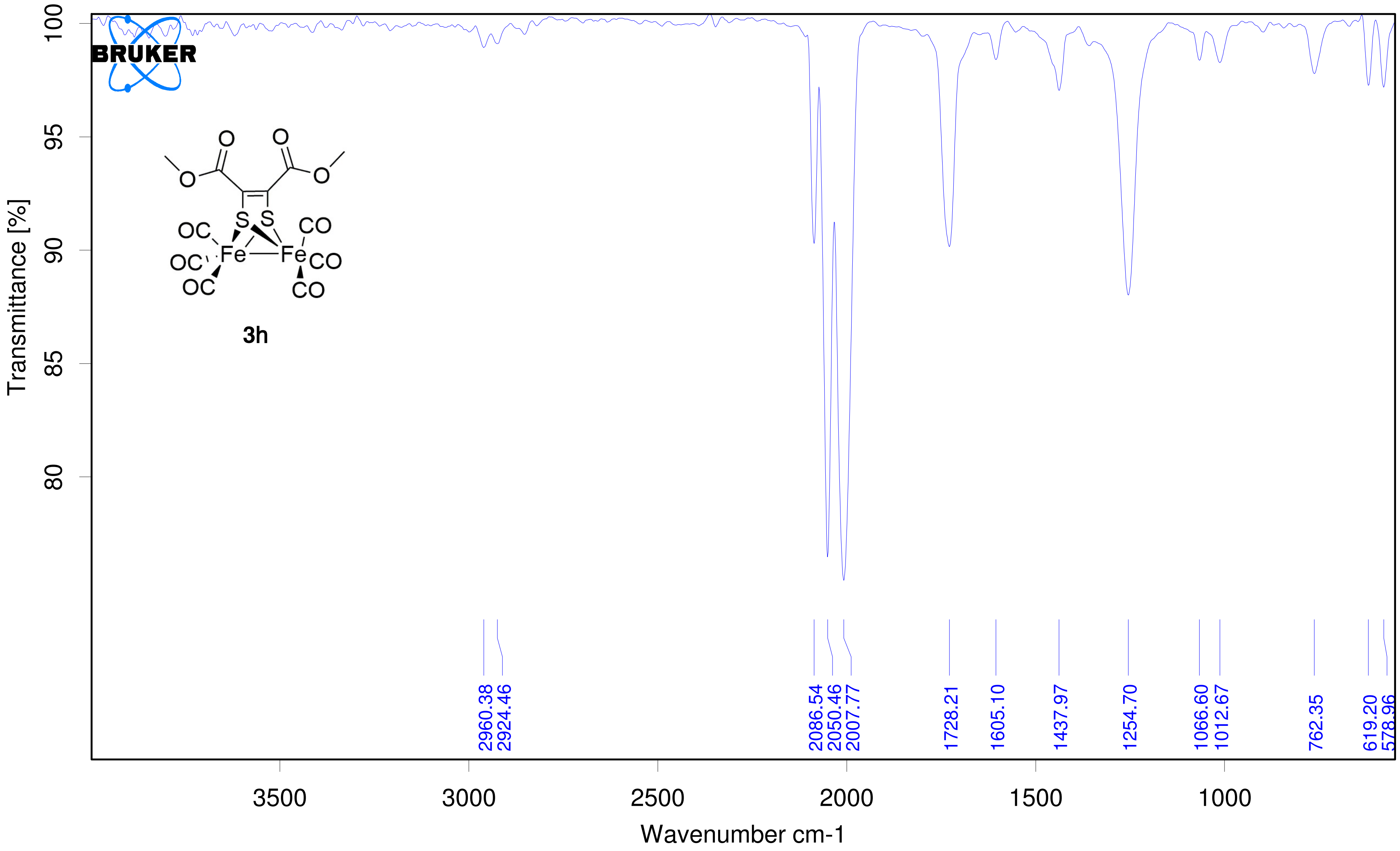

BRUKER
3h
Transmittance [%]
100
95
90
85
80
Wavenumber cm-1
3500
3000
2500
2000
1500
1000
2960.38
2924.46
2086.54
2050.46
2007.77
1728.21
1605.10
1437.97
1254.70
1066.60
1012.67
762.35
619.20
578.96

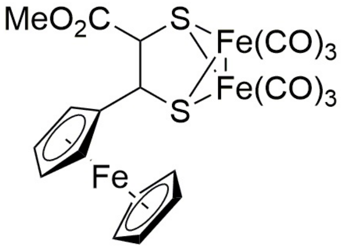

MeO2C
S
Fe(CO)3
Fe(CO)3
S
Fe

3i

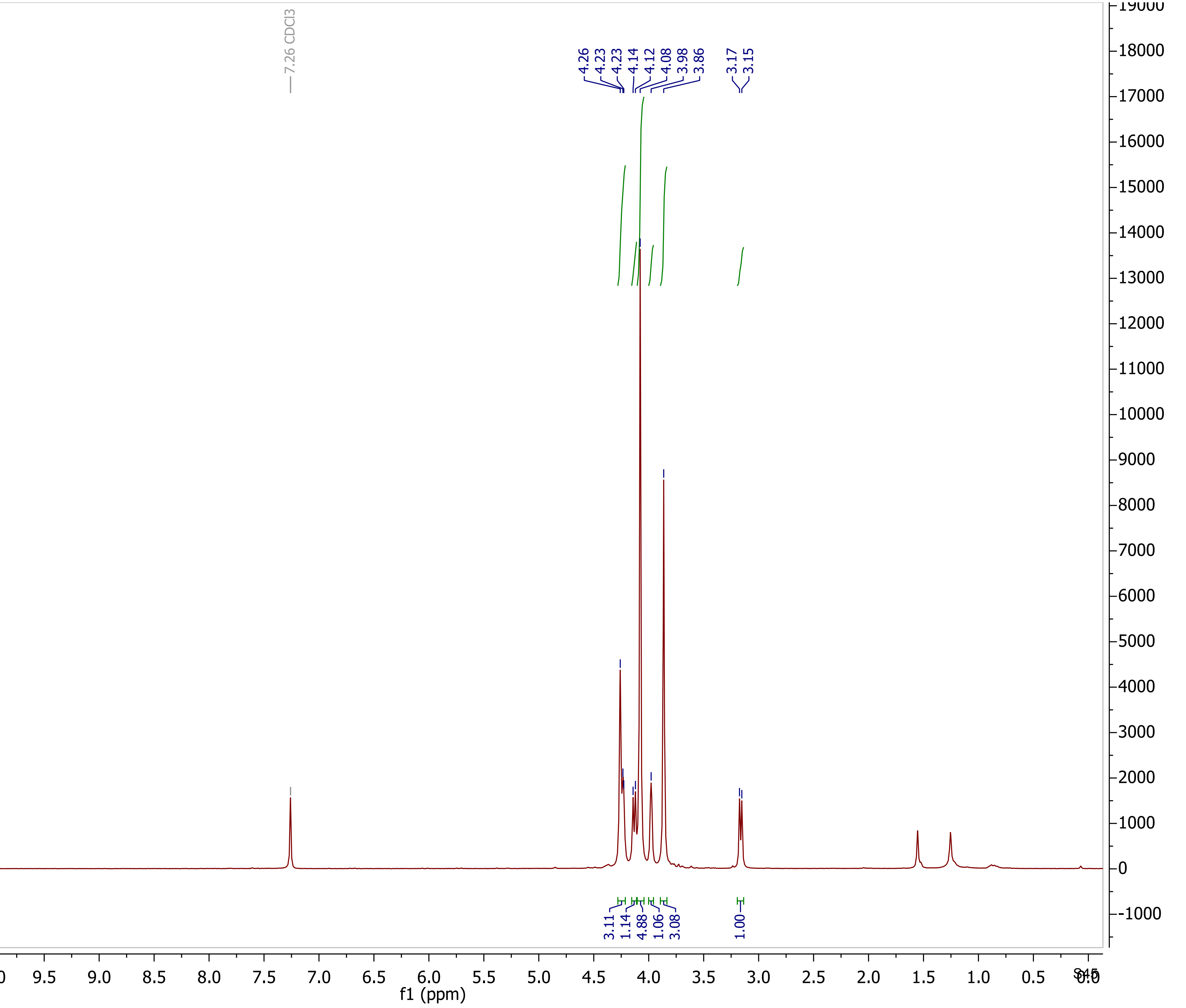

7.26 CDCl3
4.26
4.23
4.23
4.14
4.12
4.08
3.98
3.86
3.17
3.15
3.11
1.14
4.88
1.06
3.08
1.00
f1 (ppm)

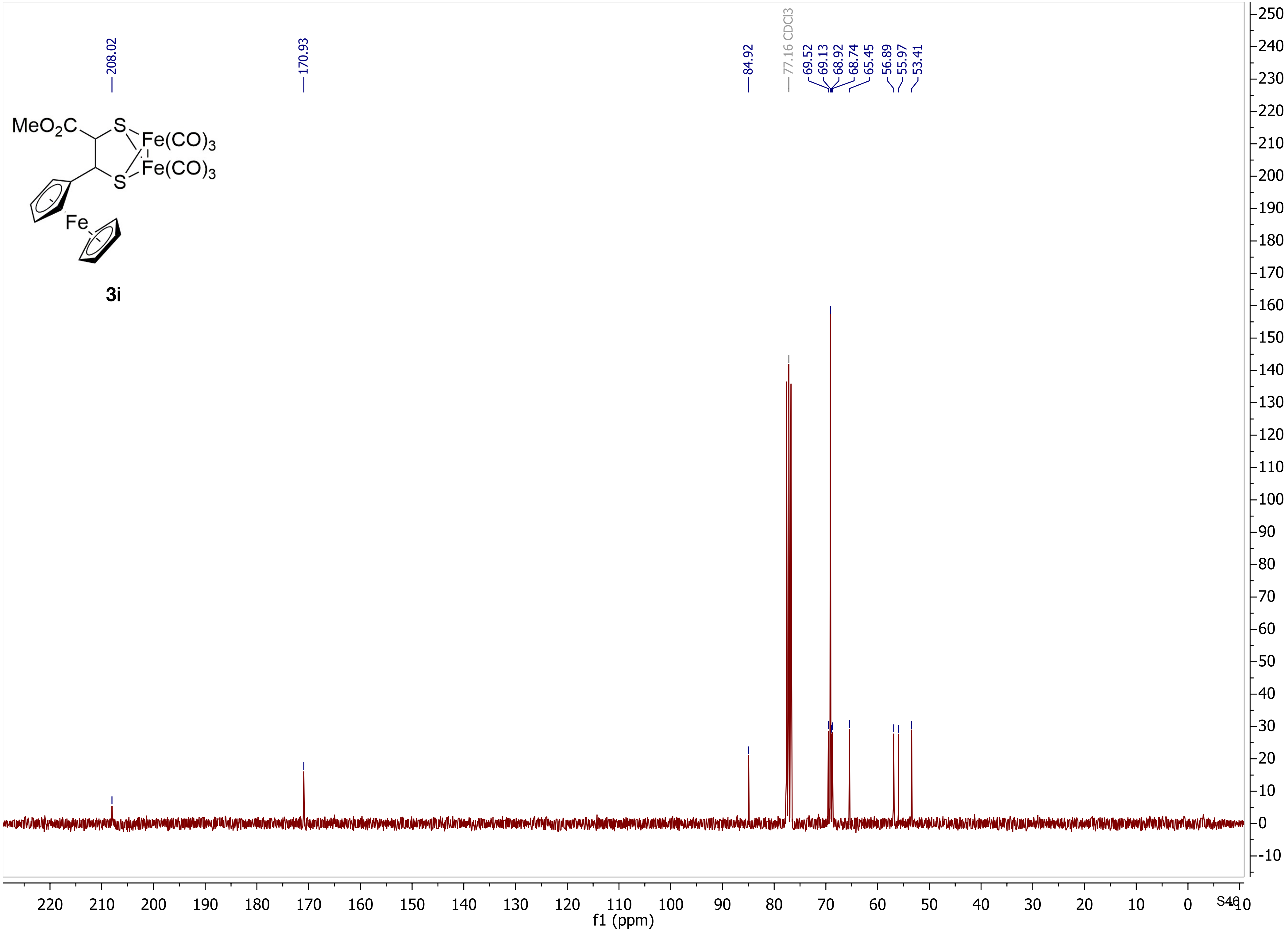

208.02
170.93
84.92
77.16 CDCl3
69.52
69.13
68.92
68.74
65.45
56.89
55.97
53.41
MeO2C
S
Fe(CO)3
Fe(CO)3
S
Fe
3i
f1 (ppm)

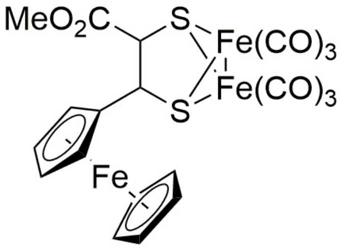

3i

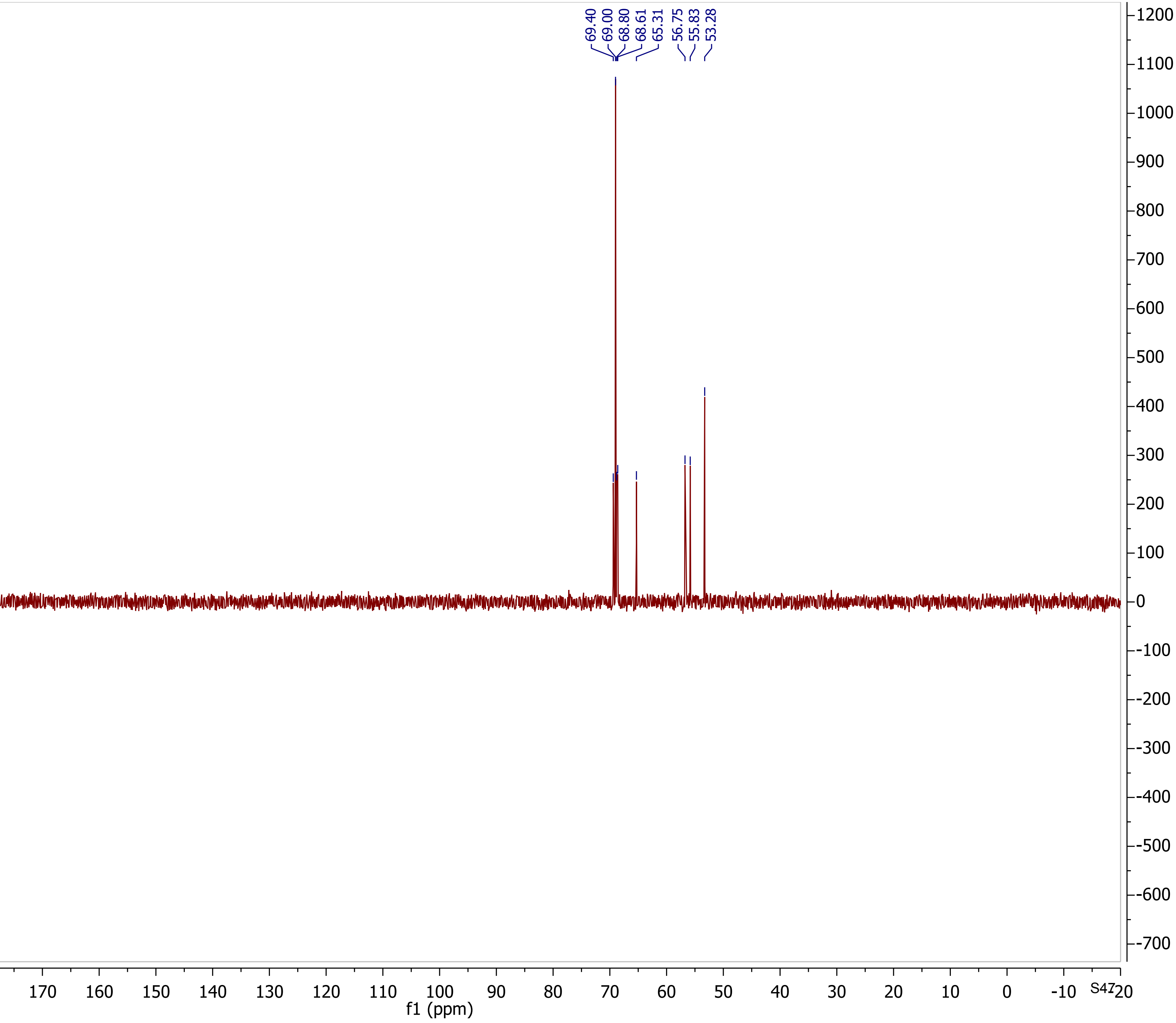

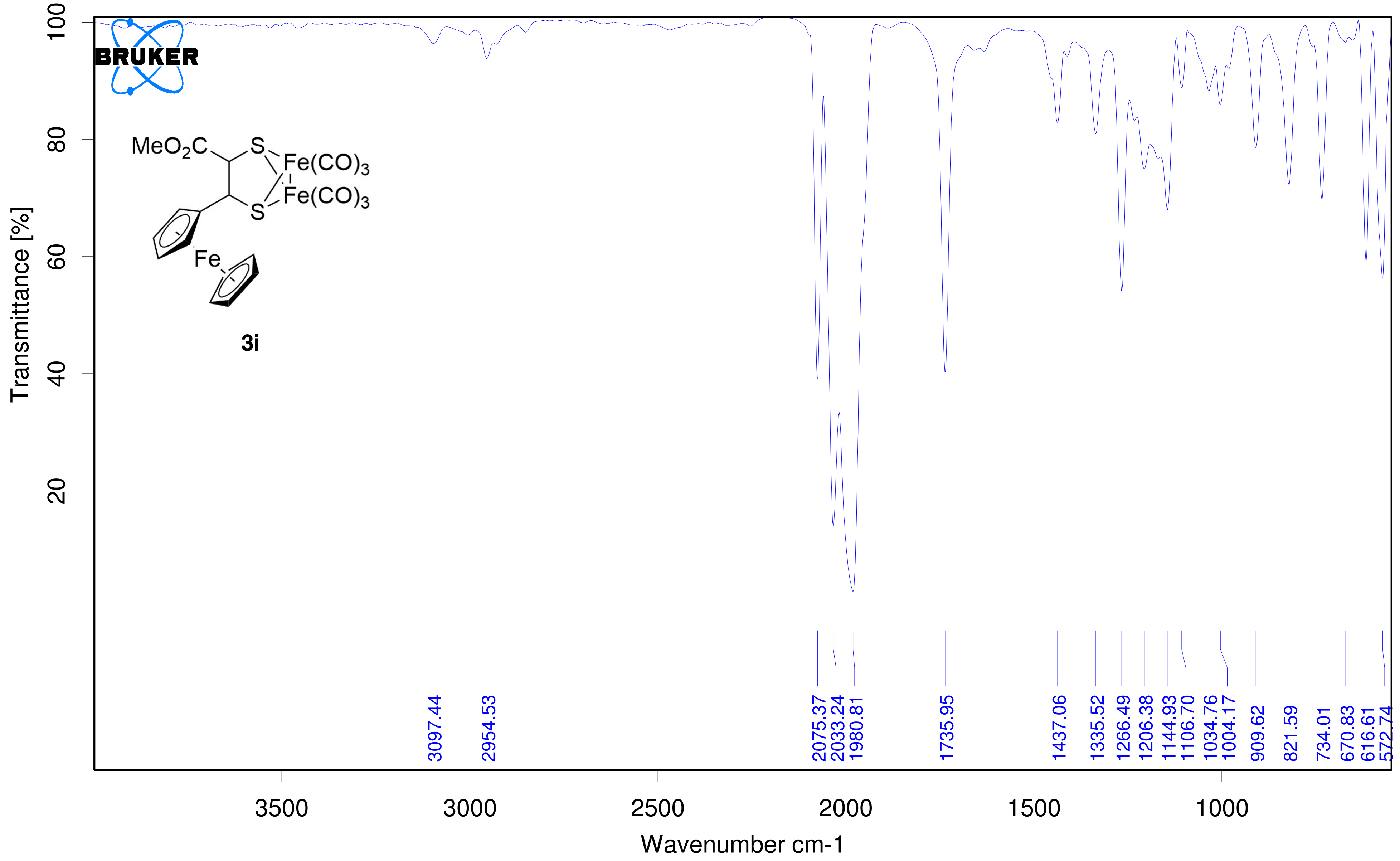
MeO2C
S
Fe(CO)3
Fe(CO)3
S
Fe
3i
Transmittance [%]
Wavenumber cm-1
3097.44
2954.53
2075.37
2033.24
1980.81
1735.95
1437.06
1335.52
1266.49
1206.38
1144.93
1106.70
1034.76
1004.17
909.62
821.59
734.01
670.83
616.61
572.74

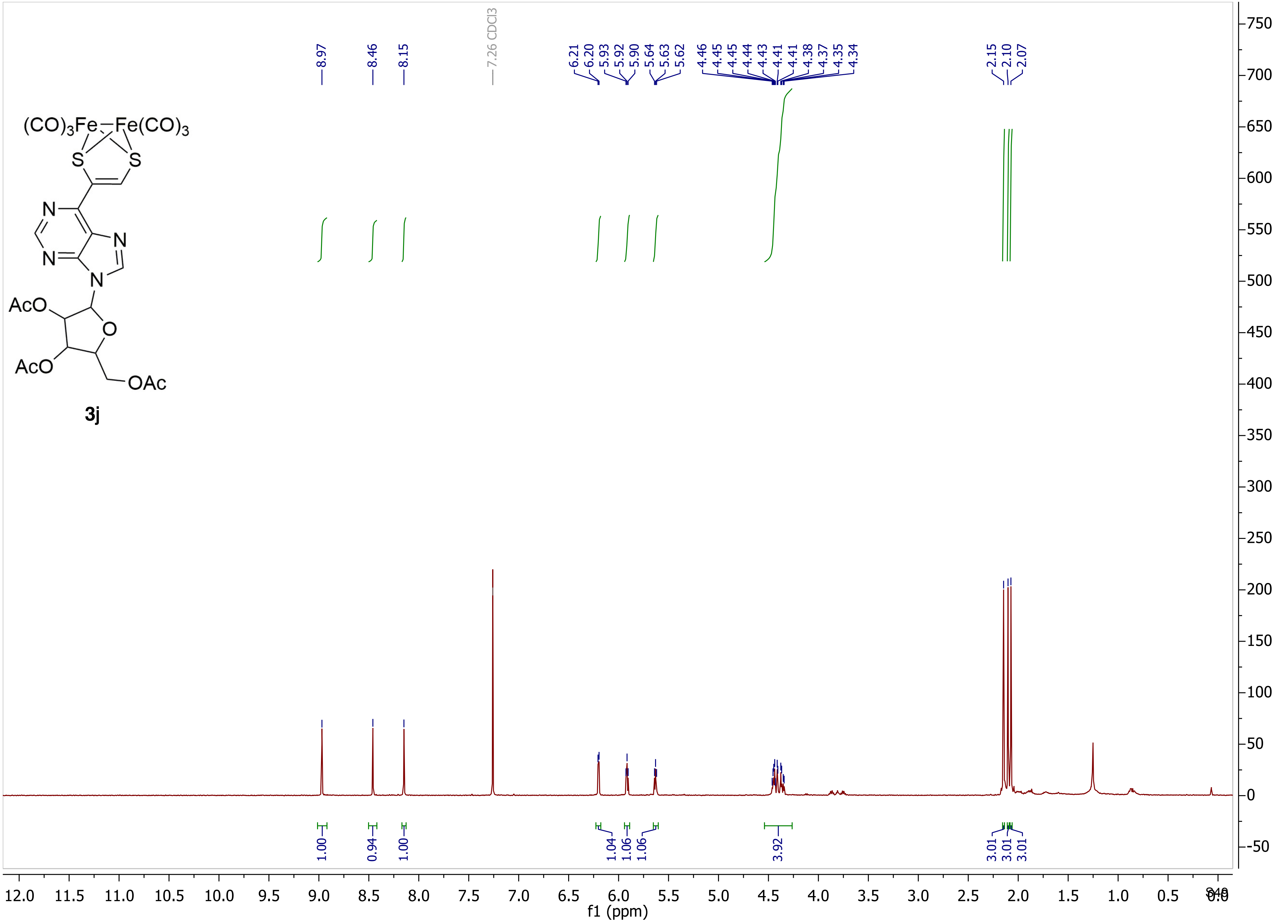
(CO)3Fe—Fe(CO)3
S S
N N N N
AcO AcO OAc O
3j
8.97
8.46
8.15
7.26 CDCl3
6.21 6.20 5.93 5.92 5.90 5.64 5.63 5.62
4.46 4.45 4.45 4.44 4.43 4.41 4.41 4.38 4.37 4.35 4.34
2.15 2.10 2.07
1.00 0.94 1.00 1.04 1.06 1.06 3.92 3.01 3.01 3.01
12.0 11.5 11.0 10.5 10.0 9.5 9.0 8.5 8.0 7.5 7.0 6.5 6.0 5.5 5.0 4.5 4.0 3.5 3.0 2.5 2.0 1.5 1.0 0.5 0.0
f1 (ppm)
750 700 650 600 550 500 450 400 350 300 250 200 150 100 50 0 -50

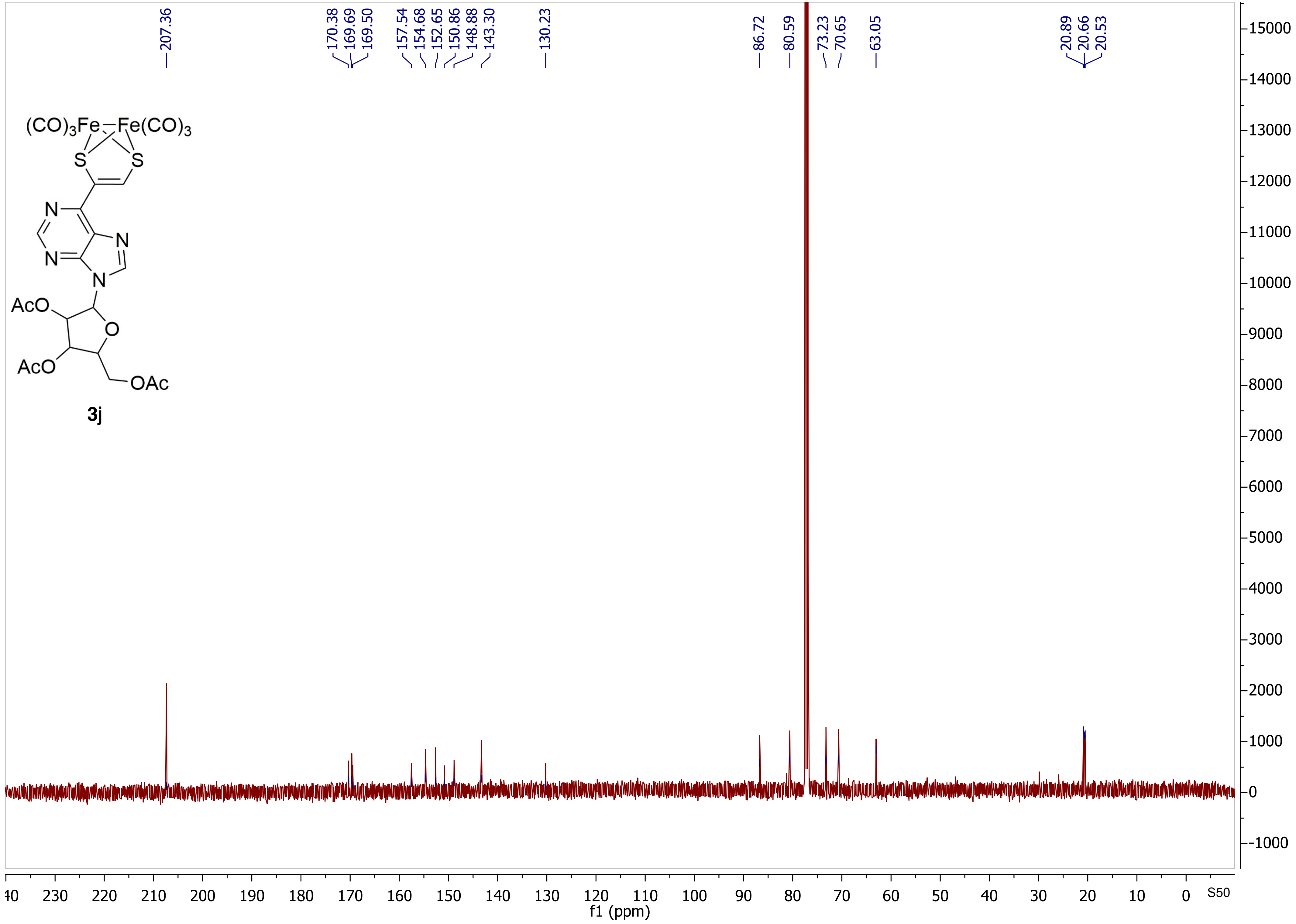

207.36
170.38
169.69
169.50
157.54
154.68
152.65
150.86
148.88
143.30
130.23
86.72
80.59
73.23
70.65
63.05
20.89
20.66
20.53
(CO)3Fe–Fe(CO)3
S
S
N
N
N
N
AcO
AcO
O
OAc
3j
f1 (ppm)

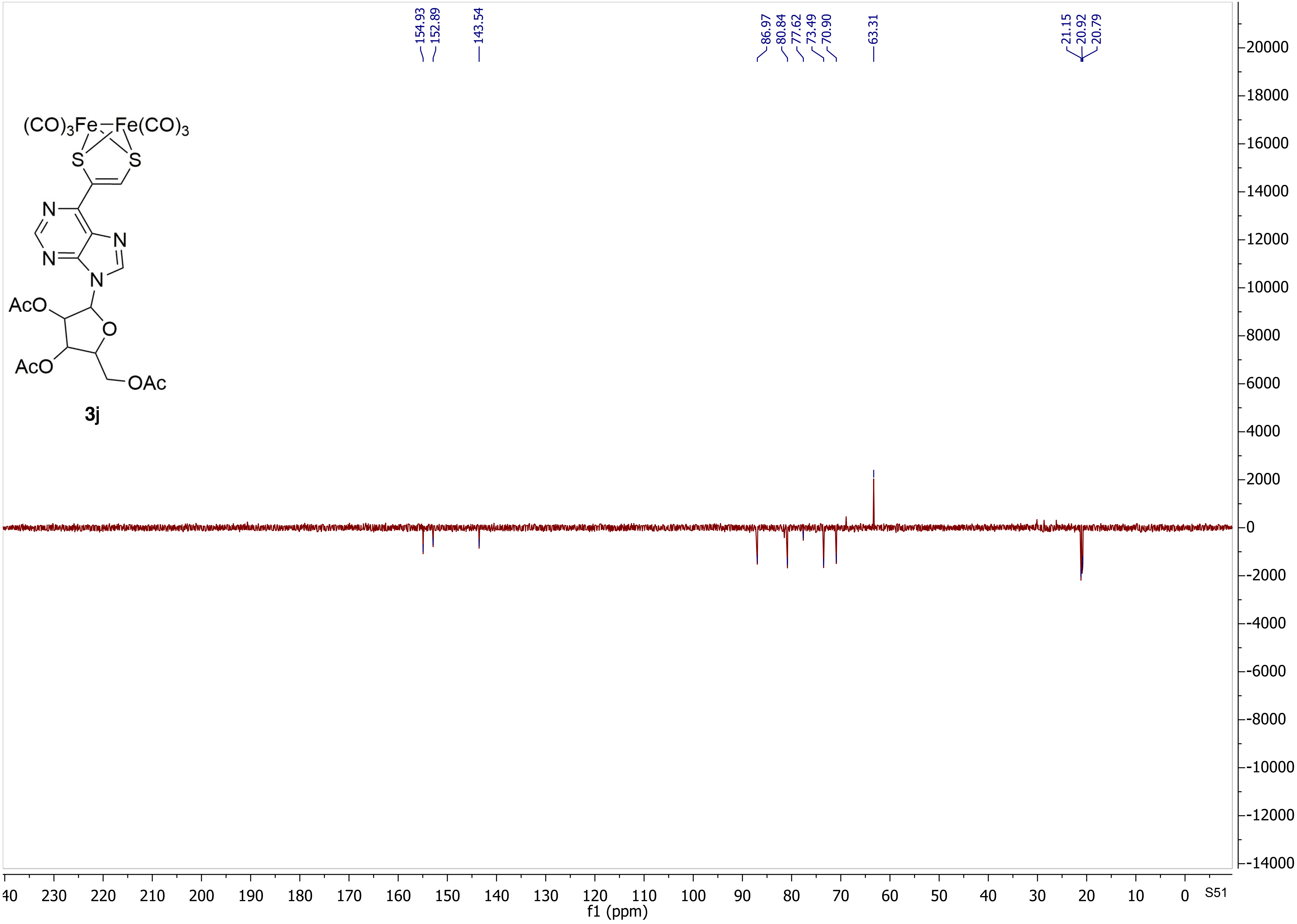
154.93
152.89
143.54
86.97
80.84
77.62
73.49
70.90
63.31
21.15
20.92
20.79
(CO)3Fe—Fe(CO)3
S
S
N
N
N
N
AcO
O
AcO
OAc
3j
20000
18000
16000
14000
12000
10000
8000
6000
4000
2000
0
-2000
-4000
-6000
-8000
-10000
-12000
-14000
40 230 220 210 200 190 180 170 160 150 140 130 120 110 100 90 80 70 60 50 40 30 20 10 0
f1 (ppm)

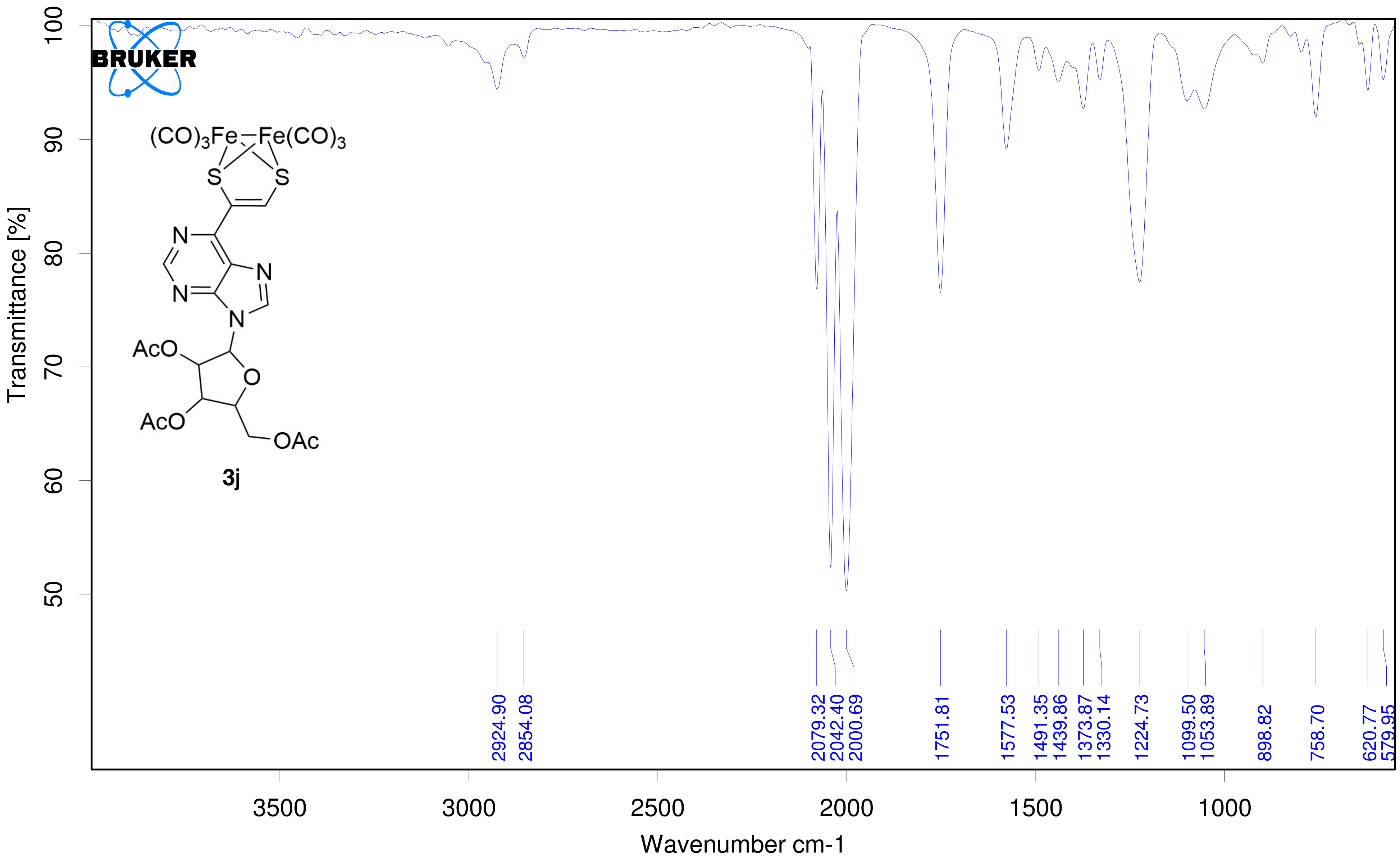

BRUKER
(CO)3Fe–Fe(CO)3
S
S
N
N
N
N
AcO
O
AcO
OAc
3j
Transmittance [%]
100
90
80
70
60
50
Wavenumber cm-1
3500
3000
2500
2000
1500
1000
2924.90
2854.08
2079.32
2042.40
2000.69
1751.81
1577.53
1491.35
1439.86
1373.87
1330.14
1224.73
1099.50
1053.89
898.82
758.70
620.77
579.95

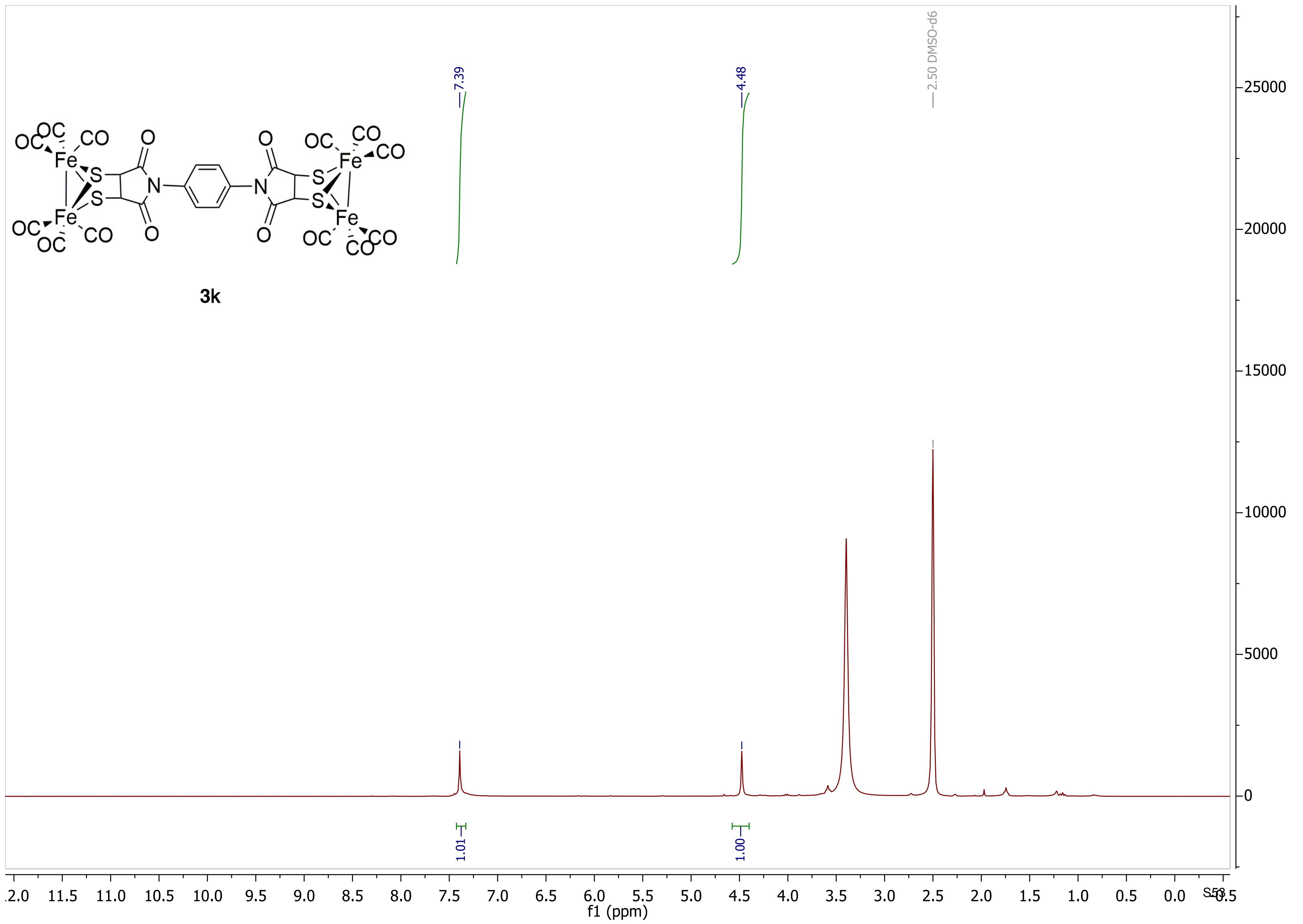
3k
7.39
4.48
2.50 DMSO-d6
1.01
1.00
f1 (ppm)

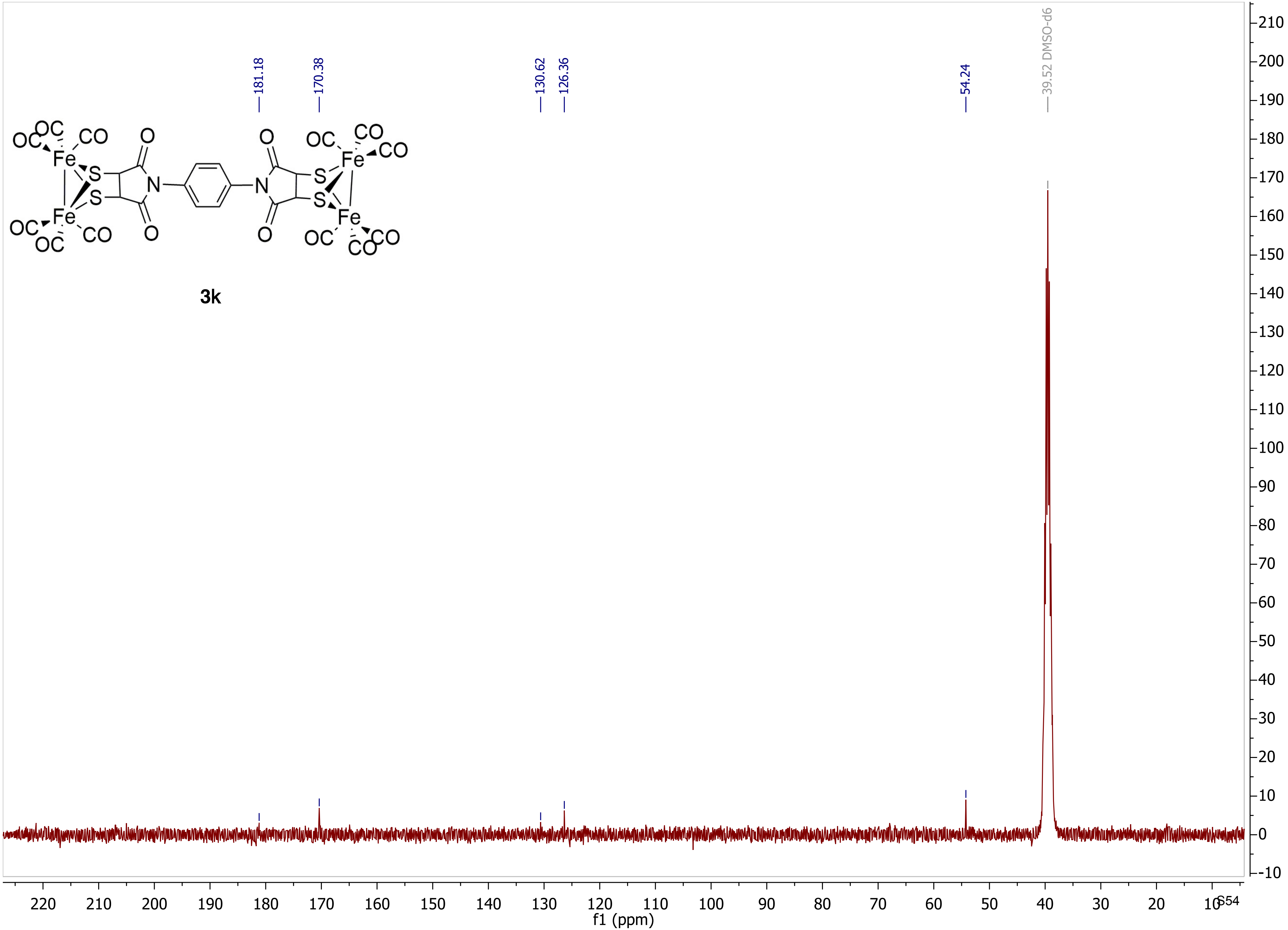

181.18
170.38
130.62
126.36
54.24
39.52 DMSO-d6
220
210
200
190
180
170
160
150
140
130
120
110
100
90
80
70
60
50
40
30
20
10
f1 (ppm)

3k

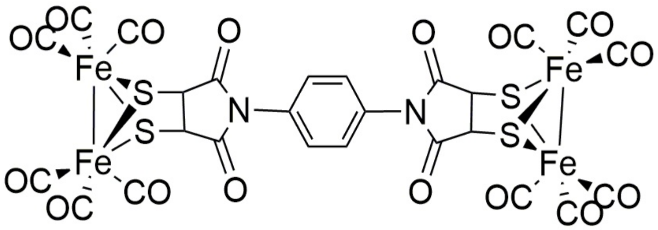

3k

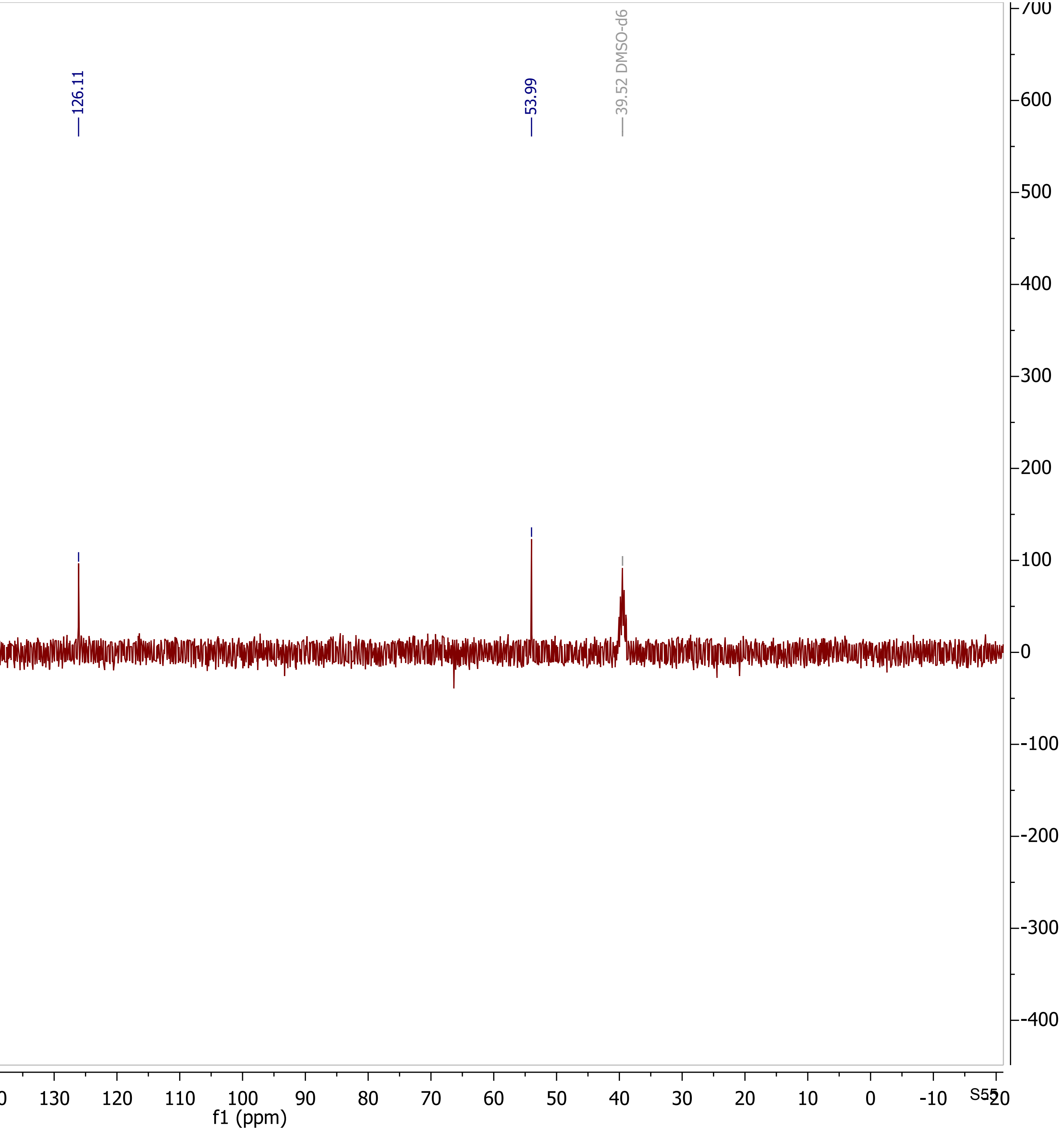

126.11
53.99
39.52 DMSO-d6
700
600
500
400
300
200
100
0
-100
-200
-300
-400
210
200
190
180
170
160
150
140
130
120
110
100
90
80
70
60
50
40
30
20
10
0
-10
-20
f1 (ppm)

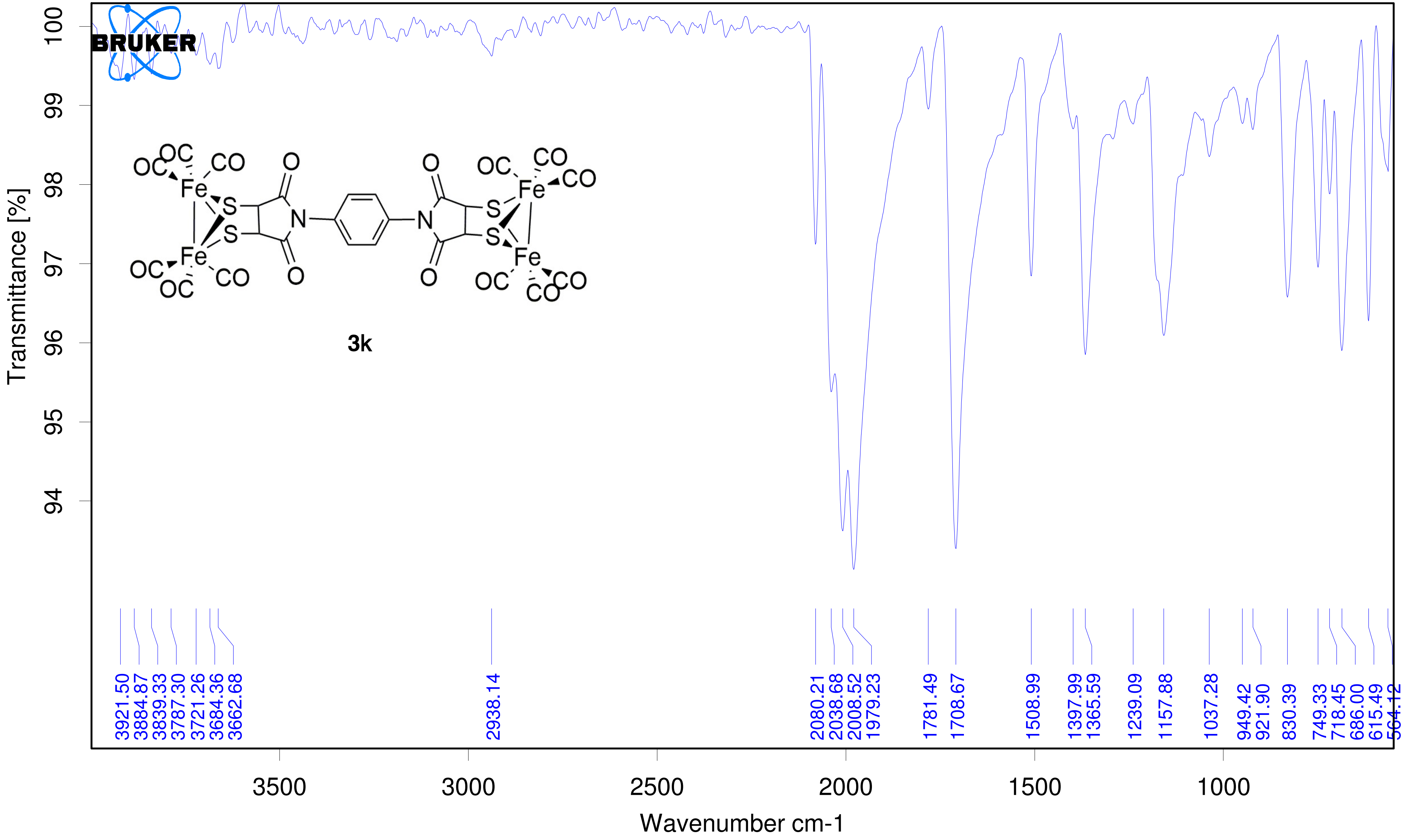
BRUKER
Transmittance [%]
Wavenumber cm-1
3k
3921.50
3884.87
3839.33
3787.30
3721.26
3684.36
3662.68
2938.14
2080.21
2038.68
2008.52
1979.23
1781.49
1708.67
1508.99
1397.99
1365.59
1239.09
1157.88
1037.28
949.42
921.90
830.39
749.33
718.45
686.00
615.49
564.12

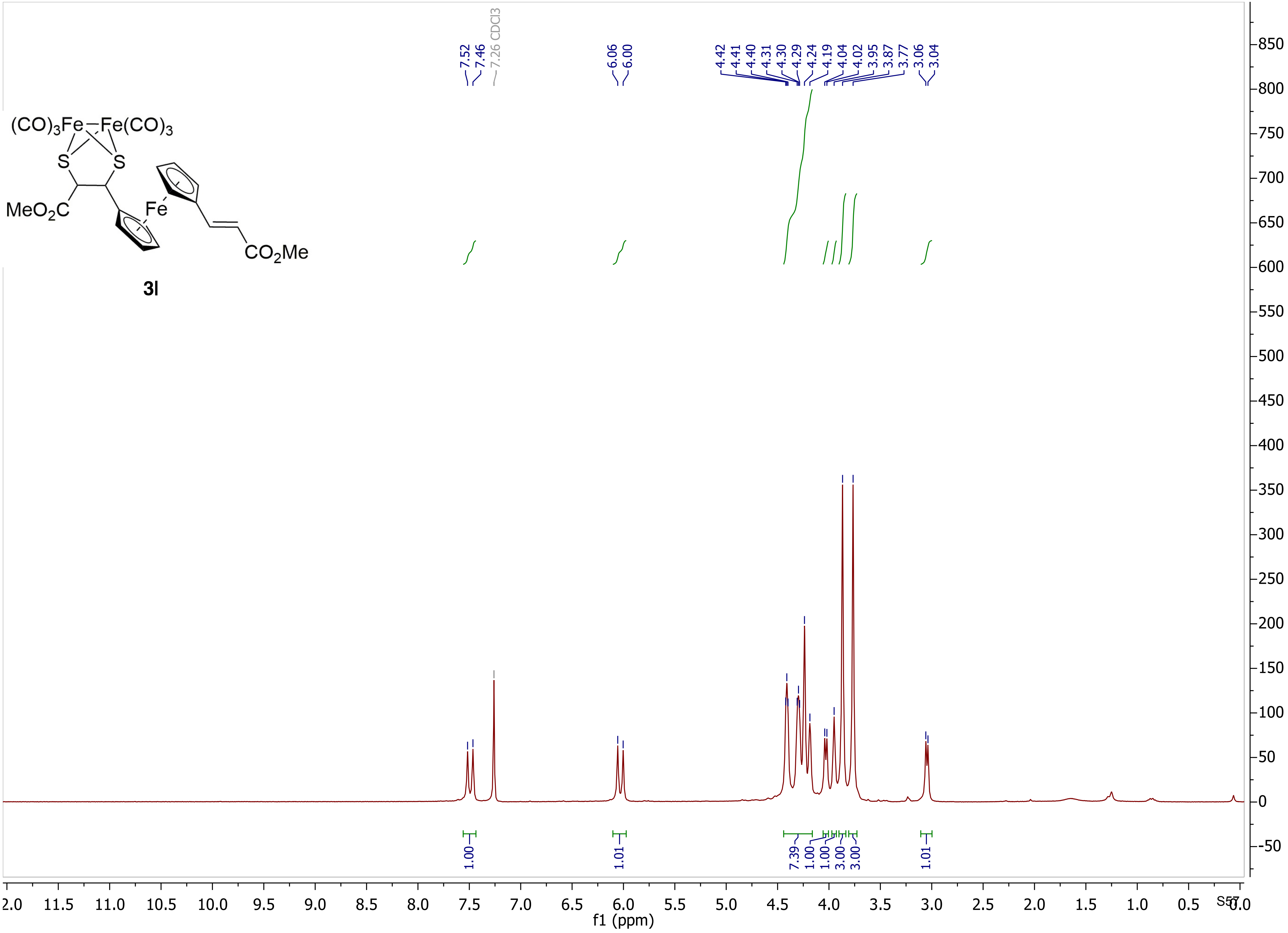

3l

7.52
7.46
7.26 CDCl3
6.06
6.00
4.42
4.41
4.40
4.31
4.30
4.29
4.24
4.19
4.04
4.02
3.95
3.87
3.77
3.06
3.04
1.00
1.01
7.39
1.00
1.00
3.00
3.00
1.01
f1 (ppm)

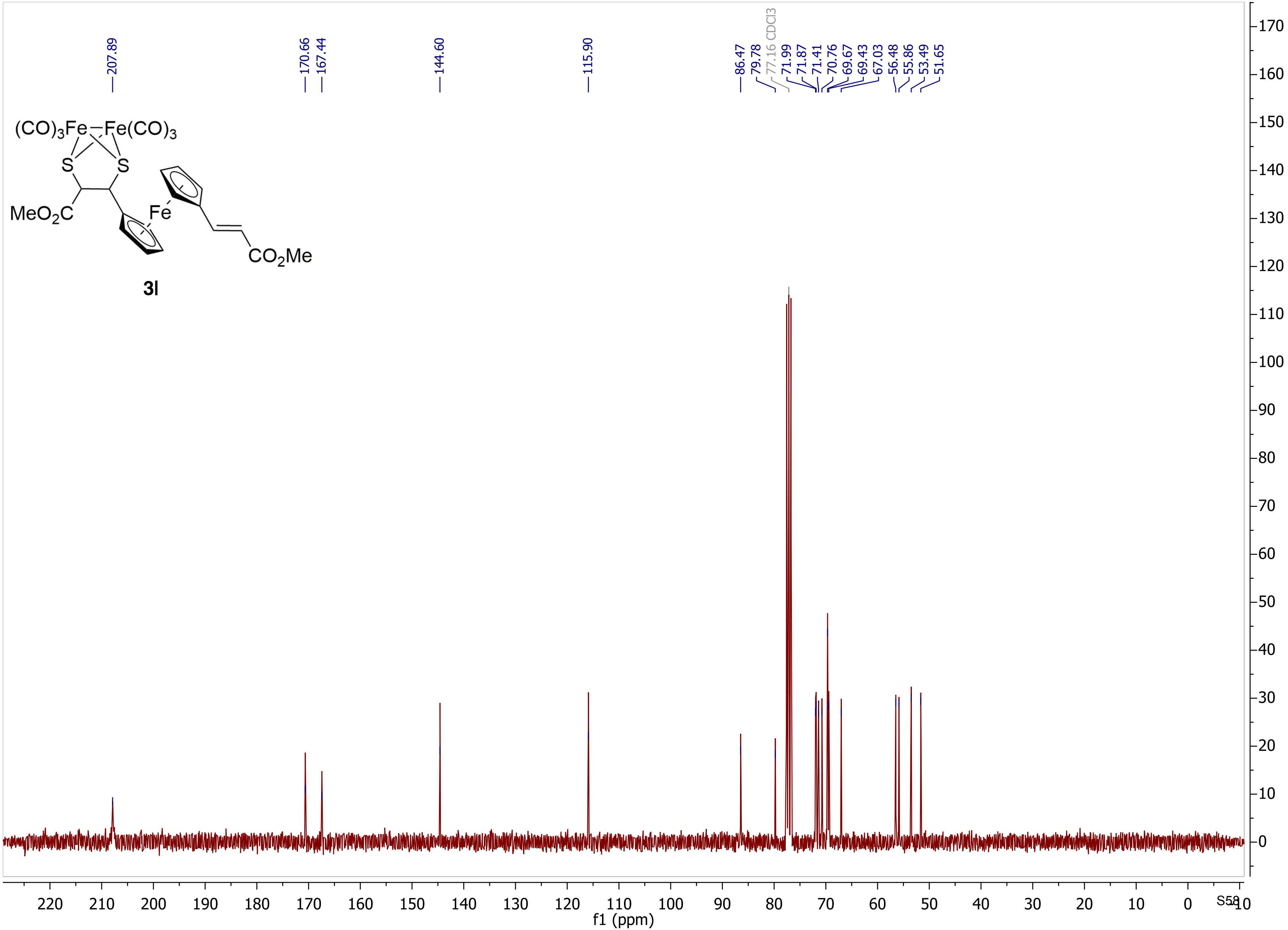
207.89
170.66
167.44
144.60
115.90
86.47
79.78
77.16 CDCl3
71.99
71.87
71.41
70.76
69.67
69.43
67.03
56.48
55.86
53.49
51.65
(CO)3Fe–Fe(CO)3
S
S
MeO2C
Fe
CO2Me
3l
f1 (ppm)

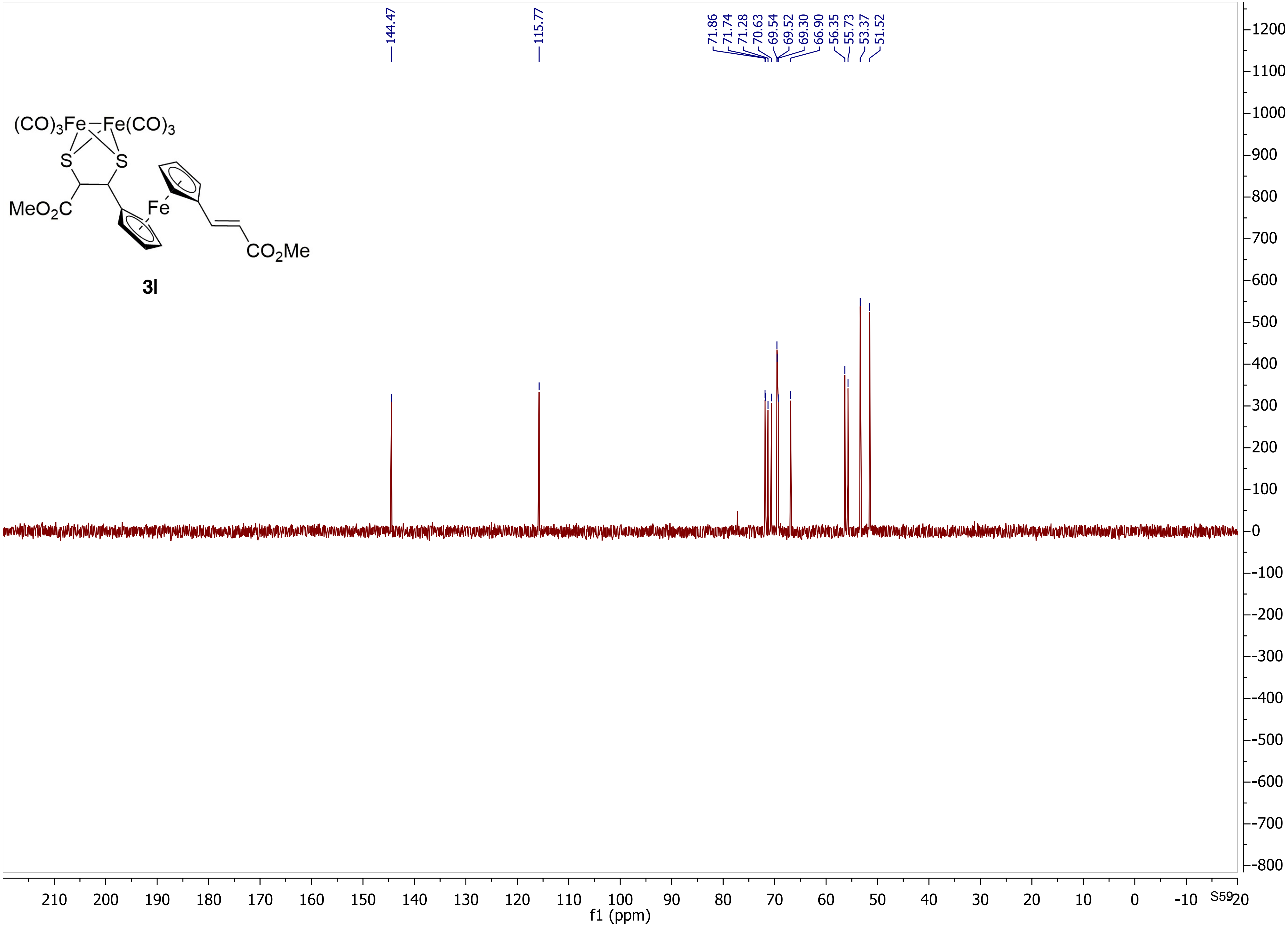
(CO)3Fe—Fe(CO)3
S
S
MeO2C
Fe
CO2Me
3l
144.47
115.77
71.86
71.74
71.28
70.63
69.54
69.52
69.30
66.90
56.35
55.73
53.37
51.52
f1 (ppm)

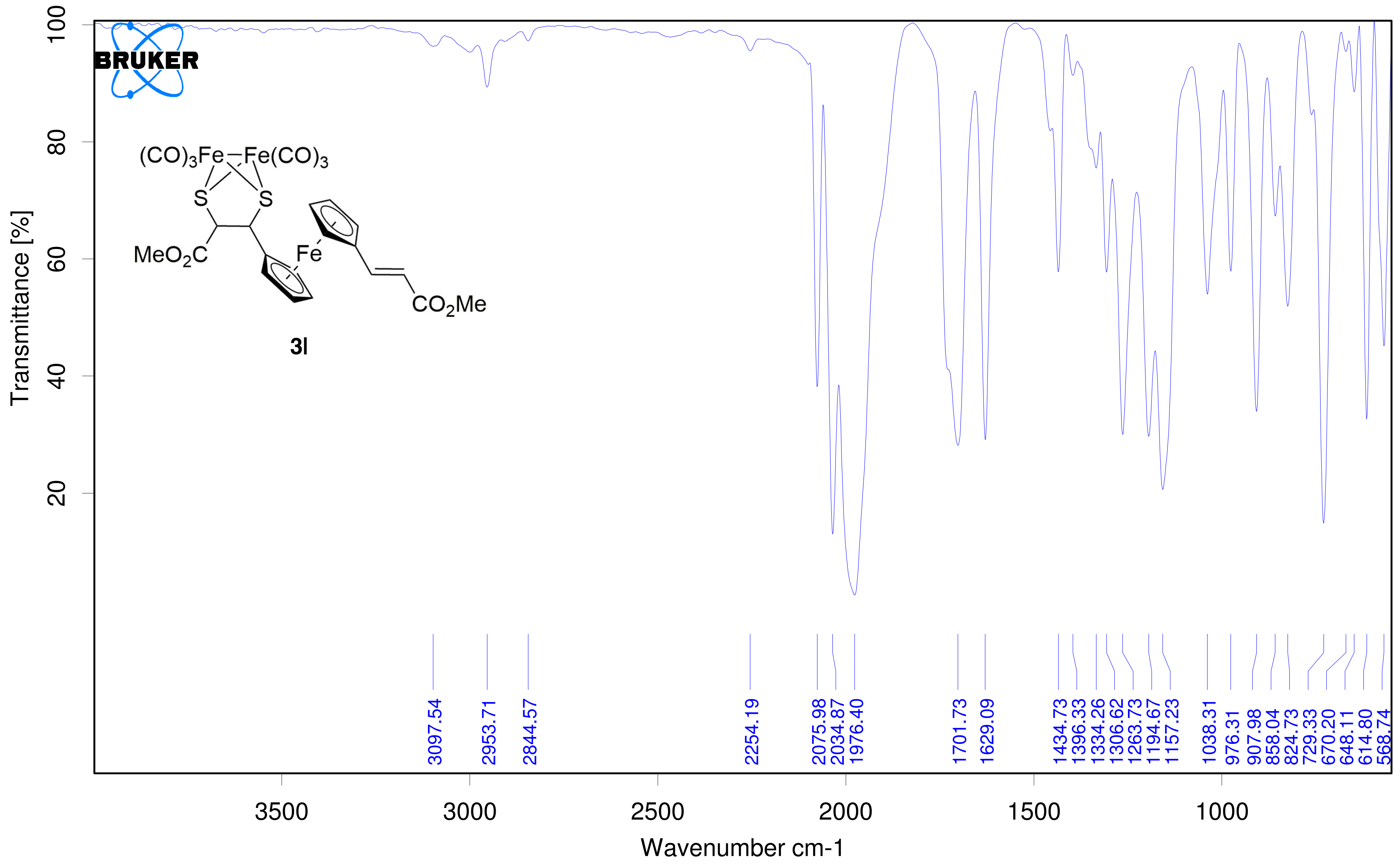
BRUKER
(CO)3Fe−Fe(CO)3
S
S
MeO2C
Fe
CO2Me
3l
Transmittance [%]
100
80
60
40
20
3097.54
2953.71
2844.57
2254.19
2075.98
2034.87
1976.40
1701.73
1629.09
1434.73
1396.33
1334.26
1306.62
1263.73
1194.67
1157.23
1038.31
976.31
907.98
858.04
824.73
729.33
670.20
648.11
614.80
568.74
3500
3000
2500
2000
1500
1000
Wavenumber cm-1

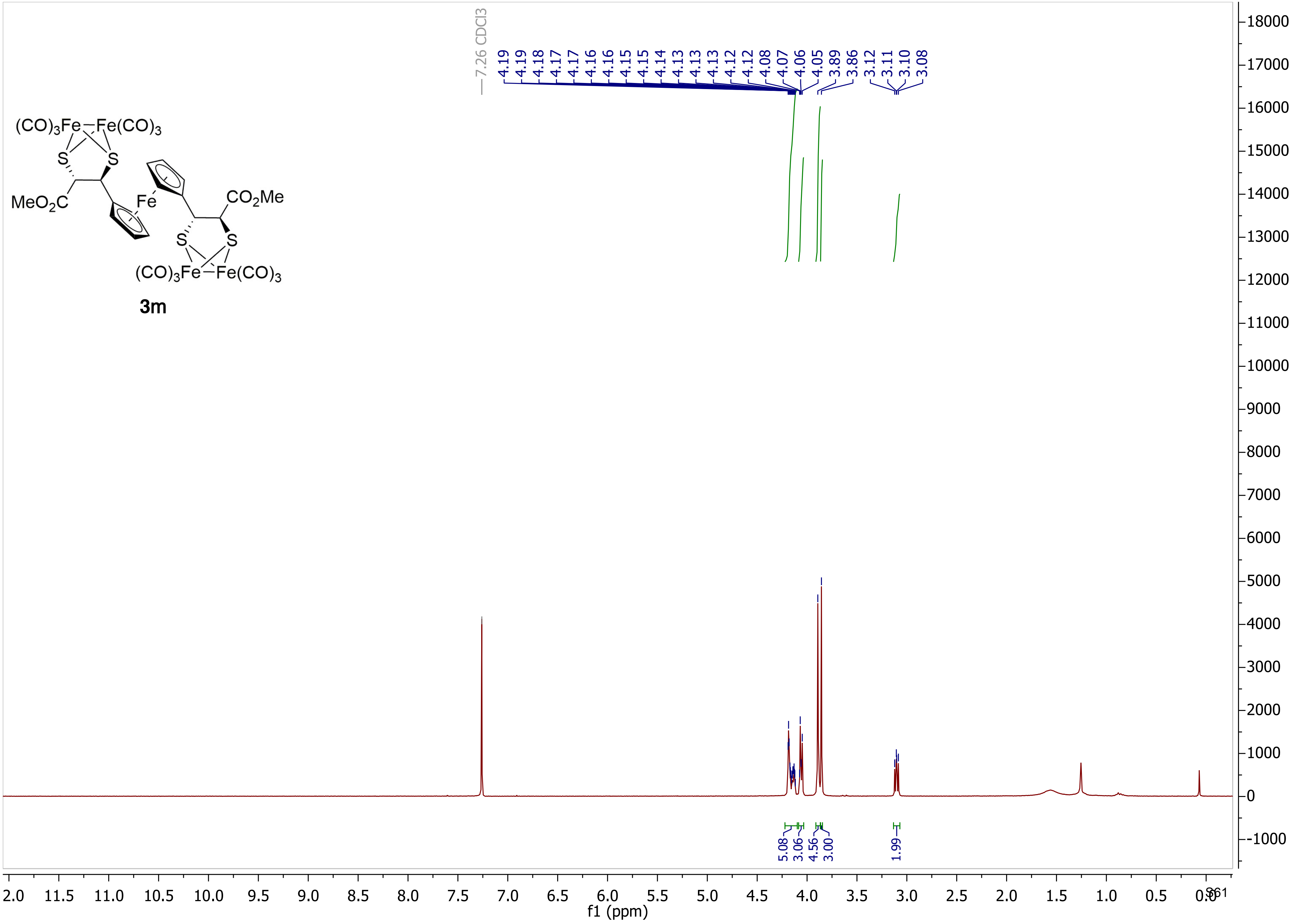
7.26 CDCl3
4.19 4.19 4.18 4.17 4.17 4.16 4.16 4.15 4.15 4.14 4.13 4.13 4.13 4.12 4.12 4.08 4.07 4.06 4.05 3.89 3.86 3.12 3.11 3.10 3.08
(CO)3Fe–Fe(CO)3
S
S
MeO2C
Fe
CO2Me
3m
5.08
3.06
4.56
3.00
1.99
f1 (ppm)

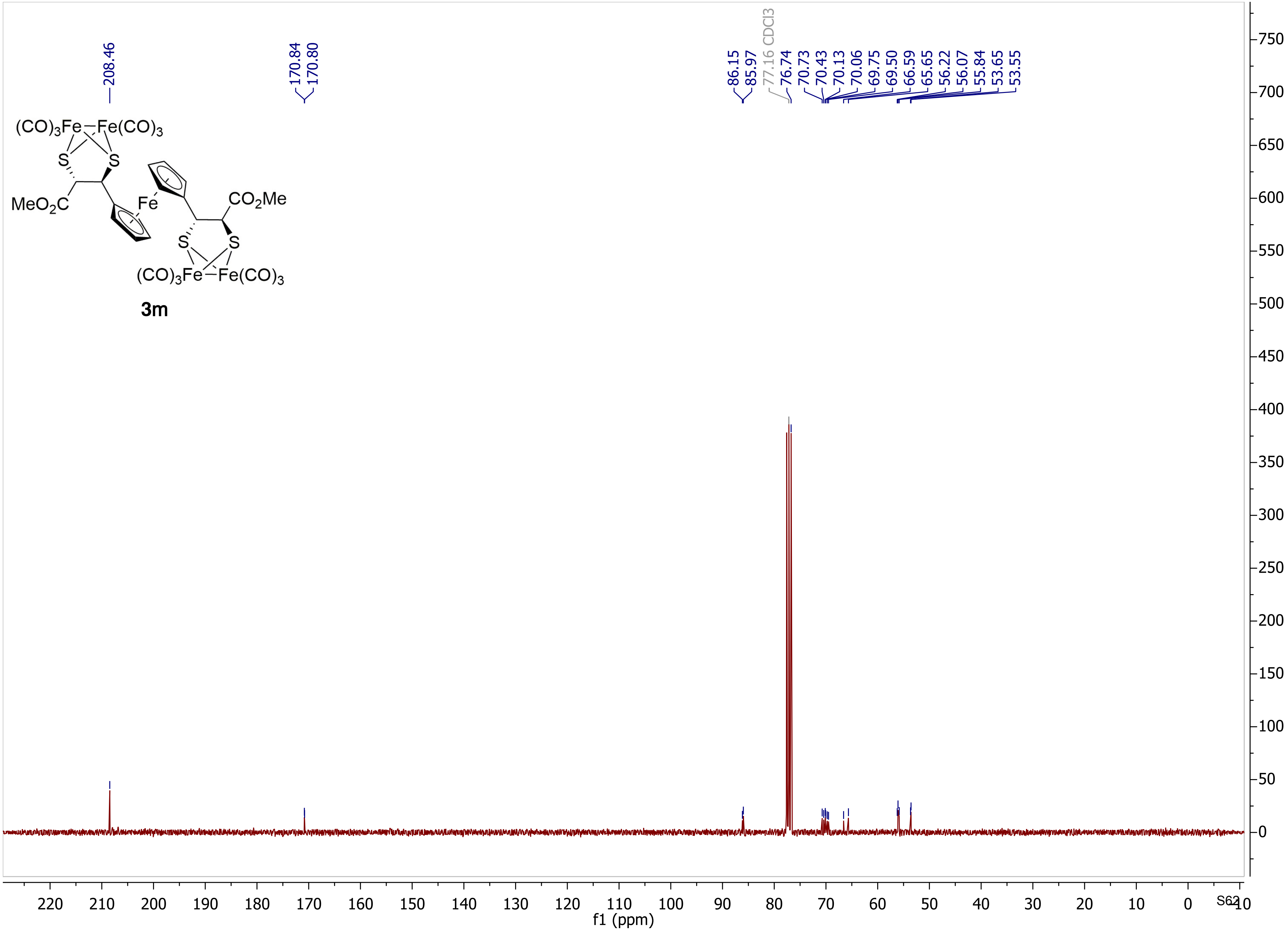
208.46
170.84
170.80
86.15
85.97
77.16 CDCl3
76.74
70.73
70.43
70.13
70.06
69.75
69.50
66.59
65.65
56.22
56.07
55.84
53.65
53.55
3m
f1 (ppm)

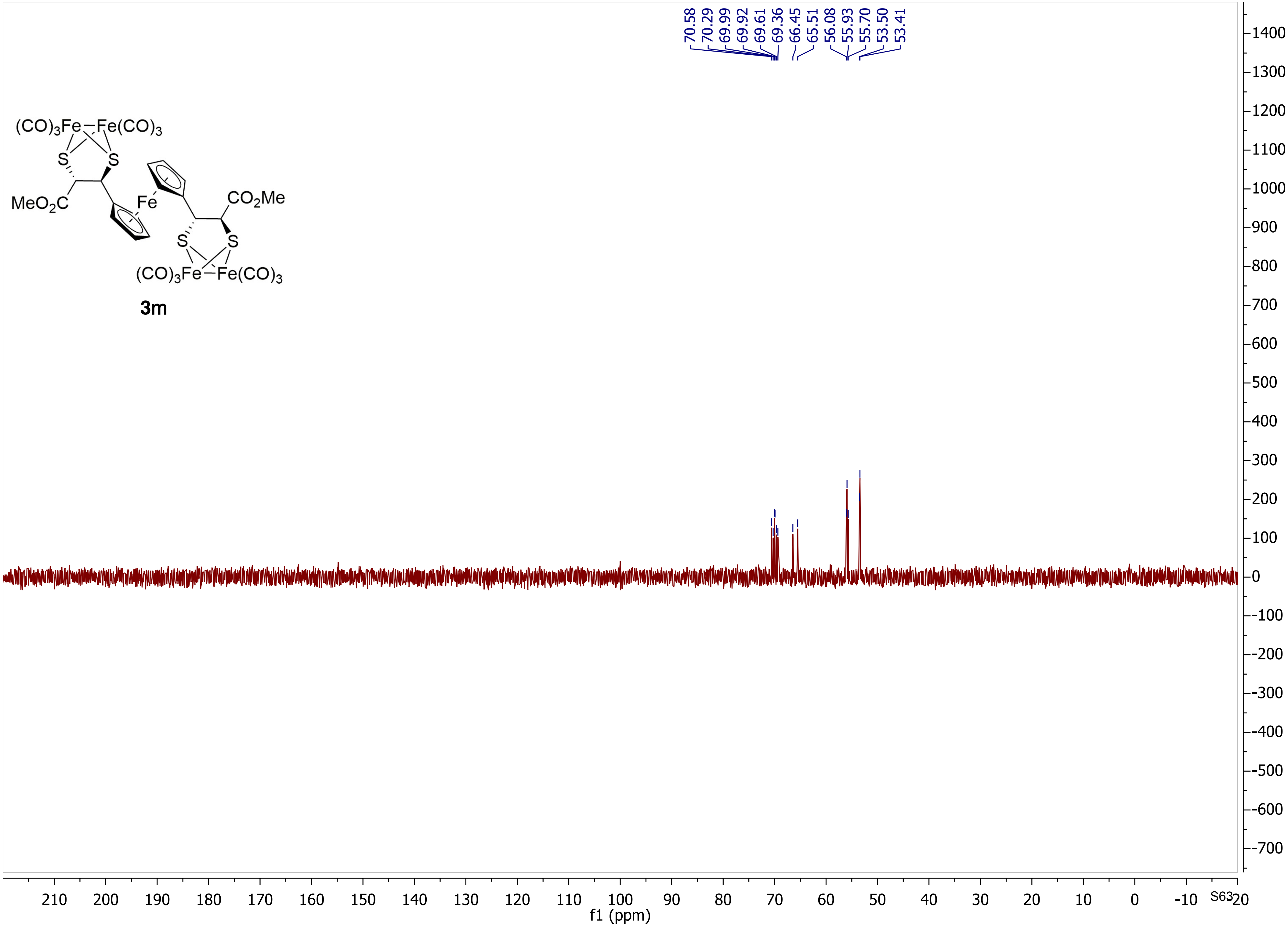
(CO)3Fe
Fe(CO)3
S
S
MeO2C
Fe
CO2Me
S
S
(CO)3Fe
Fe(CO)3

3m

70.58
70.29
69.99
69.92
69.61
69.36
66.45
65.51
56.08
55.93
55.70
53.50
53.41
f1 (ppm)

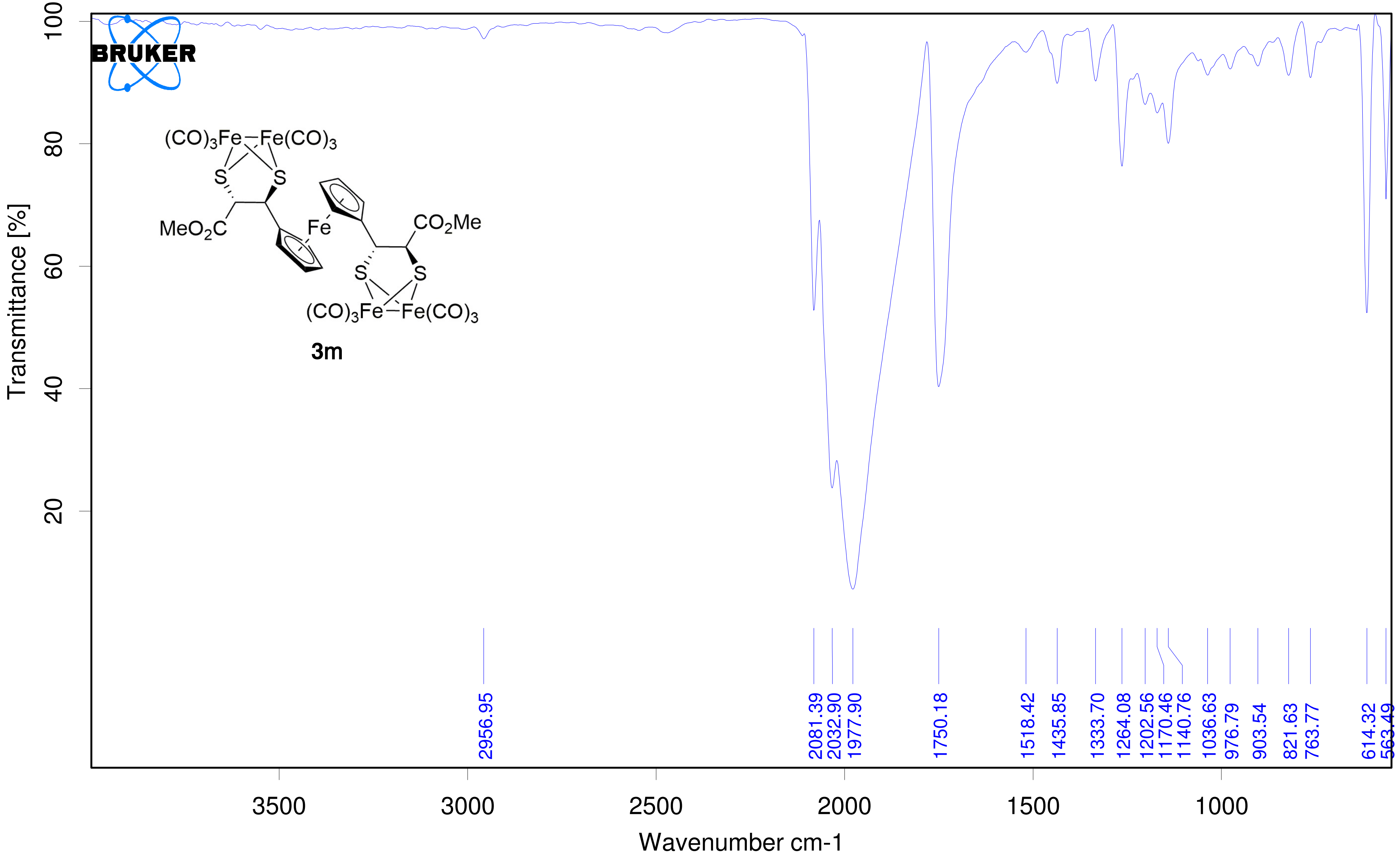

BRUKER
Transmittance [%]
100
80
60
40
20
(CO)3Fe–Fe(CO)3
S
S
MeO2C
Fe
CO2Me
S
S
(CO)3Fe–Fe(CO)3
3m
2956.95
2081.39
2032.90
1977.90
1750.18
1518.42
1435.85
1333.70
1264.08
1202.56
1170.46
1140.76
1036.63
976.79
903.54
821.63
763.77
614.32
563.49
3500
3000
2500
2000
1500
1000
Wavenumber cm-1

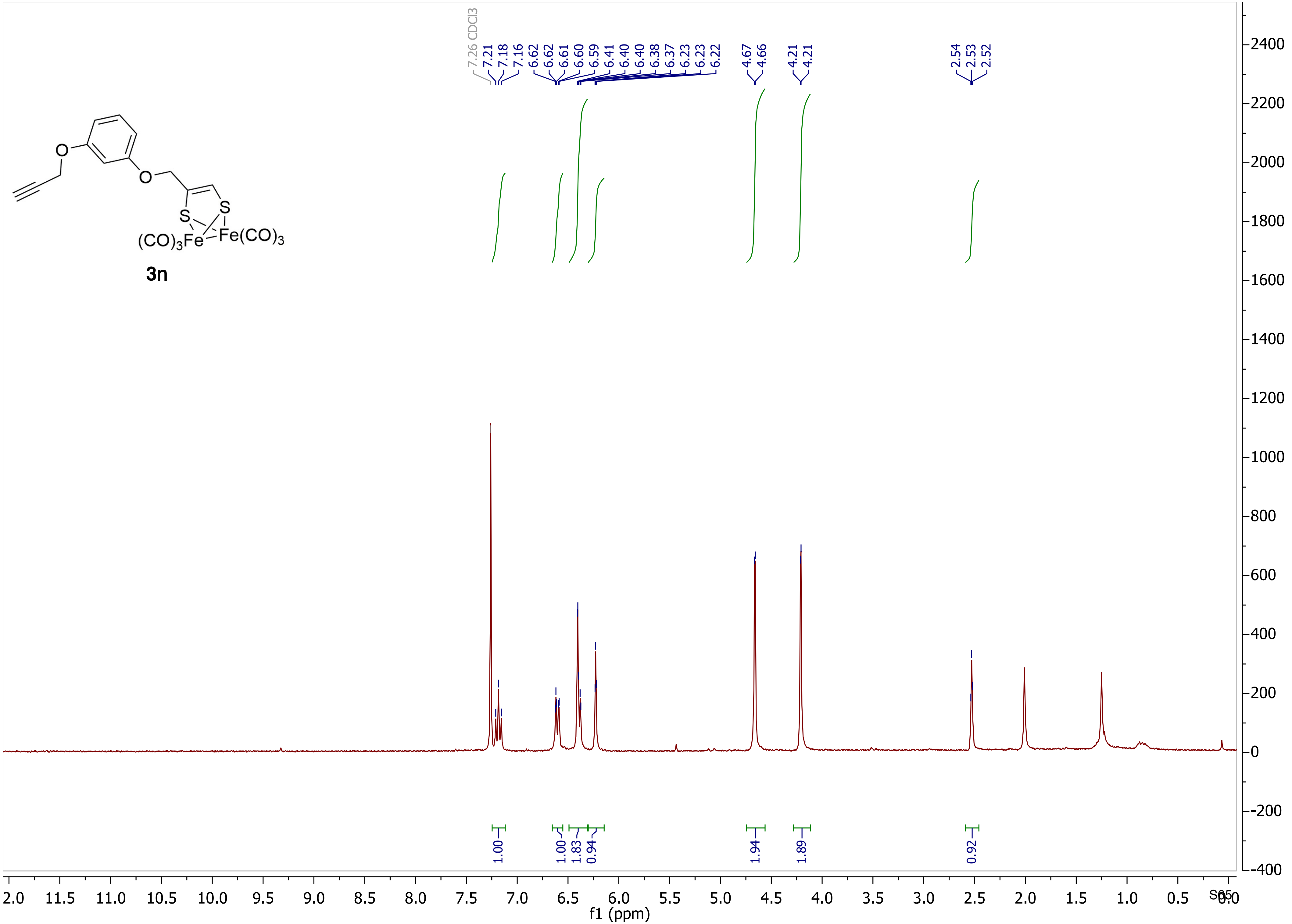

7.26 CDCl3
7.21
7.18
7.16
6.62
6.62
6.61
6.60
6.59
6.41
6.40
6.40
6.38
6.37
6.23
6.23
6.22
4.67
4.66
4.21
4.21
2.54
2.53
2.52
O
O
S
S
(CO)3Fe
Fe(CO)3
3n
1.00
1.00
1.83
0.94
1.94
1.89
0.92
f1 (ppm)

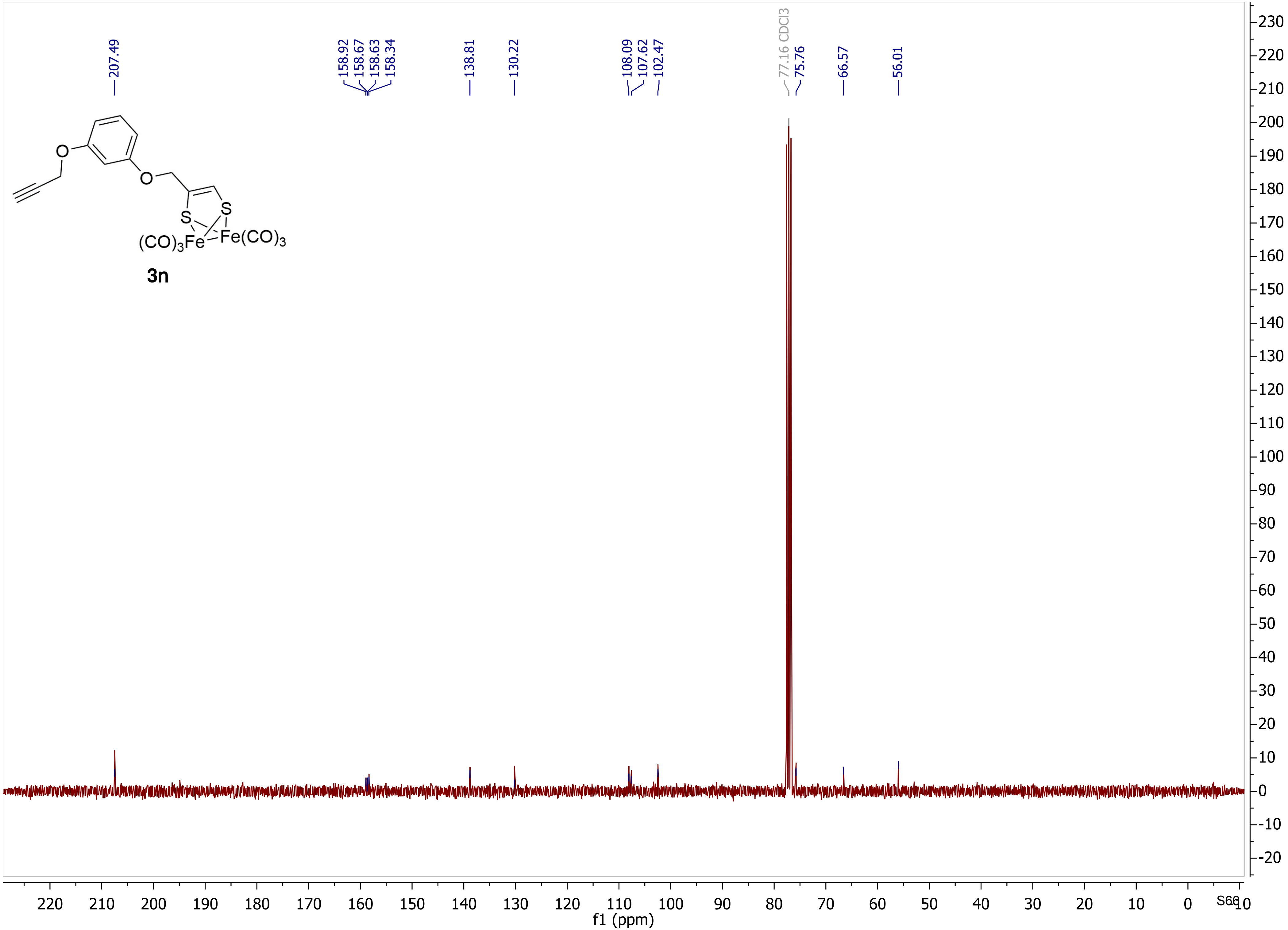

207.49
158.92
158.67
158.63
158.34
138.81
130.22
108.09
107.62
102.47
77.16 CDCl3
75.76
66.57
56.01
3n
f1 (ppm)

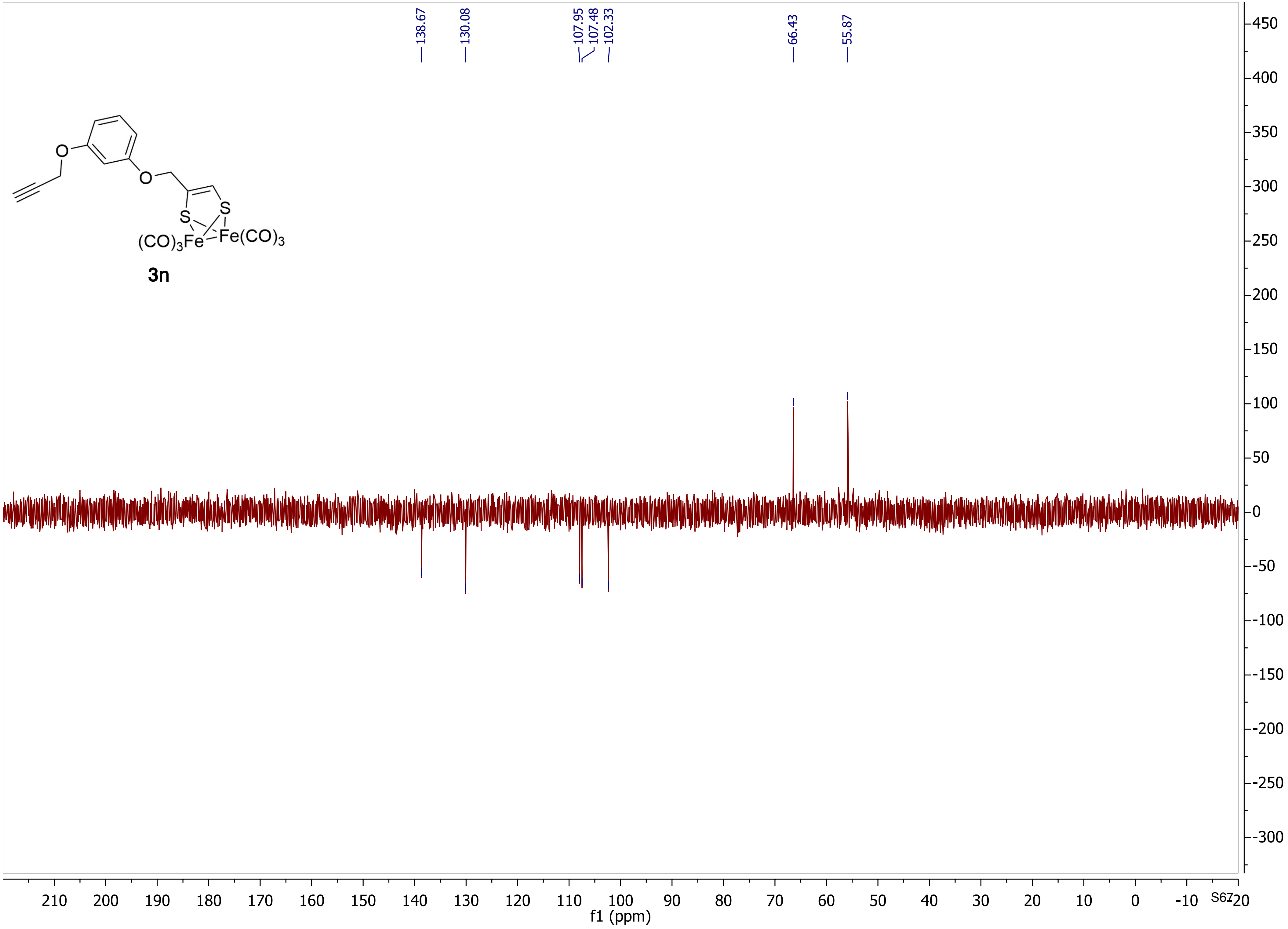
138.67
130.08
107.95
107.48
102.33
66.43
55.87
O
O
S
S
(CO)3Fe
Fe(CO)3
3n
f1 (ppm)

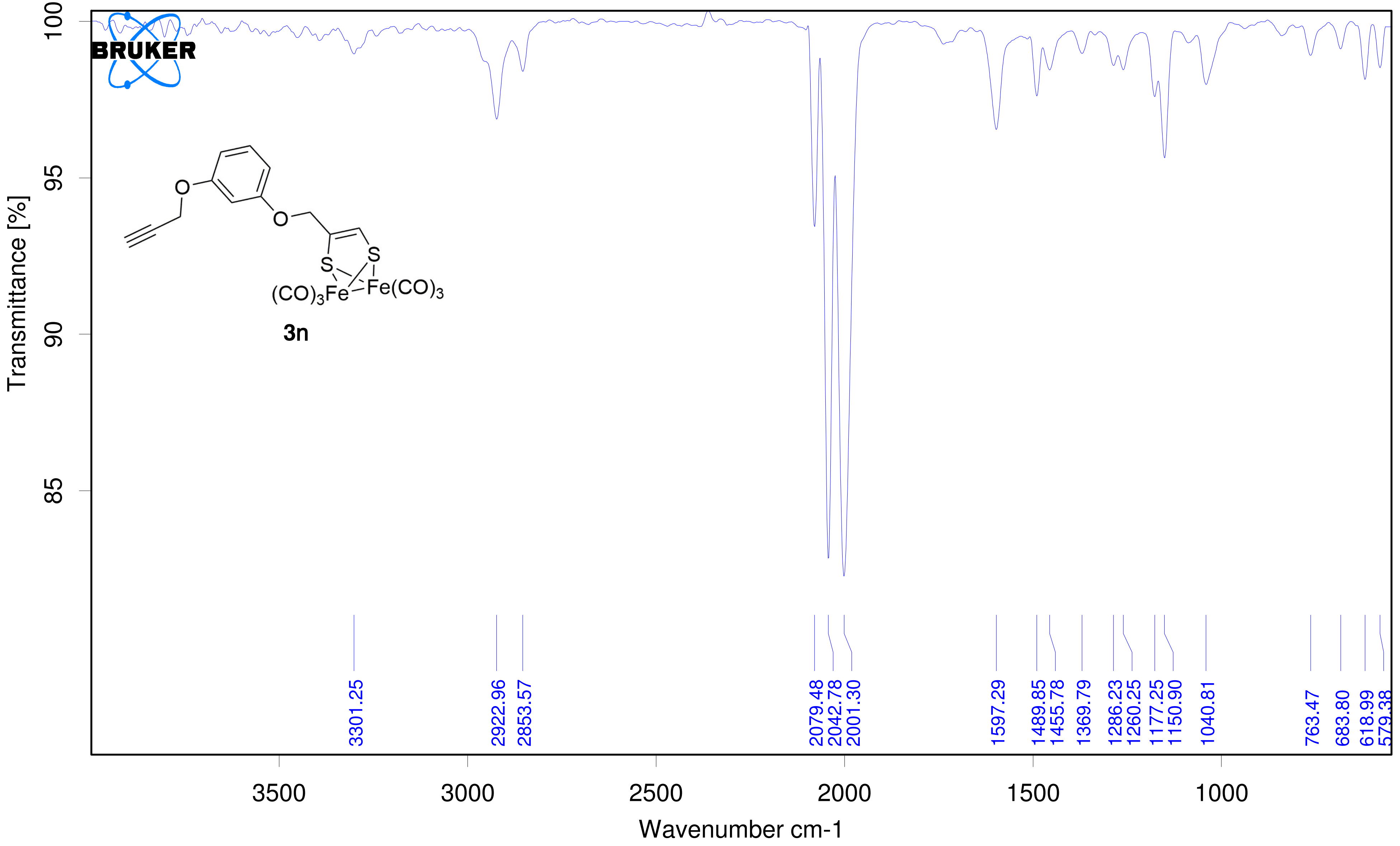

Transmittance [%]
100
95
90
85
3500
3000
2500
2000
1500
1000
Wavenumber cm-1
3301.25
2922.96
2853.57
2079.48
2042.78
2001.30
1597.29
1489.85
1455.78
1369.79
1286.23
1260.25
1177.25
1150.90
1040.81
763.47
683.80
618.99
579.38
O
O
S
S
(CO)3Fe
Fe(CO)3
3n

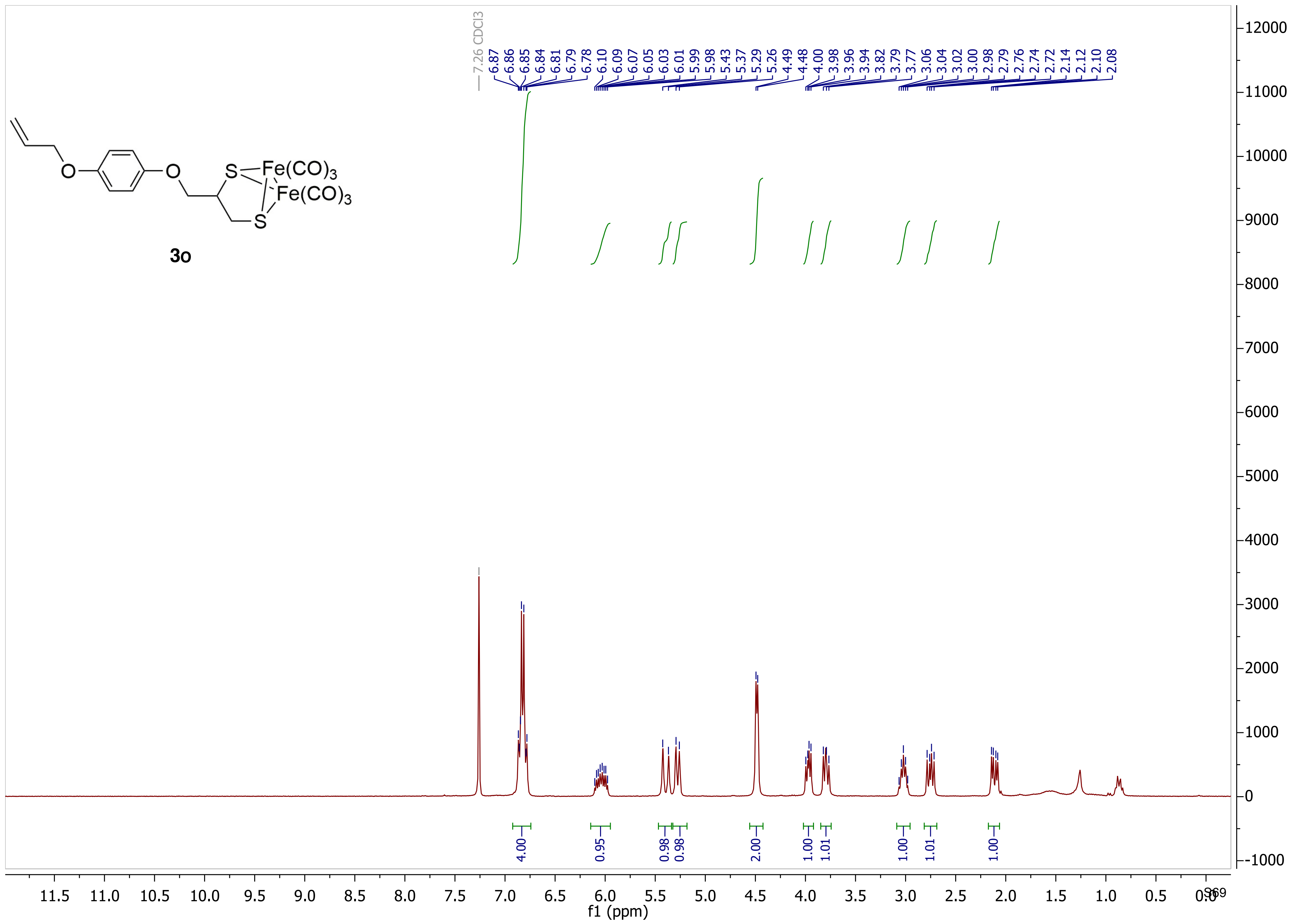

3o
7.26 CDCl3
6.87
6.86
6.85
6.84
6.81
6.79
6.78
6.10
6.09
6.07
6.05
6.03
6.01
5.99
5.98
5.43
5.37
5.29
5.26
4.49
4.48
4.00
3.98
3.96
3.94
3.82
3.79
3.77
3.06
3.04
3.02
3.00
2.98
2.79
2.76
2.74
2.72
2.14
2.12
2.10
2.08
4.00
0.95
0.98
0.98
2.00
1.00
1.01
1.00
1.01
1.00
f1 (ppm)

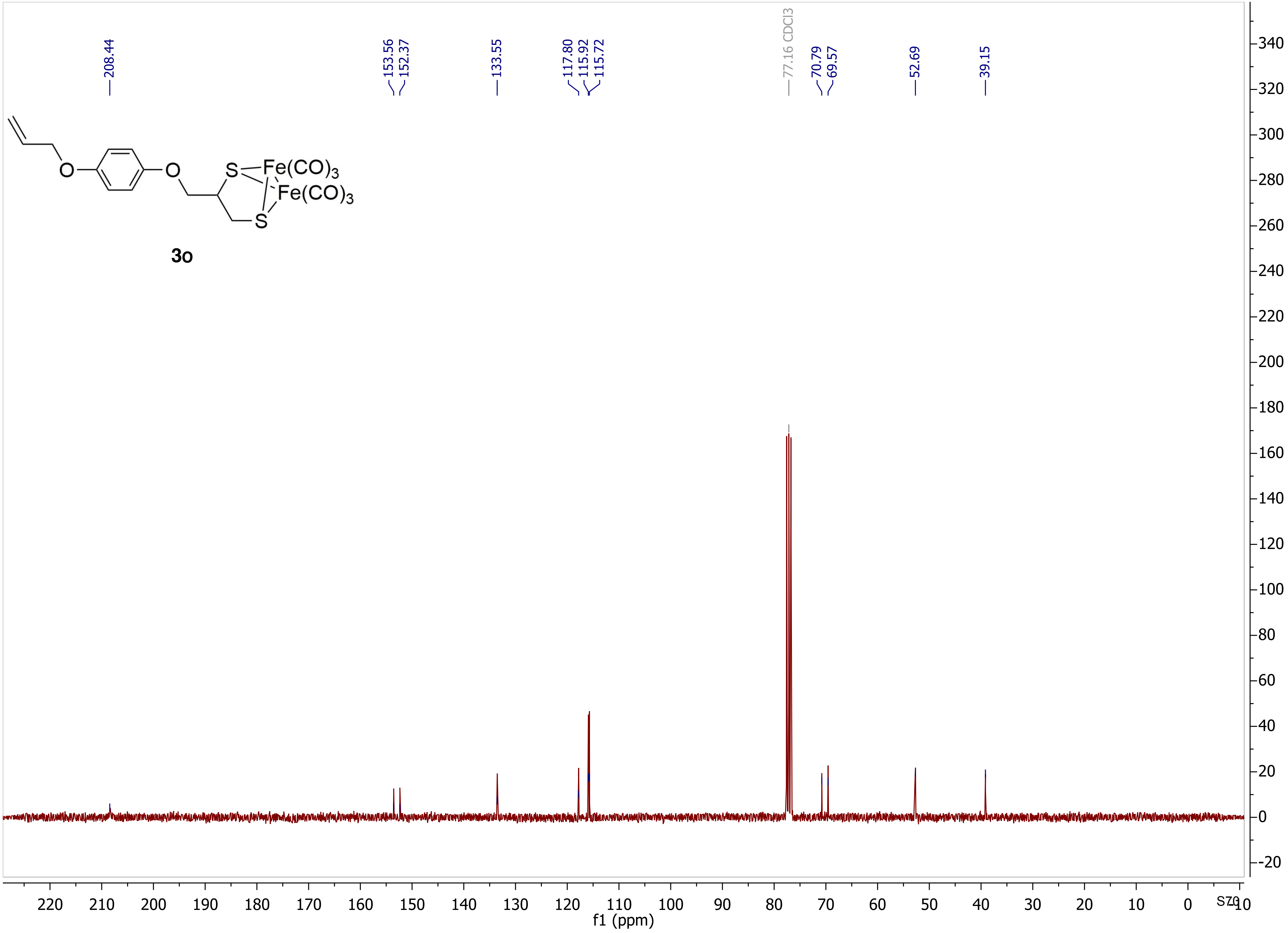

208.44
153.56
152.37
133.55
117.80
115.92
115.72
77.16 CDCl3
70.79
69.57
52.69
39.15
O
O
S
Fe(CO)3
Fe(CO)3
S
3o
220
210
200
190
180
170
160
150
140
130
120
110
100
90
80
70
60
50
40
30
20
10
0
-10
f1 (ppm)
340
320
300
280
260
240
220
200
180
160
140
120
100
80
60
40
20
0
-20
SZ6

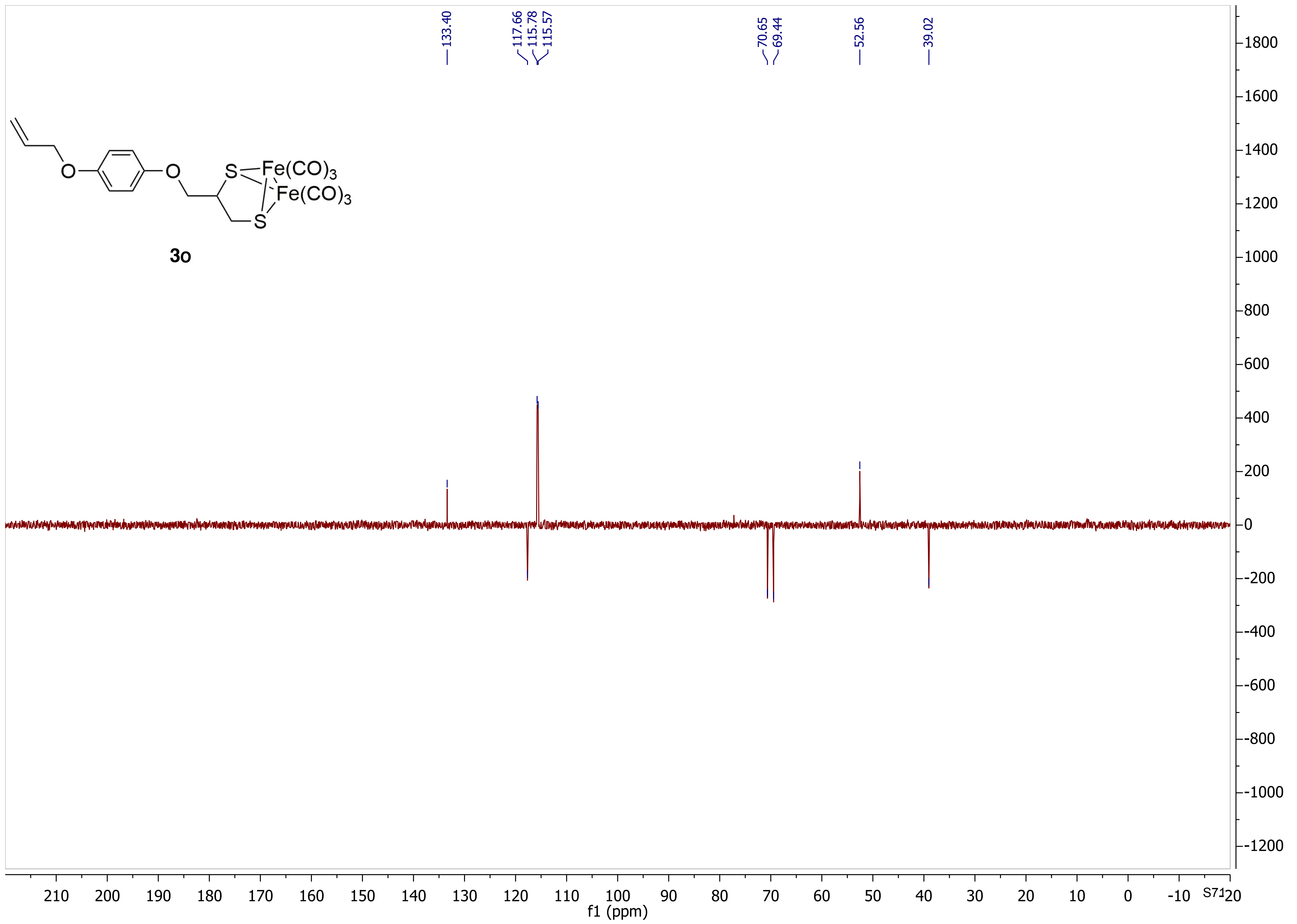
133.40
117.66
115.78
115.57
70.65
69.44
52.56
39.02
Fe(CO)3
Fe(CO)3
3o
f1 (ppm)

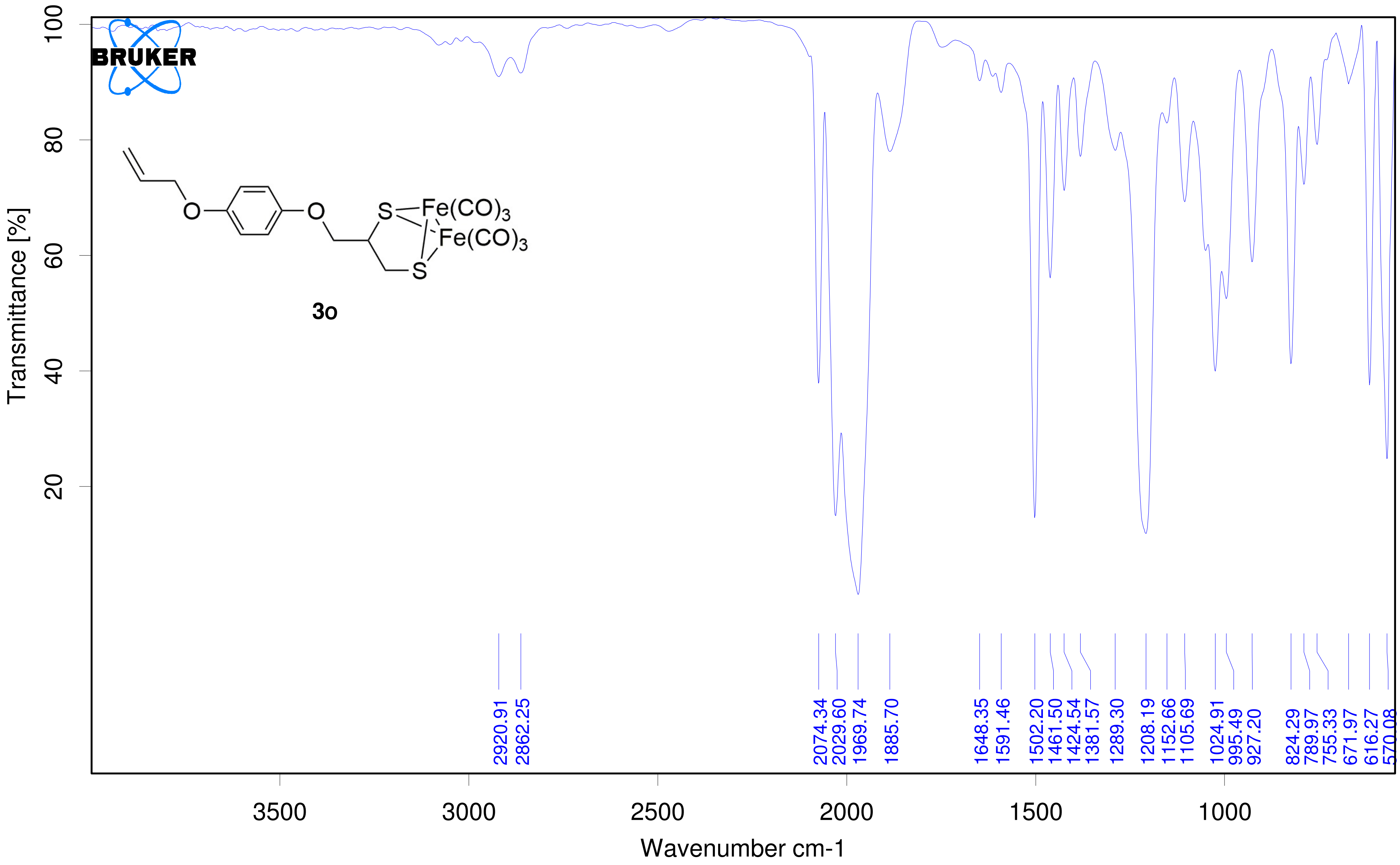
BRUKER
Transmittance [%]
100
80
60
40
20
3o
2920.91
2862.25
2074.34
2029.60
1969.74
1885.70
1648.35
1591.46
1502.20
1461.50
1424.54
1381.57
1289.30
1208.19
1152.66
1105.69
1024.91
995.49
927.20
824.29
789.97
755.33
671.97
616.27
570.08
3500
3000
2500
2000
1500
1000
Wavenumber cm-1

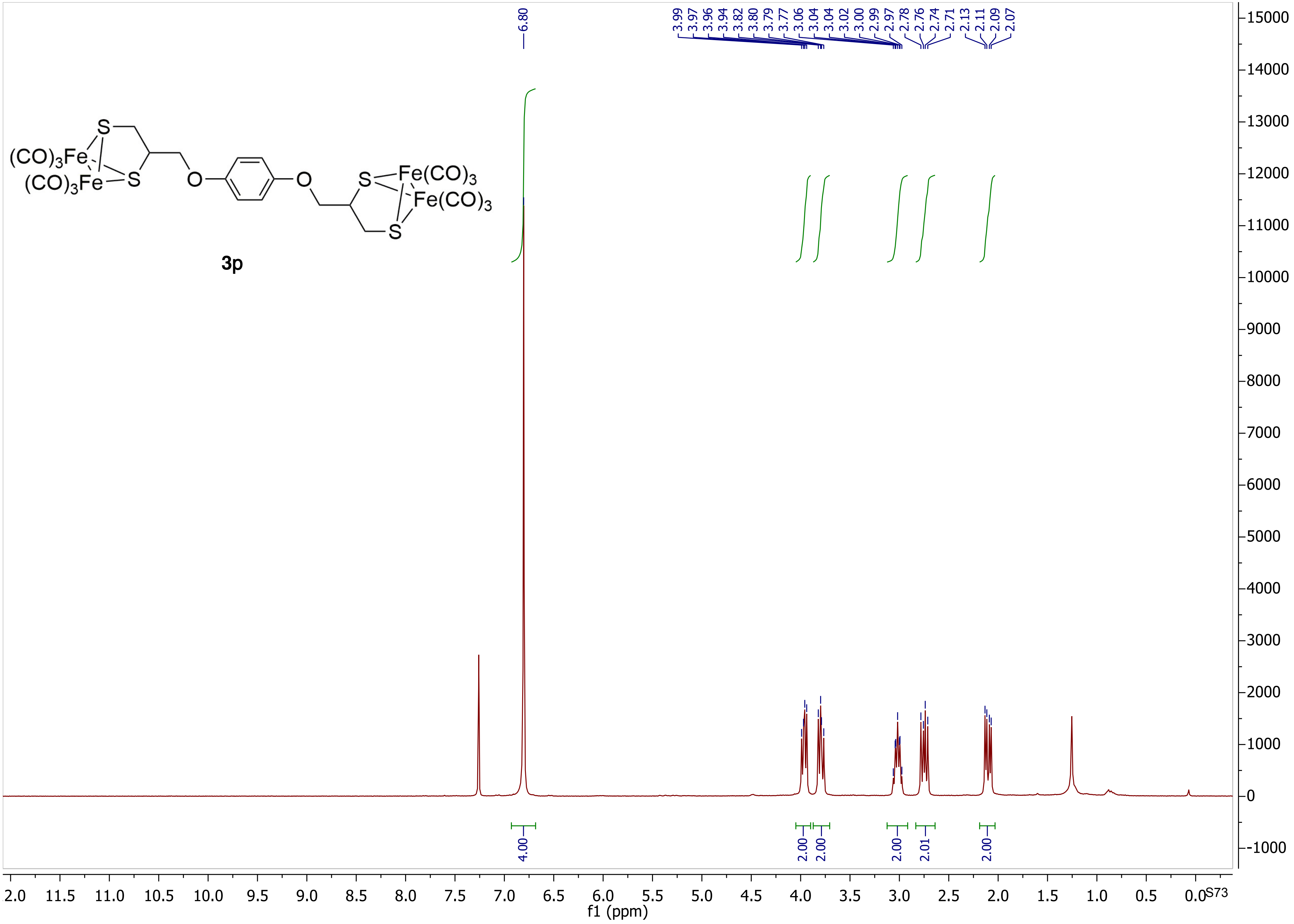
(CO)3Fe
(CO)3Fe
Fe(CO)3
Fe(CO)3
S
O
3p
6.80
3.99 3.97 3.96 3.94 3.82 3.80 3.79 3.77 3.06 3.04 3.04 3.02 3.00 2.99 2.97 2.78 2.76 2.74 2.71 2.13 2.11 2.09 2.07
4.00
2.00
2.00
2.00
2.01
2.00
f1 (ppm)

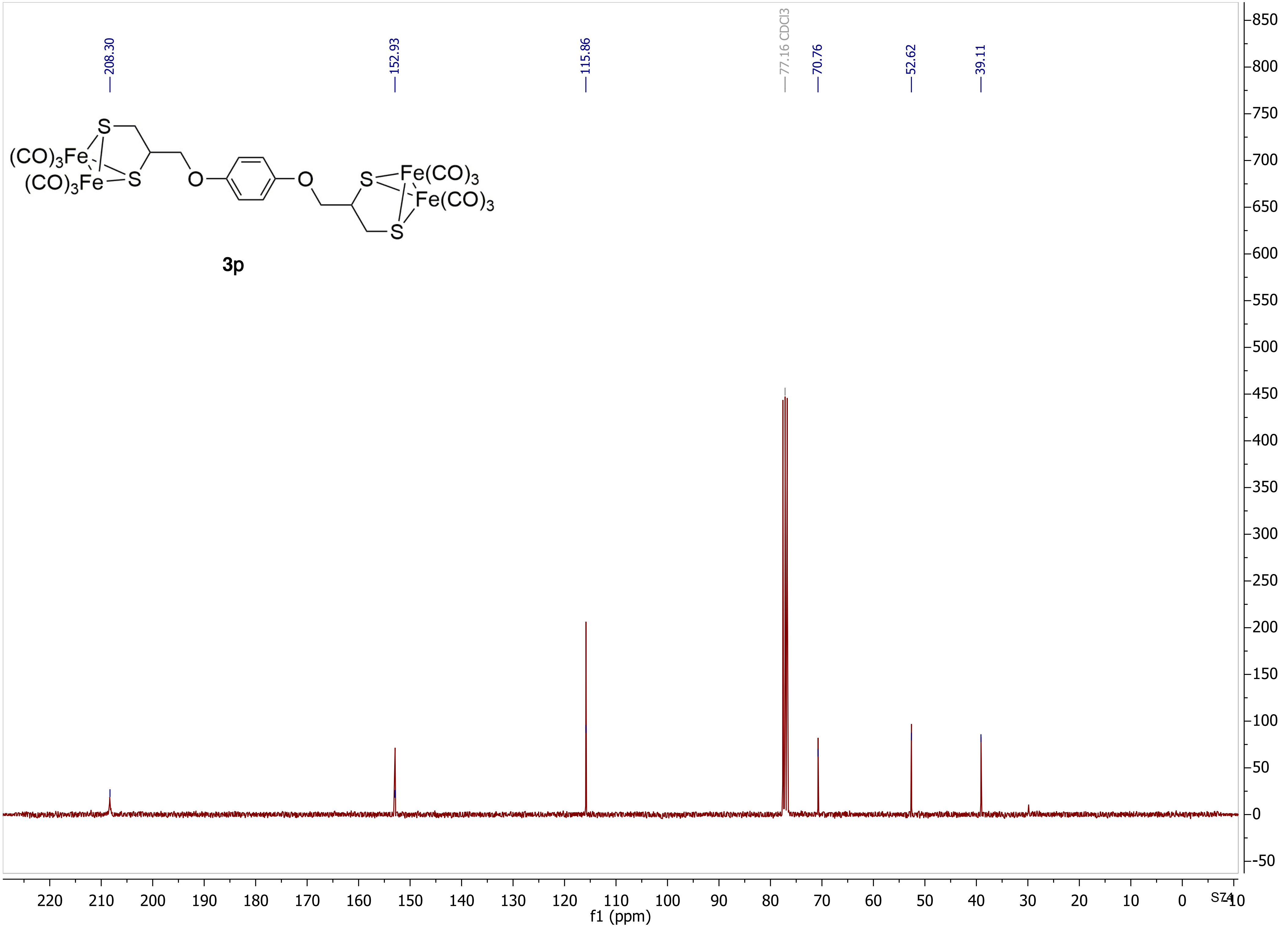
208.30
152.93
115.86
77.16 CDCl3
70.76
52.62
39.11
(CO)3Fe
(CO)3Fe
S
S
O
O
S
S
Fe(CO)3
Fe(CO)3
3p
850
800
750
700
650
600
550
500
450
400
350
300
250
200
150
100
50
0
-50
220 210 200 190 180 170 160 150 140 130 120 110 100 90 80 70 60 50 40 30 20 10 0 -10
f1 (ppm)

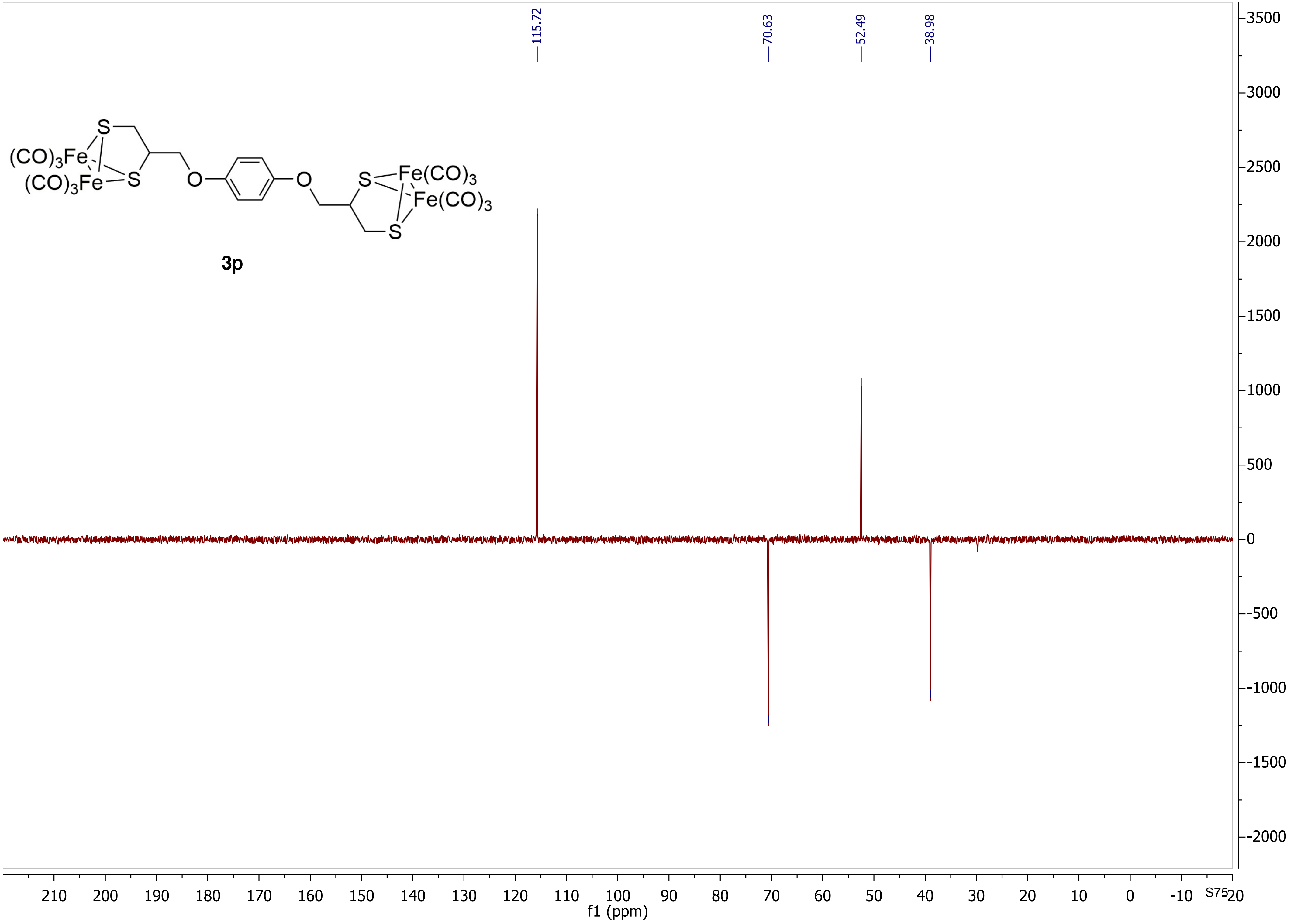
115.72
70.63
52.49
38.98
3p
f1 (ppm)

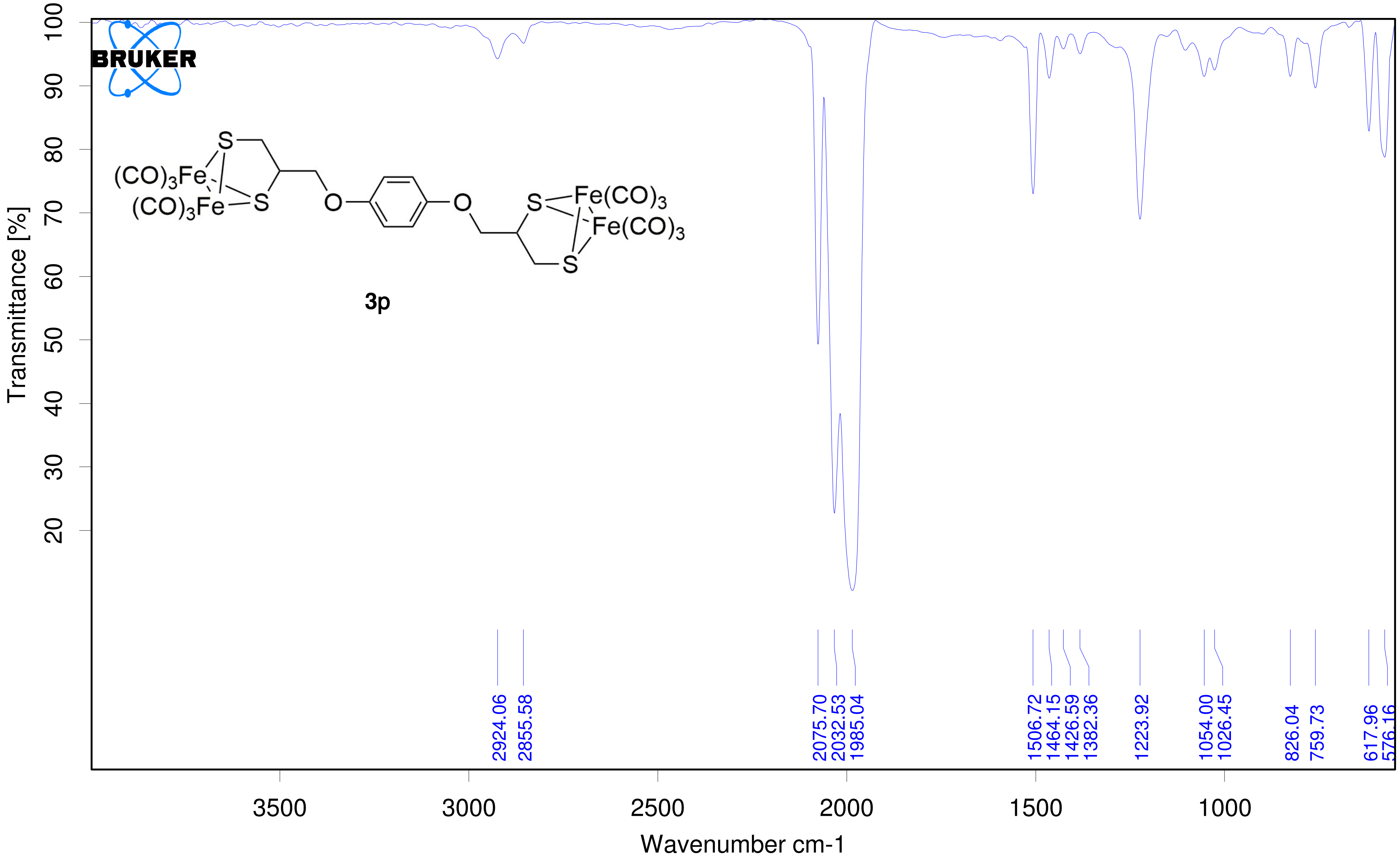

BRUKER
(CO)3Fe
(CO)3Fe
S
S
O
O
S
S
Fe(CO)3
Fe(CO)3
3p
Transmittance [%]
100
90
80
70
60
50
40
30
20
2924.06
2855.58
2075.70
2032.53
1985.04
1506.72
1464.15
1426.59
1382.36
1223.92
1054.00
1026.45
826.04
759.73
617.96
576.16
3500
3000
2500
2000
1500
1000
Wavenumber cm-1
